# Phase I trial of hydroxychloroquine to enhance palbociclib and letrozole efficacy in ER+/HER2− breast cancer

**DOI:** 10.1038/s41523-025-00722-1

**Published:** 2025-01-26

**Authors:** Akshara Singareeka Raghavendra, Nicole M. Kettner, Danielle Kwiatkowski, Senthil Damodaran, Yan Wang, David Ramirez, Dan S. Gombos, Kelly K. Hunt, Yu Shen, Khandan Keyomarsi, Debu Tripathy

**Affiliations:** 1 Department of Breast Medical Oncology, The University of Texas MD Anderson Cancer Center, Houston, TX USA; 2 Department of Experimental Radiation, The University of Texas MD Anderson Cancer Center, Houston, TX USA; 3 Department of Head and Neck Surgery, The University of Texas MD Anderson Cancer Center, Houston, TX USA; 4 Department of Breast Surgical Oncology, The University of Texas MD Anderson Cancer Center, Houston, TX USA; 5 Department of Biostatistics, The University of Texas MD Anderson Cancer Center, Houston, TX USA

**Keywords:** Breast cancer, Breast cancer, Phase I trials, Breast cancer

## Abstract

Endocrine therapy with CDK4/6 inhibitors is standard for estrogen receptor-positive, HER2−negative metastatic breast cancer (ER+/HER2− MBC), yet clinical resistance develops. Previously, we demonstrated that low doses of palbociclib activate autophagy, reversing initial G1 cell cycle arrest, while high concentrations induce off-target senescence. The autophagy inhibitor hydroxychloroquine (HCQ) induced on-target senescence at lower palbociclib doses. We conducted a phase I trial (NCT03774472 registered in ClinicalTrials.gov on 8/20/2018) of HCQ (400, 600, 800 mg/day) with palbociclib (75 mg/day continuous) and letrozole, using a 3 + 3 design. Primary objectives included safety, tolerability, and determining the recommended phase 2 dose (RP2D) of HCQ. Secondary objectives included tumor response and biomarker analysis. Fourteen ER+/HER2− MBC patients were evaluable [400 mg (*n* = 4), 600 mg (*n* = 4), 800 mg (*n* = 6)]. Grade 3 adverse events (AEs) included hematological (3 at 800 mg), skin rash (2 at 600 mg), and anorexia (1 at 400 mg), with no serious AEs. The best responses were partial (2), stable (11), and progression (1). Tumor reductions ranged from 11% to 30%, with one 55% increase. The two partial responders sustained tumor size reductions of 30% to 55% over an extended treatment period, lasting nearly 300 days. Biomarker analysis in responders demonstrated significant decreases in Ki67, Rb, and nuclear cyclin E levels and increases in autophagy markers p62 and LAMP1, suggesting a correlation between these biomarkers and treatment response. This phase I study demonstrated that HCQ is safe and well-tolerated and the RP2D was established at 800 mg/day with continuous low-dose palbociclib (75 mg/day) and letrozole (2.5 mg/day). These findings suggest that adding HCQ could potentially enhance the efficacy of low-dose palbociclib and standard letrozole therapy, pending verification in larger randomized studies.

## Introduction

Hormone receptor-positive (HR+) and human epidermal growth factor receptor 2 (HER2)-negative breast cancer represents the largest subtype of breast cancer, accounting for 75% of cases in the U.S., including the largest number of breast cancer deaths annually^1,2^. It is estimated that 155,000 patients are living with metastatic breast cancer (MBC) in the US, and that 35,000 new cases of metastatic HR+/HER2− breast cancer are diagnosed either de novo (presenting with metastatic disease), or as a result of recurrence of previously treated disease, with 30,000 deaths expected each year^2,3^. While survival rates for breast cancer have been improving due to earlier detection and newer treatments, the trends for MBC are lagging behind, with 5-year survival rates for HR+/HER2− breast cancer of 34%^3,4^.

The use of endocrine therapy (ET) as initial treatment for HR+/HER2− MBC has become more common, as studies show chemotherapy is not necessarily superior in terms of survival, especially compared with ET plus cyclin-dependent kinase 4 and 6 inhibitors (CDK4/6i)^5–10^. However, early progression within 8 weeks occurs in ~20% of patients with ET/CDK4/6i first-line therapy, and later-line treatments typically yield progressively shorter response times^11,12^. There is a need to better understand mechanisms of intrinsic resistance and early adaptive responses to ET/CDK4/6i therapy to extend breast cancer control time in the first-line setting.

The cell cycle apparatus has long been explored as a target in cancer treatment as its dysregulation is a hallmark of cancer, with multiple mutations ascribed to positive and negative regulatory components^13^. The development of specific small molecule inhibitors to CDK4/6, kinases that activate both G1/S transition and cross-talk with other oncogenic pathways, demonstrated the expected preclinical effects, particularly in HR+ breast cancer cell line models and exhibited acceptable toxicity in early-phase clinical trials^14^. In 2015, the approval of palbociclib augured a new era of targeted therapy when added to the AI letrozole, and a doubling of progression-free survival was observed^15^. Subsequently, several phase III randomized trials showed a similar impact with palbociclib, and other CDK4/6i ribociclib and abemaciclib combined with AI in first-line as well as with fulvestrant in first or second-line therapy^7,12,16–20^. Longer follow-up has now demonstrated improvement in overall survival (OS) with ribociclib and abemaciclib^21–24^. The use of CDK4/6i is now a standard and preferred option in the first-line setting for patients with advanced HR+/HER2− breast cancer^25^.

However, treatment with CDK4/6is does introduce new toxicities that require additional monitoring and may infrequently cause significant morbidity. For example, in the first-line setting, 66% of patients on palbociclib experience grade 3 and 4 neutropenia, and adverse events (AEs) necessitated a palbociclib dose reduction in 36% and discontinuation in 7.4% of patients^16^. Additionally, palbociclib must be dosed with a 7-day break, which was shown to be associated with a proliferative burst in a neoadjuvant serial biopsy study^26^. Also, a precise biological mechanism of palbociclib’s action is still unknown, and there are no known independent biomarkers to predict response and/or resistance to palbociclib. Many patients do not respond to therapy, and most importantly, virtually all eventually develop resistance. Biomarkers that may reduce response to ET and CDK4/6i including estrogen receptor-alpha (*ESR1*) and phosphatidylinositol-4,5-Bisphosphate 3-Kinase Catalytic Subunit Alpha (*PIK3CA)* mutations, loss of the retinoblastoma protein function, high proliferative antigen Ki67 expression, *cyclin D1* gene amplification or loss of the cyclin-dependent kinase inhibitor^19^ have not correlated with the relative benefits of palbociclib-associated response or time to progression^11,27,28^.

Previously, we reported that breast cancer cells activate autophagy in response to low doses of palbociclib and that the combination with autophagy inhibitors (i.e., hydroxychloroquine) induces irreversible growth inhibition and senescence in vitro, and diminish growth of cell line and patient-derived xenograft tumors in vivo^29^. The low concentrations of palbociclib are on-target and specific inhibitors of CDK4 and CDK6, as shown with small interfering RNA (siRNA) knockdown of CDK4 and CDK6. High concentrations of palbociclib induce senescence, but these are not related to CDK4/6 inhibition and are off-target effects of the drug. These results show that autophagy inhibition significantly improves the efficacy of low-dose (on-target) palbociclib in vivo and facilitates irreversible tumor growth inhibition- suggesting that palbociclib activates the autophagy pathway to protect HR+ breast cancer cells from palbociclib-induced senescence, and inhibition of autophagy sensitizes cells to lower doses of palbociclib in vitro and in vivo^29^.

Autophagy is a cellular process known for its dual role in tumor suppression^30^ and promotion^31^, as well as in contributing to drug resistance by clearing damaged cellular components and promoting survival^32^. Various inhibitors of autophagy have been investigated to enhance cancer therapy. These include: Bafilomycin A1, which inhibits the vacuolar-type H + -ATPase, disrupting lysosomal acidification and autophagic flux^33^; 3-Methyladenine (3-MA), an inhibitor of class III PI3K necessary for early autophagy stages^34^; Wortmannin- another PI3K inhibitor that hinders autophagosome formation^35^ and Spautin-1, inhibiting deubiquitinating enzymes USP10 and USP13, which target the class III PI3K complex^36^. Chloroquine (CQ) and hydroxychloroquine (HCQ) are the most extensively studied autophagy inhibitors. These lysosomotropic agents block autophagy by preventing lysosomal acidification, thereby impeding autophagic vesicle degradation^33,37^. HCQ is favored clinically for several reasons: (i) its long-standing safety record in treating malaria and autoimmune diseases like lupus and rheumatoid arthritis^38,39^: (ii) high clinical tolerability with fewer severe side effects compared to other inhibitors^40^; (iii) oral administration, enabling convenient long-term use and combination with other cancer therapies^41^ and (iv) efficacy in blocking autophagic flux at the lysosomal level^40,42–45^.

Based on these findings, we aimed to evaluate the safety and tolerability (phase I) of adding HCQ to continuous low-dose (on-target) palbociclib and letrozole in metastatic HR+, HER2− breast cancer through a dose-escalation phase 1 study to determine the recommended phase 2 dose (RP2D), the dose-limiting toxicities (DLT) and efficacy of this combination.

## Methods

### Patient selection

Eligible patients were age ≥18 years with a histologically confirmed diagnosis of HR+/HER2− MBC by ASCO/CAP criteria^46,47^ and were candidates for treatment with CDK4/6 inhibitor (CDK4/6i) and ET with an aromatase inhibitor (AI) as standard of care. Patients had to have adequate renal (serum creatinine concentration <1.5 × ULN), hepatic (bilirubin level <1.5 × ULN. Aspartate aminotransferase (AST) or alanine aminotransferase (ALT) < 3 × ULN or alkaline phosphatase ≤2.5 ULN), and hematologic (ANC ≥ 1500 cells/μl; platelet count ≥100,000/μl) function; with an Eastern Cooperative Oncology Group (ECOG) performance status score of 0 or 1. Female patients had to be postmenopausal-defined as age ≥ 55 years and 1 year or more of amenorrhea; age <55 years and 1 year or more of amenorrhea with luteinizing hormone (LH) and/or follicle-stimulating hormone (FSH) levels in the postmenopausal range; age <55 with prior hysterectomy but intact ovaries with LH and/or FSH levels in the postmenopausal range; chemotherapy or medically induced ovarian suppression with 1 year or more of amenorrhea and with LH and/or FSH levels in the postmenopausal range; status after bilateral oophorectomy (≥28 days prior to first study treatment). Premenopausal patients were allowed on a later protocol amendment if receiving a gonadotropin-releasing hormone agonist.

The exclusion criteria were defined as follows: patients with prior exposure to CDK4/6 inhibitor therapy; a history of retinal disease or active visual disturbances (a normal baseline retinal examination, as specified by the study, was required); acute illnesses, including infections requiring medical therapy, known bleeding disorders, or the need for anticoagulation; and recent treatment with any of the following within 4 weeks prior to initiating study treatment: oral estrogens, including hormone replacement therapy; investigational agents (within 4 weeks or 5 half-lives of the agent, whichever was longer); or medications requiring concurrent use of strong CYP3A inhibitors or inducers. Additional exclusion criteria included psychological, familial, sociological, or geographical factors that could impede compliance with the study protocol; a life expectancy of less than 6 months; and pregnancy, lactation, or plans to become pregnant.

### Treatment plan

This is a single-center, open-label, non-randomized, standard 3 + 3 dose-escalation design^48^ phase I study (NCT03774472 registered in ClinicalTrials.gov on 8/20/2018) with the primary objective to evaluate the safety and tolerability of combining HCQ with continuous low-dose palbociclib (75 mg/day) and letrozole in patients with HR+/HER2− MBC and to establish the RP2D of HCQ (see Results section for the study design). The decision to use continuous low-dose palbociclib (75 mg/day) in this trial was based on emerging evidence from neoadjuvant studies showing spikes in cell proliferation during the 7 days off therapy in the standard 125 mg/day dosing schedule (administered 21 days on, 7 days off). These proliferation spikes, reflected by increases in both the proliferative index and serum thymidine kinase levels, suggest that the off-treatment interval may allow tumor regrowth, potentially reducing therapeutic efficacy^49–51^. To address this concern, we hypothesized that continuous dosing of palbociclib, when combined with HCQ and letrozole, could sustain therapeutic pressure and prevent these regrowth periods. At the time of this trial, preliminary data from other ongoing studies at Pfizer testing continuous palbociclib dosing at 75 mg/day supported the feasibility and tolerability of this regimen, providing a basis for its inclusion in our study design. This dosing strategy enabled the evaluation of continuous cell cycle inhibition as a means to optimize the therapeutic potential of the drug combination while maintaining safety and tolerability.

The secondary objective was to determine the response rate and clinical benefit rate at 8 weeks of the assigned HCQ dose plus continuous low-dose palbociclib and letrozole. For the Phase I portion of this study, tumor biopsies were optional, as the primary objective was to assess safety and establish the RP2D of HCQ. Tissue acquisition and analysis, including the use of archival specimens, were exploratory aims of this phase. As a result, not all patients provided tissue samples, and pre- and on-treatment biopsies were not universally collected. Only one patient had paired pre- and on-treatment biopsies available which were analyzed. The results presented here include IHC analysis for Ki67, Rb, and nuclear cyclin E for the pre-treatment samples from patients 2, 3, 4, 6, 8, 9, 10, 12, 13, and 15. We also are providing pre and on-treatment IHC results for Ki67, Rb, cyclin E, and 2 autophagy markers, p62, and LAMP1, for patient 3.

The study was approved by MD Anderson Cancer Center’s (Houston, TX) Institutional Review Board and was conducted in accordance with the declaration of Helsinki and Good Clinical Practice principles^52^. All patients provided written informed consent before starting any study-related procedures. The IRB protocol (MDACC 2017-0071) is included in the Supplementary material.

The study design schema is shown in the results section. A cycle was defined as 28 days, with DLT assessed over the first 28-day cycle. DLT is defined as grade 3 or 4 attributed to the study drug and excepting uncomplicated neutropenia ≤7 days (for a more detailed definition, refer to the protocol in the supplementary material). The dose escalation followed a standard 3 + 3 design^48^ to evaluate escalating doses of HCQ in combination with palbociclib (75 mg/kg taken orally once daily) and letrozole (2.5 mg/day taken orally once daily). At each dose level, three patients were initially enrolled and monitored for DLTs over one treatment cycle (28 days). If no DLTs were observed, the dose was escalated to the next cohort. If one patient experienced a DLT, the cohort was expanded to include an additional three patients. Escalation ceased if two or more patients in a cohort experienced DLTs, with the prior dose defined as the maximum tolerated dose. To define an RP2D, at least six evaluable patients were required at a given dose level.

For this trial, 14 patients were enrolled to determine the RP2D. Two patients (Study IDs 1 and 8) were excluded from the DLT assessment due to protocol-specified reasons: non-compliance during treatment (Study ID 1) and early disease progression before completing the DLT period (Study ID 8). These patients were replaced, ensuring 12 evaluable patients were included in the analysis. The RP2D was determined based on the safety and tolerability data from 3 patients treated at 400 mg HCQ, 3 at 600 mg HCQ, and 6 at 800 mg HCQ daily. Full details of patient outcomes and DLT assessments are provided in Table S2.

The primary objective of this trial phase was to assess safety and establish the RP2D of HCQ in combination with palbociclib and letrozole. Since the focus was on toxicity management rather than treatment response, measurable disease was not required for enrollment. Continued treatment beyond 8 weeks was reserved for patients demonstrating clear benefits, particularly those with partial responses (PR) as defined by RECIST criteria (≥30% reduction in the sum of the longest diameters of target lesions). Patients with stable disease (SD), defined as <30% decrease or less than a 20% increase in tumor size from baseline, were not treated beyond the DLT assessment phase. This approach prioritized safety while limiting exposure to potentially toxic therapy in patients without significant responses, aligning with the trial’s objective of defining a safe dosing strategy (IRB protocol included in the Supplementary Material).

### Efficacy and safety assessments

For the first 2 cycles (8 weeks), all patients were monitored with weekly office visits for physical exams and blood tests including complete blood count and leukocyte differential, electrolytes, blood urea nitrogen, creatinine, glucose, albumin, AST, and alanine aminotransferase, total bilirubin, alkaline phosphatase at baseline; thereafter patients were seen every 4 weeks. At baseline, urine or serum pregnancy test and urinalysis with microscopic exam were also performed. Additionally, a baseline HCQ screening ophthalmological exam per the latest guidelines of the American Academy of Ophthalmology (AAO) was performed on all patients, with repeat assessment required for new or worsening eye symptoms.

AEs were evaluated systematically to ensure comprehensive safety assessment and accurate attribution to the study drugs. A detailed breakdown of drug-associated AEs is provided in Table S1, where each AE is classified based on its potential relationship to the study drugs. Attribution of AEs was determined by the Principal Investigator (PI) or a designated co-investigator using a conservative approach, recognizing the potential for overlapping toxicities due to combination therapy. Consequently, some AEs were attributed to multiple drugs when definitive causation could not be established.

All AEs were assessed using the CTCAE Version 4.03 criteria, which included classification by type of adverse effect, severity grading (from mild, Grade 1, to severe, Grade 4), and attribution to one or more study drugs as suspected or unrelated. The duration of each AE was documented, noting the start and end dates or if the AE was ongoing at the time of the final examination. Actions taken in response to AEs, such as dose adjustment, temporary interruption, permanent discontinuation, or administration of concomitant medications, were also recorded. For serious adverse events (SAEs), criteria for severity and impact were evaluated to determine if they met SAE designation. This structured approach ensured thorough monitoring and documentation of AEs, facilitating a detailed understanding of the safety profile of the study drugs, both individually and in combination.

DLTs were defined as any death not clearly due to the underlying disease or extraneous causes; any grade 3 or higher non-hematologic toxicity; liver function abnormalities meeting Hy’s law^53^; neutropenic fever; grade 4 neutropenia or thrombocytopenia >7 days; grade 3 or higher thrombocytopenia with bleeding; grade 3 or higher nausea/vomiting or diarrhea ≥72 hours with adequate antiemetic and other supportive care; grade 3 or higher fatigue ≥1 week; grade 3 or higher electrolyte abnormality lasting >72 hours or of any duration with clinical symptoms.

Tumor response and progression were evaluated using Response Evaluation Criteria in Solid Tumors (RECIST) (version 1.1)^54^ once every 8 weeks or as clinically indicated. At week 8, if tumor response was observed, patients were allowed to continue treatment until progression or significant toxicities up to a maximum of 1 year. This timeframe was due to concerns about HCQ toxicities that could be treatment cumulative and would be tested in a follow-up controlled longer-term trial. Patients who showed progression or stability of disease at week 8 came off the study and continued standard treatment (standard dose palbociclib and letrozole) or alternate therapy at the discretion of the treating physician.

### Correlative biomarker assays

Sections of formalin-fixed, paraffin-embedded blocks (5 microns) of patient primary or metastatic tumor tissue were de-paraffinized (30 minutes at 72 °C) and rehydrated with antigen retrieval at 100 °C for 30 minutes with citrate buffer, pH 6.0. Endogenous peroxidase was blocked with 3% peroxide for 5 minutes. Slides from each patient were stained for antibodies against cyclin E (C-19, sc-198; Santa Cruz Biotechnology), Ki67 (#ab16667, 1:500 dilution; Abcam), and Rb (clone G3-245, BD Pharmagen^TM^, RRID:AB_385259) as described^55,56^. For patient 3, where pre and on-treatment slides were available, in addition to the three aforementioned antibodies, the slides were stained with two autophagy markers LAMP1 [Santa Cruz #sc-20011 RRID:AB_626853 (1:400)] and for p62 [Cell Signaling Technology #88588S, RRID:AB_2800125 (1:800)]. Post-primary antibody detection was conducted using a commercial polymer system (Bond Polymer Refine Detection, Leica), and stain development was achieved by incubating cells with DAB and DAB Enhancer (Leica). A positive batch of control slides was added for every immunohistochemistry run and on-control to each slide. Stained slides were scanned using the Aperio CS2 slide scanner (20X magnification) and subsequently analyzed for staining intensity using the positive pixel count tool in the Leica ImageScope software program (version 12.3), which measures both the area and intensity of positive staining per square millimeter of tissue. Percent staining positivity was defined as the number of positive cells per square millimeter of tissue analyzed for each sample.

### Statistical analysis

Descriptive summary statistics were used to characterize demographics, safety, and antitumor activity. AEs were identified for each drug and at each dose level for HCQ and reported by type and grade. Categorical data were summarized using frequencies, percentages, and 95% exact confidence intervals (CIs). Differences in categorical variables were assessed by Fisher’s exact tests. Continuous data were summarized by medians with 95% CIs and ranges.

## Results

### Patient characteristics

**Fig. 1:**
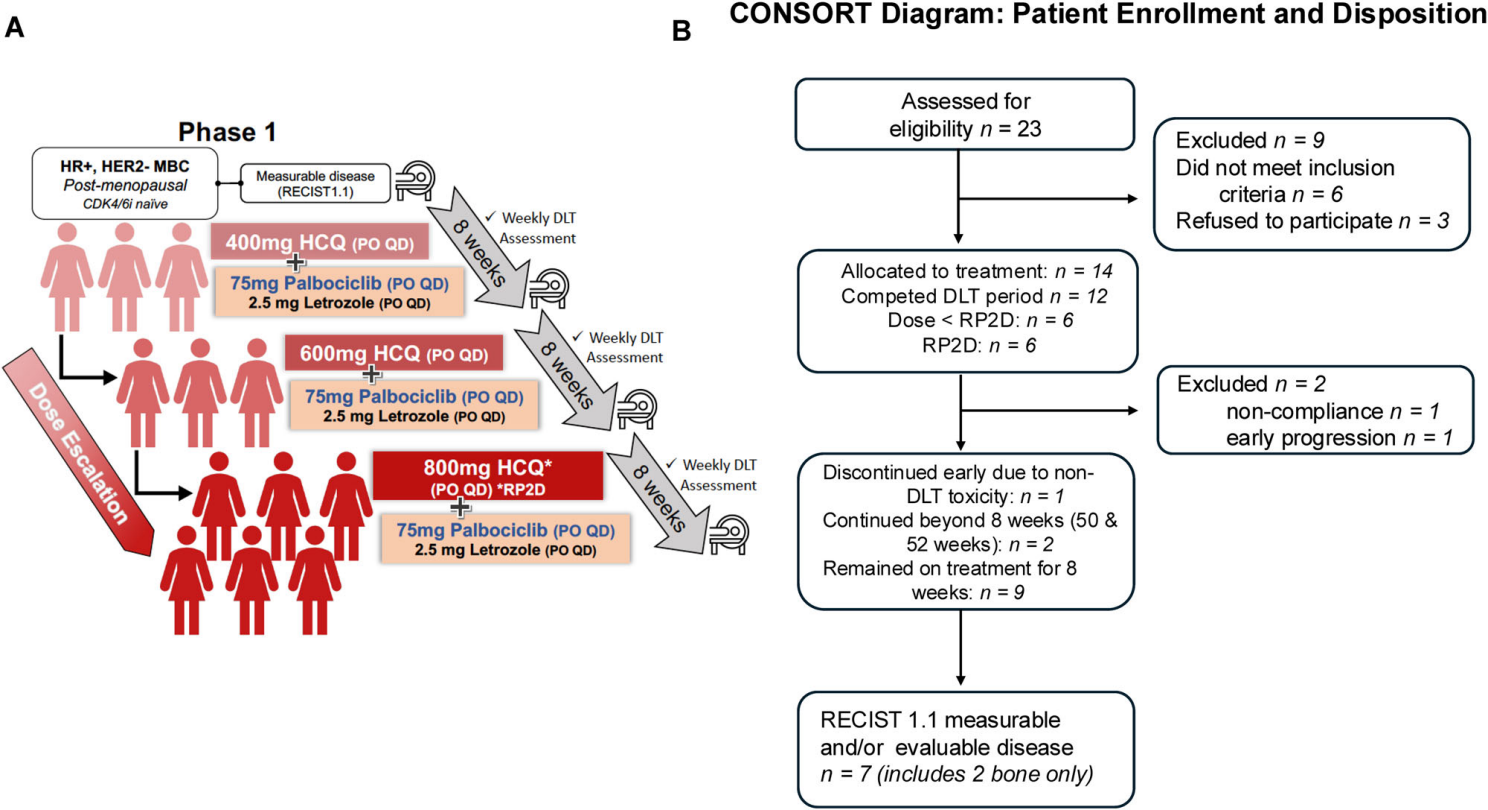
Study Design and Participant Flow in a Phase I Trial of Hydroxychloroquine, Palbociclib, and Letrozole in HR+/HER2- Metastatic Breast Cancer. **A** Study schema for dose escalation and dose expansion of hydroxychloroquine (HCQ) (400 mg, 600 mg, and 800 mg/day) in combination with fixed-dose continuous palbociclib (75 mg/day) and letrozole (2.5 mg/day). **B** Flow chart (CONSORT diagram) of study participants (enrollment and disposition).

Between September 2018 and December 2020, 23 patients signed informed consent for study enrollment at the MD Anderson Cancer Center as depicted in the study design schema (Fig. 1A); of these 6 patients did not meet secondary requirements and were considered “screen failures”, and 3 registered patients withdrew consent prior to receiving any treatment on the study (Fig. 1B). Two patients with measurable disease and documented response continued the study beyond 8 weeks (50 and 52 weeks) per protocol (Fig. 1B). The baseline clinical characteristics of these 14 patients are shown in Table 1. All were female and non-Hispanic, and their median age was 41 years (range 39–69), with the majority being Caucasian (79%).

**Table 1:** Patient characteristics

Characteristic	No. of patients (%) *N* = 14
Median age years	41 (range 39–69)
Sex	
Female	14 (100)
Race	
White	11 (79)
Black or African American	2 (14)
Asian	1 (7)
Menopausal status	
Pre	3 (21)
Peri	1 (7)
Post	10 (72)
ECOG performance status score	
0	11 (79)
1	3 (21)
Site of Metastasis	
Visceral	5 (36)
Non- visceral	9 (64)
Prior chemotherapy before metastasis	
Yes	10 (71)
No	4 (29)
Prior endocrine therapy for metastatic disease	
Yes	3 (21)
No	11 (79)
Prior radiation therapy	
Yes	10 (71)
No	4 (29)
No. of prior therapies for metastatic disease (range 0–6)	
0	12 (86)
3	1 (7)
6	1 (7)

All data are no. of patients (%) unless otherwise indicated.

*CDK4/6i* CDK4/6 inhibitors, *ECOG* Eastern Cooperative Oncology.

None of the 14 patients on the trial received CDK4/6 inhibitor (CDK4/6i) therapy prior to enrollment, as this was explicitly excluded per the study protocol. Twelve patients (Study IDs 1, 2, 3, 5, 8, 9, 10, 11, 12, 13, 14, and 15) were diagnosed with metastatic disease at the time of accrual (between September 2018 and December 2020) and entered the trial immediately following their diagnosis, without exposure to any prior CDK4/6i therapy. Two patients who had been diagnosed with metastatic disease prior to the accrual period had received multiple lines of therapy for metastatic disease, but none of these treatments included CDK4/6i. For patient Study ID 4, prior treatments included Taxol (chemotherapy) in 2013, fulvestrant, and the combination of everolimus and exemestane (endocrine therapies) in 2014, followed by Xeloda (chemotherapy) until 2018. This patient subsequently enrolled in a Phase 1 trial involving a STING agonist and PDR001 in 2018, progressed on that therapy, and was then enrolled in our clinical trial without prior CDK4/6i exposure. Similarly, patient Study ID 7 received fulvestrant and the combination of everolimus and exemestane (endocrine therapies) until 2016, followed by Xeloda (chemotherapy) until 2019, after which she enrolled in our trial. Like the other patients, this individual had no prior exposure to CDK4/6is before participating in the study. This ensures that all enrolled patients met the exclusion criterion regarding prior CDK4/6i treatment. In summary, out of 14 patients identified, 12 patients received study treatment as 1st line and 2 patients as 4th line and 7th line.

Most patients underwent additional therapy after coming off the study, which included CDK4/6i + ET, chemotherapy/targeted therapies, or enrollment in other clinical trials. Table 2 summarizes the sequence and frequency of subsequent therapies received by 14 patients after discontinuation of the trial.

**Table 2:** Subsequent therapy following the Phase I trial

Subsequent therapy after trial	No. of patients (%) *N* = 14
Post palbociclib + HCQ 1st therapy	
CDK4/6i + ET	11 (79)
Clinical trial	1 (7)
Chemotherapy	2 (14)
Post palbociclib + HCQ 2nd (immediately off study) therapy	
CDK4/6i + ET	2 (14)
Chemotherapy	3 (21)
Targeted therapy	2 (14)
Endocrine therapy	1 (7)
Clinical trial	1 (7)
No further treatment	5 (36)
Post palbociclib + HCQ 3rd therapy	
CDK4/6i + ET	1 (7)
Chemotherapy	1 (7)
Clinical trial	1 (7)
Targeted therapy	2 (14)
No further treatment	9 (65)
Post palbociclib + HCQ 4th therapy	
CDK4/6i + ET	2 (14)
Chemotherapy	1 (7)
Antibody-drug conjugate	1 (7)
No further treatment	10 (72)
Post palbociclib + HCQ 5th therapy	
Antibody-drug conjugate	1 (7)
No further treatment	13 (93)
Post palbociclib + HCQ 6th therapy	
Clinical Trial	1 (7)
No further treatment	13 (93)
Post palbociclib + HCQ 7th therapy	
Clinical trial	1 (7)
No further treatment	13 (93)

All data are no. of patients (%) unless otherwise indicated.

*CDK4/6i*, CDK4/6 inhibitors, *ET* endocrine therapy

### Safety and dose escalation

**Fig. 2:**
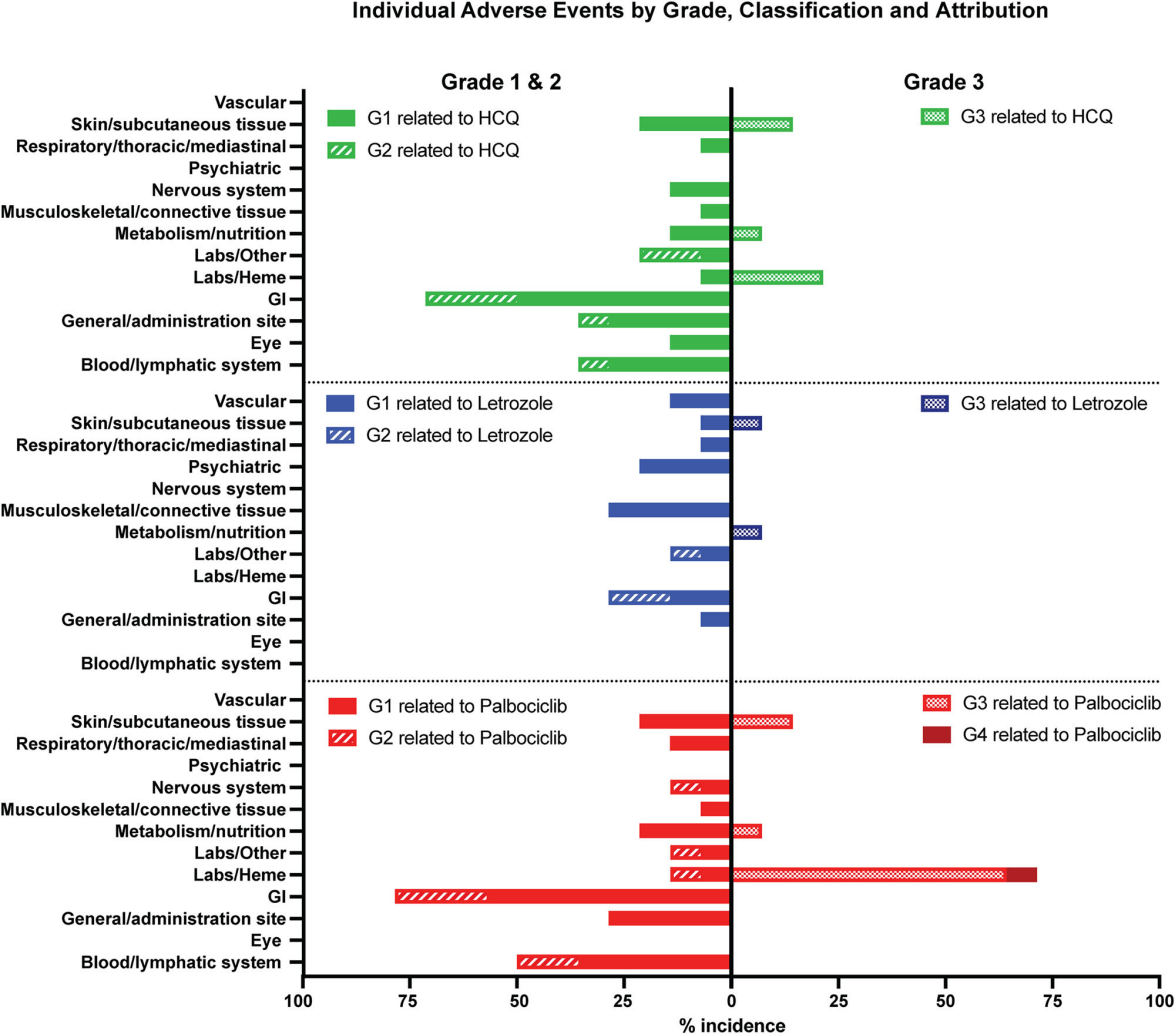
Summary of Treatment-Emergent Adverse Events by Severity for Each of the Three Study Drugs. Toxicity assessment board for frequencies and grades of unique treatment-emergent adverse events for HCQ (top panels), letrozole (middle panels and palbociclib (bottom panels); Grades 1 and 2 adverse events per each drug are depicted to the right, and grades 3 and 4 adverse events per each drug are depicted to the left.

**Fig. 3:**
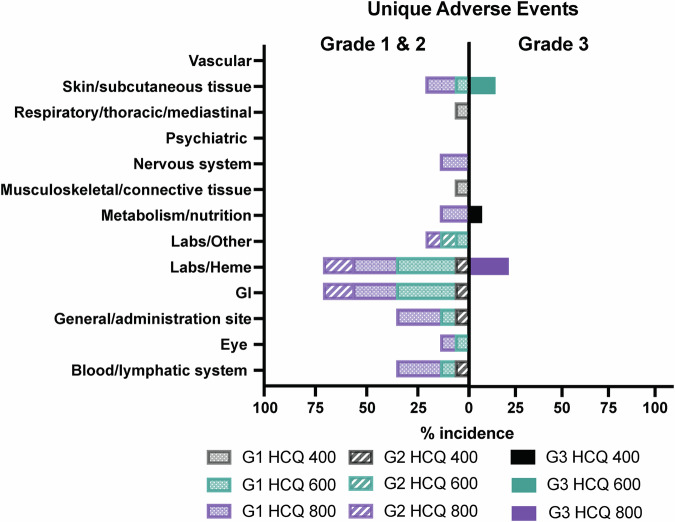
Adverse Event Profile of Hydroxychloroquine Across Escalating Dose Levels. Unique adverse events for frequencies and grades for each of the 3 doses of HCQ (400, 600, and 800 mg/day), with grades 1 and 2 events on the left and grade 3 on the right. No grade 4 adverse events for any dose of HCQ were observed in any of the patients.

All 14 patients who had received at least one dose of HCQ in any of the three cohorts, were evaluable for safety and efficacy (4 at 400 mg, 4 at 600 mg, and 6 at 800 mg). Of the 14 patients enrolled in the study, two were not evaluable for DLT (Fig. 1B). One patient in the first cohort discontinued the study at week 3 due to non-compliance, and one patient in the second cohort experienced early disease progression at week 4, just prior to completing the DLT period. As per protocol, these two patients were replaced, resulting in 12 patients being evaluated for DLT analysis across the three dose levels. There were no treatment-related deaths in the study. Treatment-related AEs according to grade and drug attribution (palbociclib, letrozole, or HCQ) are shown in Fig. 2 and Supplementary Table 1. Figure 3 depicts dose-specific AEs associated with HCQ. The majority of patients (79%) treated with any of the three doses of HCQ (400, 600, or 800 mg/day) experienced only grade 1 or 2 AEs. AEs of grade 3 associated with HCQ were hematological in 3 of the patients at the 800 mg dose level, skin rash in 2 of the patients at the 600 mg dose level, and anorexia in 1 of the patients at the 400 mg dose level and (Supplementary Table 1 and Fig. 3). HCQ treatment was not associated with any grade 4 AEs at any dose level in any of the patients. One patient discontinued early at 4 weeks shortly after the DLT period with a grade 3 rash that was attributed to HCQ (but worsened by sun exposure). Ocular toxicities were seen in 4 patients (29%) and were all grade 1. The only grade 3 AEs associated with letrozole were skin rash in 1 patient and anorexia in 1 patient. Letrozole treatment was not associated with any grade 4 AEs (Fig. 2 and Supplementary Tables 1 and 2). The AEs of grade ≥3 associated with palboclicib were hematologic in ten patients (71%), skin rash in two patients (14%), and anorexia in one patient (7%), with no serious AEs reported.

No DLTs were seen in the 1st cohort (400 mg/day) and hence the 2nd cohort (600 mg/day) and 3rd cohort (and final target dose) of 800 mg of HCQ per day in combination with continuous dosing palbociclib at 75 mg/day and letrozole at 2.5 mg/day were successively activated. No DLTs were observed in the overall study; hence, 800 mg was declared as the RP2D for HCQ.

There were no dose modifications or reductions of HCQ. Nine patients (64%) had at least one dose delay of palbociclib because of grade 3 neutropenia (Supplementary Table 2). No clear association was observed between HCQ dose level and the percent of total days of palbociclib dosing (ranging from 0 to 37.5%) that was delayed due to neutropenia (absolute neutrophil count <1000).

### Efficacy and biomarker assessments

**Fig. 4:**
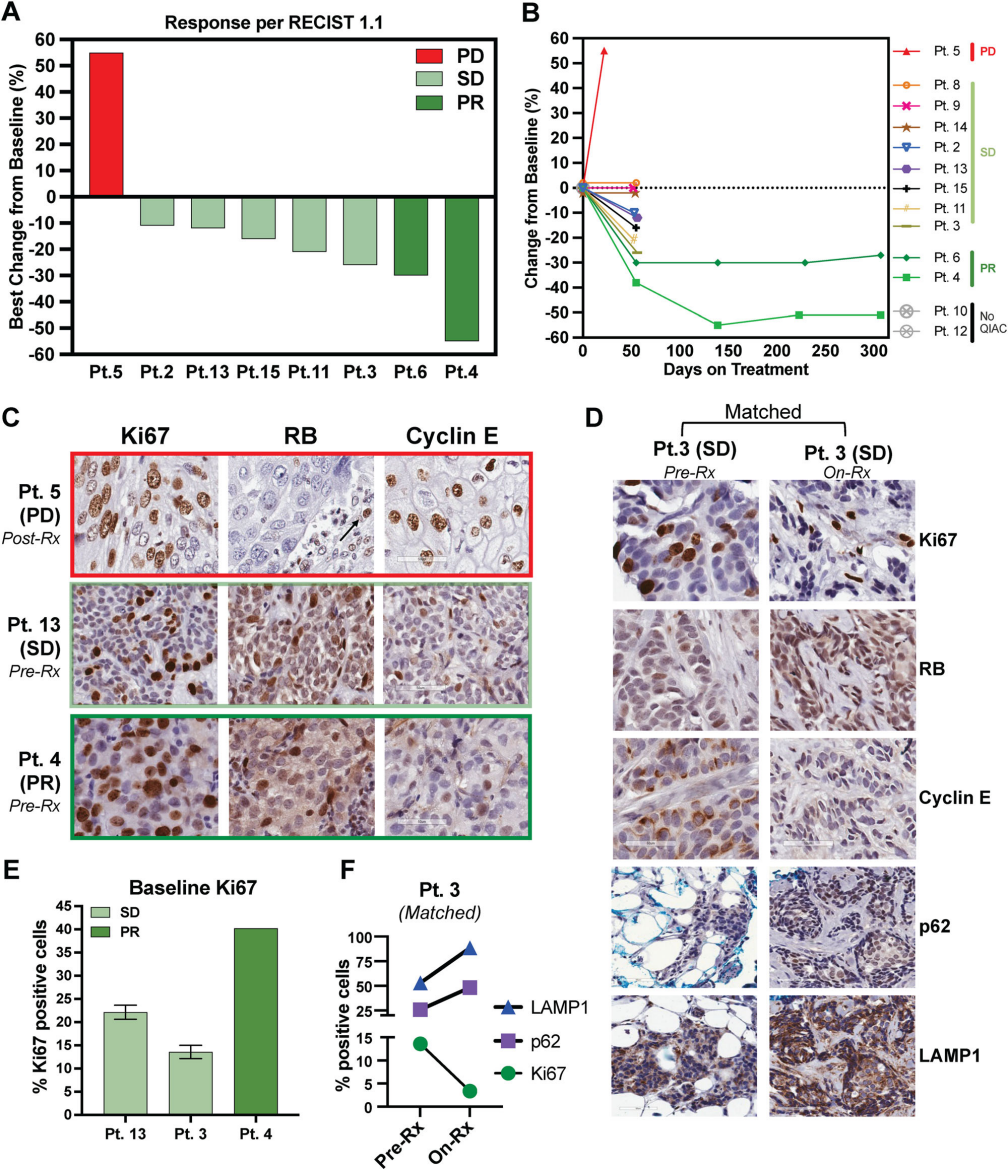
Tumor Response by RECIST Criteria and Biomarker Analysis of Ki67, Rb, Cyclin E, p62, and LAMP1 in Patients Treated with HCQ + Palbociclib + Letrozole. **A** Waterfall plot depicting the best percentage change in the sums of the diameters of target lesions per RECIST 1.1 in patients with measurable disease. **B** Tumor Response Over Time by RECIST Criteria in Patients on Treatment. percentage change from baseline in tumor size over time (days on treatment) for individual patients treated with a combination of HCQ, palbociclib, and letrozole. Each patient’s response is color-coded to indicate their response category according to RECIST criteria: progressive disease (PD), stable disease (SD), or partial response (PR). Patients 10 and 12 are labeled as “No QIAC,” indicating they were on treatment but lacked quantifiable imaging data to determine their exact response. **C** Immunohistochemistry (IHC) staining on biopsy from patient 5 (Pt. 5) with PD after treatment (post-Rx) indicates increased proliferation (Ki67), lack of RB expression in tumor (arrow indicates positive RB expression in stromal cells), and cyclin E levels. IHC for Ki67, RB, and cyclin E was performed on biopsies obtained from Pt. 4 with PR and Pt 13 SD prior to treatment (baseline). **D** Pt. 3 had two biopsies: baseline and on treatment (On-Rx), and each was stained with Ki67, Rb, and cyclin E as well as two autophagy markers, p62 and LAMP1. **E** Quantification of Ki67 in C at baseline for Pt 13, Pt 3, and Pt 4. **F** Quantification of Ki67, p62, and LAMP1 in matched biopsies from Pt.3. There is a 10% reduction in Ki67 and a 25% and 35%% increase in p62 and LAMP1, respectively, while on treatment from baseline. PD progressive disease, SD stable disease, PR partial response.

The median follow-up time for all patients was 47.1 months. All patients underwent restaging CT scans at 8 weeks to assess initial response according to RECIST 1.1. Patients with measurable disease and responses were eligible to continue treatment, undergoing scans every 8-12 weeks for up to 1 year, provided there was no progression or as decided by the patient and/or by the investigator-defined response. One patient had a progression of disease at 4 weeks, detected clinically as progression in an ipsilateral axillary node at 3 weeks, and was taken off the study. Of the 14 patients, PR was observed in two patients (14.3%), SD at 8 weeks was observed in 11 (78.6%), and clinical progression in one patient (7.1%). For measurable disease, tumor decreases by RECIST criteria of 11%, 12%, 21%, 26%, 30%, and 55%, and an increase in 1 patient by 55% progressive disease (PD) was observed (Fig. 4A). The percentage change in tumor size from baseline over time is depicted in a spider plot (Fig. 4B) for individual patients treated with HCQ, palbociclib, and letrozole, categorized according to RECIST criteria: PR, SD, or PD. One patient (Patient 5) exhibited PD with a rapid tumor size increase exceeding 50% within the first 50 days. Seven patients (Patients 3, 8, 9, 14, 2, 13, and 15) demonstrated SD, with tumor size changes fluctuating slightly but remaining within a stable range (<30% decrease or less than 20% increase). Two patients (Patients 6 and 4) achieved PRs, with tumor size reductions exceeding 30%. Notably, Patient 6 maintained a 55% reduction, the maximum observed, over ~300 days, suggesting a durable response. Two patients (Patients 10 and 12) lacked sufficient imaging data to quantify their response.

IHC analysis of Rb, cyclin E, and Ki67 was performed on available tumor biopsies (Fig. 4C and Supplementary Fig. 1). The results revealed that Rb was expressed in tumors from patients with SD and PR but was absent in the tumor cells of the one patient who experienced PD (patient 5, Fig. 4C). Notably, in patient 5, Rb expression was restricted to the stromal compartment, as indicated by the arrow in Fig. 4C. Nuclear cyclin E expression was detected in all patient tumors, indicating no evidence of cytoplasmic cyclin E-mediated intrinsic resistance to CDK4/6i. Ki67 expression in pre-treatment biopsies ranged from 13 to 37% in patients 13, 3, and 4. Importantly, Ki67 levels in patient 3 decreased to below 2.7% during treatment, consistent with the therapeutic activity of the regimen (Fig. 4D–F). Additionally, the autophagy markers p62 and LAMP1^33,57^ were evaluated in pre-treatment and on-treatment samples for patient 3. The analysis showed increased expression of both markers in the on-treatment sample compared to the pre-treatment sample (Fig. 4D, F), suggesting that the addition of HCQ to the treatment regimen effectively inhibited the autophagy pathway in this patient.

## Discussion

To our knowledge, this is the first prospective clinical trial reporting results on CDK4/6 inhibitor (CDK4/6i) and ET concurrently with an autophagy inhibitor, HCQ. The combination of low-dose palbociclib, letrozole, and HCQ, in which palbociclib was administered continuously at 75 mg/day, letrozole at 2.5 mg/day (FDA-approved dose) and HCQ at 3 dose levels (400 mg/day, 600 mg/day and 800 mg/day) demonstrated an acceptable safety profile and clinical feasibility in hormone receptor-positive and HER2− MBC. Safe escalation to 800 mg HCQ was accomplished with no DLTs. The RPD2 of HCQ was 800 mg with continuous palbociclib at 75 mg/d and letrozole 2.5 mg/d. There appears to be an accentuation of fatigue, gastrointestinal symptoms, dysgeusia, and rash with the addition of HCQ. Prominent neutropenia was seen, necessitating delays in palbociclib with this regimen compared to standard palbociclib at 125, 100, or 75 mg for 21 days on and 7 days off. It is not clear if this is due to continuous dosing palbociclib or the addition of HCQ. There was a trend toward more neutropenia at the highest HCQ level, or days of palbociclib held due to neutropenia (palbociclib held for absolute neutrophil count <1000). Additionally, the overall rate of 14.2% of days of delay of palbociclib may overestimate hematologic toxicity since most patients waited 7 or more days prior to rechecking blood counts.

Our preclinical study^29^ supported the continuous dosing of palbociclib used in this study, and a neoadjuvant trial testing palbociclib with anastrozole did, in fact, show that more patients exhibited a proliferative burst as assessed by the proliferative antigen Ki67 when stopping palbociclib with a longer break before surgery than a short or no break^49^. Efforts are ongoing to explore lower continuous dosing, with a schedule of 5 days on and two days off weekly at a palbociclib dose of 125 mg/d preliminarily showing this dose was feasible, with 36% of patients experiencing grade 3 neutropenia, less than the hypothesized rate^58^. Thus, the concept of autophagy inhibition with a lower dose of continuous palbociclib is expected to be feasible. As described earlier, these findings have implications for augmenting the effects of other approved CDK4/6is, mTOR, PI3K, and AKT inhibitors used with ET.

Responses reported after the initial 8-week period may not be representative of the long-term activity that would be assessed in a phase II trial, but our design to limit longer-term treatment was necessary until we better understand the toxicity profile such that patients could receive standard care for their disease. Nevertheless, the combination exhibited encouraging activity, eliciting two durable PRs and 11 patients with SD that requires confirmation with a Phase II trial. The overall response rate was 2 of 7 (29%) in those with measurable disease and 8-week disease control was seen in 11 of 12 evaluable patients (92%).

Responses were independent of prior exposure to ET, which suggests that triple combination can overcome mechanisms of resistance; however, the small numbers of patients treated in our study preclude any definite conclusion. In addition, two patients who had measurable and responsive disease remained on therapy for at least 50 weeks.

Our preclinical findings demonstrate that combining autophagy inhibitors, such as HCQ, with CDK4/6is induces irreversible growth arrest and establishes a stable state of cellular senescence in cancer cells^29^. This combination is particularly relevant in the adjuvant setting, where the primary goal is to achieve durable tumor suppression and prevent late recurrences^59^. While senescence is a critical tumor-suppressive mechanism, its associated secretory phenotype (SASP) can drive drug resistance and promote a pro-tumorigenic microenvironment over time^60,61^. Autophagy inhibition addresses this challenge by destabilizing the survival pathways of senescent cells, creating a “senescence lock” that prevents these cells from re-entering the cell cycle or escaping dormancy^62^. Additionally, autophagy inhibitors function as senolytics, selectively eliminating senescent cells and mitigating SASP-driven effects that could facilitate tumor recurrence^63,64^. By clearing these cells and preventing their escape from dormancy, this approach enhances the antitumor durability of CDK4/6is, reduces the residual disease burden, and fosters a tumor microenvironment less conducive to recurrence, thereby improving long-term outcomes in patients.

Although the study regimen is feasible, extended HCQ dosing may increase toxicities and potentially disrupt optimal long-term palbociclib administration. The combination demonstrated encouraging activity, such as PRs, prolonged SD (in patients without measurable disease), and promising overall survival benefits. However, newer autophagy inhibitors with fewer off-target toxicities may offer more favorable responses and improved tolerability. One such inhibitor is Lys05 is a potent next-generation autophagy inhibitor that outperforms HCQ^65,66^. It effectively accumulates in lysosomes and inhibits autophagy, making it ideal for combination cancer therapies. Lys05 enhances the impact of radiation on NSCLC cells^67^ and boosts the cytotoxicity of BRAF inhibitors in melanoma, particularly in resistant cells^68^. It also enhances palbociclib’s effect in breast cancer models at lower doses than HCQ^29^, potentially reducing toxicity in clinical trials. Additionally, ULK1/2 inhibitors, such as SBI-0206965^69^ and DCC-3116^70^, have shown effectiveness in blocking autophagy initiation, offering targeted approaches for cancer treatment. Combining ULK1/2 inhibitors with existing therapies can improve outcomes by selectively inhibiting autophagy pathways. Currently, DCC-3116 is undergoing Phase 1/2 clinical trials (NCT04892017, NCT05957367) to evaluate its safety, tolerability, and preliminary efficacy as both a monotherapy and in combination with other cancer therapies, such as MEK and KRAS G12C inhibitors, in patients with advanced or metastatic solid tumors.

Our study also had some limitations. We enrolled relatively few patients with diverse tumor characteristics. Dose escalation also limited the optimal interpretation of activity. Our study did not include analyses of additional factors, which could have impacted outcomes, such as autophagy biomarkers pre and post-treatment samples or pharmacokinetics assessments. Despite these limitations, our study demonstrated that the combination of palbociclib, letrozole, and HCQ has a favorable safety profile. Recognizing the limitations of the exploratory tissue analysis in this Phase I study, we have designed a future randomized Phase II “window” trial in the early-stage setting to address these gaps. This trial will evaluate the feasibility and efficacy of autophagy inhibition with HCQ in combination with low-dose continuous CDK4/6i to reverse resistance or enhance therapeutic activity. Conducted in the neoadjuvant setting, this study will include mandatory pre- and post-treatment biopsies, allowing for a brief (2-week) exposure period to thoroughly investigate biomarkers and pharmacodynamic changes. Particular focus will be given to biomarkers strongly associated with autophagy modulation, providing critical insights into their potential role in predicting clinical outcomes and guiding therapeutic strategies. Additional studies using more targeted autophagy inhibitors in development may provide a better benefit/toxicity ratio needed for longer-term testing if this concept is proven in the phase II setting.

## Supplementary information

Supplementary Figure 1, Supplementary Table 1 and Supplementary Table 2

IRB protocol for the Palbociclib + HCQ clinical trial

## Data Availability

The data that support the findings of this study are available from the corresponding author, upon reasonable request.
