# Supplementary material for: Phase I trial of hydroxychloroquine to enhance palbociclib and letrozole efficacy in ER+/HER2− breast cancer
Source: NPJ Breast Cancer. 2025 Jan 26;11:7. doi: 10.1038/s41523-025-00722-1 (PMC11770068; doi:10.1038/s41523-025-00722-1)
Supplement: Supplementary file 1 — Supplementary Figure 1, Supplementary Table 1 and Supplementary Table 2 [file 41523_2025_722_MOESM1_ESM.pdf]

Phase I Trial of Hydroxychloroquine to Enhance Palbociclib and Letrozole Efficacy in ER+/HER2- Breast Cancer

Akshara Singareeka Raghavendra, Nicole M. Kettner, Danielle Kwiatkowski, Senthil Damodaran, Yan Wang, David Ramirez, Dan S. Gombos, Kelly K. Hunt, Yu Shen, Khandan Keyomarsi, Debu Tripathy

Supplementary Figure 1 and Supplementary Table 1 and Supplementary Table 2

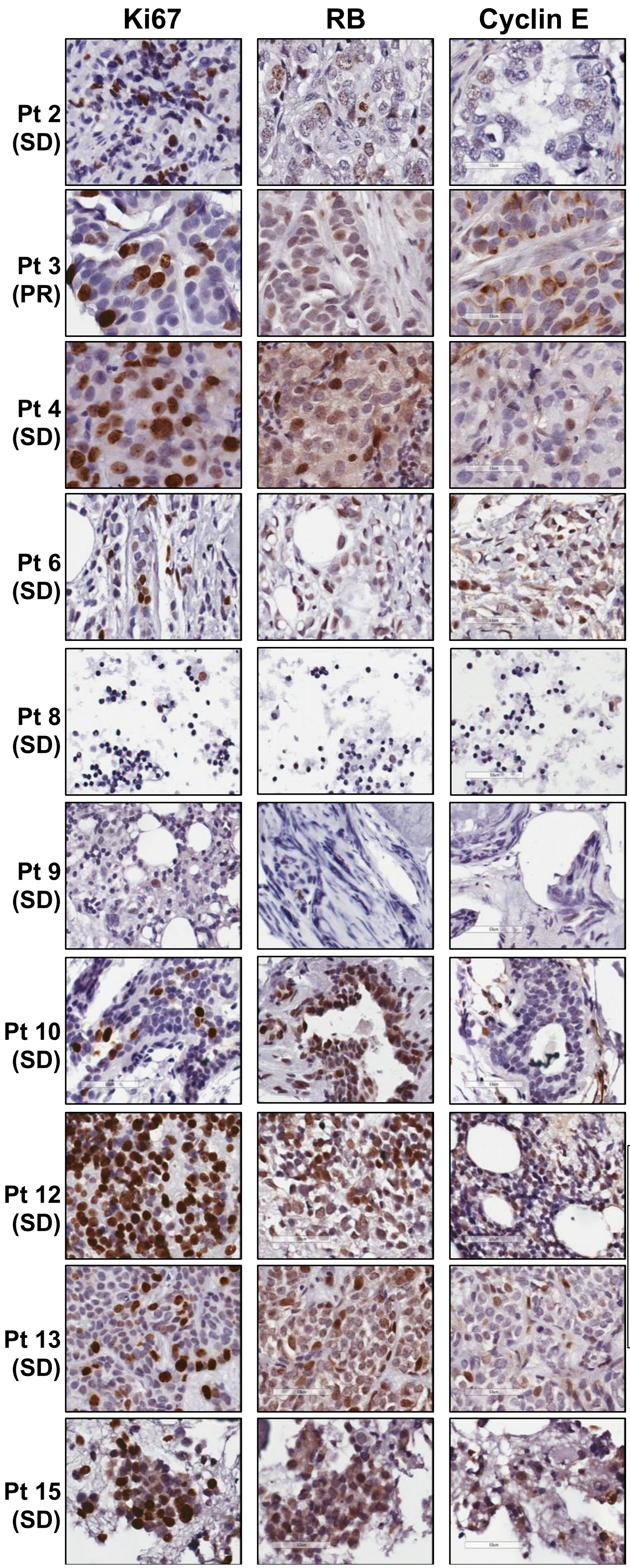

Supplementary Figure 1: IHC staining of Ki67, Rb and cyclin E of pre-treatment biopsies from all patients (except patients 11 and 14 for whom slides were not available).

Supplementary Table 1: Adverse events occuring in all patients by grade per treatment

Classification	AE term (subcategory)	Grade	Palbocicclib, No (%)	Letorzole, No (%)	HCQ 400mg/day, No (%)	HCQ 600mg/day, No (%)	HCQ 800mg/day, No (%)	HCQ all doses, No (%)
		G1	5 (35.7)	0 (0)	0 (0)	1 (7.1)	3 (21.4)	4 (28.6)
Blood/lymphatic system	Anemia	G2	2 (14.3)	0 (0)	1 (7.1)	0 (0)	0 (0)	1 (7.1)
		G3	0 (0)	0 (0)	0 (0)	0 (0)	0 (0)	0 (0)
		G4	0 (0)	0 (0)	0 (0)	0 (0)	0 (0)	0 (0)
		G1-4	7 (50)	0 (0)	1 (7.1)	1 (7.1)	3 (21.4)	5 (35.7)
Eye	Blurred vision	G1	0 (0)	0 (0)	0 (0)	1 (7.1)	0 (0)	1 (7.1)
		G2	0 (0)	0 (0)	0 (0)	0 (0)	0 (0)	0 (0)
		G3	0 (0)	0 (0)	0 (0)	0 (0)	0 (0)	0 (0)
		G4	0 (0)	0 (0)	0 (0)	0 (0)	0 (0)	0 (0)
		G1-4	0 (0)	0 (0)	0 (0)	1 (7.1)		1 (7.1)
Eye	Eye disorders - Other	G1	0 (0)	0 (0)	0 (0)	0 (0)	1 (7.1)	1 (7.1)
		G2	0 (0)	0 (0)	0 (0)	0 (0)	0 (0)	0 (0)
		G3	0 (0)	0 (0)	0 (0)	0 (0)	0 (0)	0 (0)
		G4	0 (0)	0 (0)	0 (0)	0 (0)	0 (0)	0 (0)
		G1-4	0 (0)	0 (0)	0 (0)	0 (0)	1 (7.1)	1 (7.1)
GI	Nausea/Diarrhea	G1	8 (57.1)	2 (14.3)	1 (7.1)	4 (28.6)	5 (35.7)	10 (71.4)
		G2	3 (21.4)	2 (14.3)	1 (7.1)	0 (0)	2 (14.3)	3 (21.4)
		G3	0 (0)	0 (0)	0 (0)	0 (0)	0 (0)	0 (0)
		G4	0 (0)	0 (0)	0 (0)	0 (0)	0 (0)	0 (0)
Investigations/Heme	Neutrophil count decreased	G1	1 (7.1)	0 (0)	1 (7.1)	4 (28.6)	5 (35.7)	10 (71.4)
		G2	1 (7.1)	0 (0)	1 (7.1)	0 (0)	2 (14.3)	4 (28.6)
		G3	9 (64.3)	0 (0)	0 (0)	0 (0)	3 (21.4)	3 (21.4)
		G4	1 (7.1)	0 (0)	0 (0)	0 (0)	0 (0)	0 (0)
Investigations/Other	Creatinine/ALP increased	G1	1 (7.1)	1 (7.1)	0 (0)	1 (7.1)	0 (0)	1 (7.1)
		G2	1 (7.1)	1 (7.1)	0 (0)	1 (7.1)	1 (7.1)	2 (14.3)
		G3	0 (0)	0 (0)	0 (0)	0 (0)	0 (0)	0 (0)
		G4	0 (0)	0 (0)	0 (0)	0 (0)	0 (0)	0 (0)
Metabolism/nutrition	Anorexia	G1	3 (21.4)	0 (0)	0 (0)	0 (0)	2 (14.3)	2 (14.3)
		G2	0 (0)	0 (0)	0 (0)	0 (0)	0 (0)	0 (0)
		G3	1 (7.1)	1 (7.1)	1 (7.1)	0 (0)	0 (0)	1 (7.1)
		G4	0 (0)	0 (0)	0 (0)	0 (0)	0 (0)	0 (0)
Musculoskeletal/	Weakness/Arthralgia/	G1	1 (7.1)	4 (28.6)	1 (7.1)	0 (0)	0 (0)	1 (7.1)
connective tissue	Myalgia/other	G2	0 (0)	0 (0)	0 (0)	0 (0)	0 (0)	0 (0)
		G3	0 (0)	0 (0)	0 (0)	0 (0)	0 (0)	0 (0)
		G4	0 (0)	0 (0)	0 (0)	0 (0)	0 (0)	0 (0)
Nervous system	Dysgeusia/Dizziness/	G1	1 (7.1)	0 (0)	0 (0)	0 (0)	2 (14.3)	2 (14.3)
	Headache	G2	1 (7.1)	0 (0)	0 (0)	0 (0)	2 (14.3)	2 (14.3)
		G3	0 (0)	0 (0)	0 (0)	0 (0)	0 (0)	0 (0)
		G4	0 (0)	0 (0)	0 (0)	0 (0)	0 (0)	0 (0)
Psychiatric	Insomnia	G1	0 (0)	3 (21.4)	0 (0)	0 (0)	0 (0)	0 (0)
		G2	0 (0)	0 (0)	0 (0)	0 (0)	0 (0)	0 (0)
		G3	0 (0)	0 (0)	0 (0)	0 (0)	0 (0)	0 (0)
		G4	0 (0)	0 (0)	0 (0)	0 (0)	0 (0)	0 (0)
Respiratory/thoracic/	Epistaxis	G1	2 (14.3)	1 (7.1)	1 (7.1)	0 (0)	0 (0)	1 (7.1)
mediastinal		G2	0 (0)	0 (0)	0 (0)	0 (0)	0 (0)	0 (0)
		G3	0 (0)	0 (0)	0 (0)	0 (0)	0 (0)	0 (0)
		G4	0 (0)	0 (0)	0 (0)	0 (0)	0 (0)	0 (0)
Skin/subcutaneous tissue	Alopecia/Rash	G1	3 (21.4)	1 (7.1)	0 (0)	1 (7.1)	2 (14.3)	3 (21.4)
		G2	0 (0)	0 (0)	0 (0)	0 (0)	0 (0)	0 (0)
		G3	2 (14.3)	1 (7.1)	0 (0)	2 (14.3)	0 (0)	2 (14.3)
		G4	0 (0)	0 (0)	0 (0)	0 (0)	0 (0)	0 (0)
Vascular	Hot flashes	G1	0 (0)	2 (14.3)	0 (0)	0 (0)	0 (0)	0 (0)
		G2	0 (0)	0 (0)	0 (0)	0 (0)	0 (0)	0 (0)
		G3	0 (0)	0 (0)	0 (0)	0 (0)	0 (0)	0 (0)
		G4	0 (0)	0 (0)	0 (0)	0 (0)	0 (0)	0 (0)

Supplementary Table 2. Days of Palbociclib Held Due to Neutropenia (% of total time)

Study ID	Dose Level HCQ (mg)	Weeks on Study	Total # days palbociclib held due to neutropenia	
1	400	3	0 (0%)	
2	400	8	7 (12.5%)	
3	400	8	14 (25%)	7.4%
4	400	50	44 (12.5%)	
5	600	4	0 (0%)	
6	600	52	7 (1.9%)	3%
7	600	8	7 (12.5%)	
8	600	4	0 (0%)	
9	800	8	0 (0%)	
10	800	8	0 (0%)	
11	800	8	14 (25%)	17%
12	800	8	7 (12.5%)	
13	800	8	14 (25%)	
14	800	8	21 (37.5)	
Total All Patients		185	135 (10.4%)	

Note: Twelve of fourteen patients were on study for 8 weeks and stopped per protocol, unless they had measurable disease (Study ID 5) and objective response by RECIST 1.1 criteria per protocol to stay on beyond 8 weeks (Study ID 4 and 6). Two patients (Study ID 1 and 8) were removed from DLT assessment and dose escalation due to non-compliance at weeks 3 in cohort 1 and due to early progression of disease at week 4 in cohort 2, just prior to the end of DLT period. These 2 patients were replaced as per protocol, and the remaining 12 patients were enrolled to three dose levels (3 to 400mg HCQ, 3 to 600mg HCQ and 6 to the 800mg HCQ cohorts).
