# Supplementary material for: Phase I trial of hydroxychloroquine to enhance palbociclib and letrozole efficacy in ER+/HER2− breast cancer
Source: NPJ Breast Cancer. 2025 Jan 26;11:7. doi: 10.1038/s41523-025-00722-1 (PMC11770068; doi:10.1038/s41523-025-00722-1)
Supplement: Supplementary file 2 — IRB protocol for the Palbociclib + HCQ clinical trial [file 41523_2025_722_MOESM2_ESM.docx]

| **MD Anderson IND Sponsor Cover Sheet** | |
| --- | --- |
|  | |
| **Protocol ID** | 2017-0071 |
| **Protocol Title** | Phase I/II Safety and Efficacy Study of Autophagy Inhibition with Hydroxychloroquine to Augment the Antiproliferative and Biological Effects of Pre-Operative Palbociclib plus Letrozole for Estrogen Receptor-Positive and HER2-negative Breast Cancer |
| **Protocol Version** | 07 |
| **Version Date** | 03/17/2021 |
|  | |
| **Protocol PI** | Debu Tripathy, MD |
| **Department** | Breast Medical Oncology |
|  | |
| **IND Sponsor** | MD Anderson Cancer Center |
| **IND #** | 138453 |

Phase I/II Safety and Efficacy Study of Autophagy Inhibition with Hydroxychloroquine to Augment the Antiproliferative and Biological Effects of Pre-Operative Palbociclib plus Letrozole for Estrogen Receptor-Positive and HER2-negative Breast Cancer

Principal Investigator: Debu Tripathy, MD

Professor and Chairman, Breast Medical Oncology

Dan L. Duncan Building (CPB5.3505)

1515 Holcombe Blvd. Unit 1354

Houston, TX 77030

Co- Principal Investigator: Khandan Keyomarsi, MD

(Translational Sciences)

Co- Principal Investigator: Kelly Hunt, MD

Protocol Version: 6

IND No.: 138453

**Contents**

LIST OF ABBREVIATIONS AND DEFINITIONS OF TERMS……………………………...5

[35T**1.0**35T 35T**INTRODUCTION**35T 6](#_Toc486591094)

[35T1.135T 35TBackground35T 6](#_Toc486591095)

1.2 Mechanisms of Resistance to Endocrine Therapy and Rationale for the use of Autophagy inhibition with Hydroxychloroquine………………………………………..7

[35T1.335T 35TRationale for the Use of Pre-operative (Neoadjuvant) or “Window” Endocrine Therapy35T ……………………………………………………………………………………8](#_Toc486591096)

[35T1.435T 35TRisk/Benefit Assessment35T 10](#_Toc486591097)

[35T**2.0**35T 35T**STUDY AIMS AND OBJECTIVES**35T 10](#_Toc486591098)

[35T2.135T 35TPhase I Safety Component (metastatic disease) Objectives and Endpoints35T ……..10](#_Toc486591100)

[35T2.235T 35TPhase II Window/Neo-adjuvant Component Objectives and Endpoints35T 11](#_Toc486591104)

[35T**3.0**35T 35T**INVESTIGATIONAL PLAN**35T 12](#_Toc486591105)

[35T3.135T 35TOverall Design35T 12](#_Toc486591106)

[35TPhase I Safety Component:35T 12](#_Toc486591107)

[35TPhase II Pre-operative “Window” Component:35T 12](#_Toc486591108)

3.2 Trial Schemata………………………………………………………………………….. 12

[35T3.335T 35TDose Limiting Toxicity Definition35T 14](#_Toc486591109)

[35T3.435T 35TPhase II Part 1 (neo-adjuvant cohorts)35T 1](#_Toc486591110)5

[35T3.535T 35TPhase II Part 2 Window/Neo-adjuvant Component35T 1](#_Toc486591116)5

[35T3.635T 35TPhase II Patient Evaluability35T 15](#_Toc486591117)

[35T3.735T 35TReplacement of Patients35T](#_Toc486591118) 15

[35T3.835T 35TTarget Accrual35T](#_Toc486591119) 15

[35T**4.0**35T 35T**PATIENT SELECTION**35T 16](#_Toc486591120)

[35T4.135T 35TInclusion Criteria35T 16](#_Toc486591121)

[35T4.235T 35TExclusion Criteria35T 17](#_Toc486591122)

[35T**5.0**35T 35T**STUDY PROCEDURES AND SCHEDULE OF ASSESSMENTS**35T 18](#_Toc486591123)

35T5.1 Patient Identification…………………………………………………………………… 18

[35T5.235T 35TSchedule of Assessments35T 18](#_Toc486591124)

[35T5.2.135T 35T Baseline Assessments35T 20](#_Toc486591125)

[35T5.2.235T 35T Evaluation During Study Phase II35T 20](#_Toc486591126)

[35T5.2.335T 35T Treatment Duration35T 20](#_Toc486591127)

[35T5.2.3.1 Phase I35T 20](#_Toc486591128)

[35T5.2.3.2 Phase II35T 21](#_Toc486591130)

[35T5.2.4 35T 35TEnd of treatment visit and Follow up assessments35T 21](#_Toc486591131)

5.2.5 Early discontinuation………………………………………………………………21

5.2.6 Review of Systems and Physical Exam…………………………………………21

5.2.7 Laboratory Assessments………………………………………………………….21

[35T5.2.835T35T Imaging assessments Phase I35T 21](#_Toc486591132)

[35T5.2.9 35TImaging assessments Phase II35T 21](#_Toc486591132)

[35T5.335T 35TCorrelative Biomarker Studies35T 22](#_Toc486591136)

5.3.1 Biopsies, Tissue Handling and Analysis……………………………………….......22

5.3.2 Whole Blood/Plasma Biospecimens………………………………………….…….23

5.3.3 Ki67 Tumor Assay and Scoring……………………………………………………..23

5.4 Banked Biospecimens………………………………………………………………..24

[35T**6.0**35T 35T**INVESTIGATIONAL AND NON-INVESTIGATIONAL AGENTS**](#_Toc486591137)

[35T6.1 35T 35TPalbociclib35T 24](#_Toc486591138)

6.1.1 Description of palbociclib…………………………………………………………….24

6.1.2 Source of palbociclib………………………………………………………………….24

6.1.3 Preparation and dispensing………………………………………………………….24

6.1.4 Dosing and adjustments for palbociclib………………………………..…………...25

[35T6.235T 35THydroxychloroquine35T 2](#_Toc486591139)5

6.2.1 Description of hydroxychloroquine……………………………………………….…25

6.2.2 Source and dispensing of hydroxychloroquine…………………………………....26

6.2.3 Dosing and adjustments for hydroxychloroquine………………………………….26

[35T6.335T 35TLetrozole35T](#_Toc486591140) 26

6.4 Concomitant Medications………………...………………………………………………27

[35T**7.0**35T 35T**ADVERSE EVENT MONITORING AND REPORTING**35T 27](#_Toc486591141)

[35T7.135T 35TDefinition of an Adverse Event35T 27](#_Toc486591142)

[35T7.235T 35TSerious Adverse Event (SAE) Reporting35T 27](#_Toc486591143)

[35T](#_Toc486591144)

[35T7.335T 35TRecording of Adverse Events35T](#_Toc486591145) 29

[35T7.435T 35TAssessment of Adverse Events35T 29](#_Toc486591147)

[35T7.535T 35TCommunications between the Investigator and Supporting Company Pfizer35T 30](#_Toc486591148)

[35T**8.0**35T 35T**STATISTICAL CONSIDERATIONS**35T 30](#_Toc486591149)

**8.1 IND Office Reporting..**…………………………………………………………………..31

[35T**9.0**35T 35T**INSTITUTIONAL REVIEW, CONSENT, MEDICAL MONITORING AND RECORD KEEPING AND SAFETY MONITORING**35T 31](#_Toc486591150)

[35T9.1 Informed Consent35T 3](#_Toc486591151)1

[35T9.2 Institutional Review Board Approval35T 32](#_Toc486591152)

[35T9.335T 35TConfidentiality35T 32](#_Toc486591153)

35T [9.4 Study Medical Monitoring Requirements 32](#_Toc486591154)

[35T9.535T 35TData Collection35T 33](#_Toc486591155)

[35T**10**35T **REFERENCES** ……….…….............................................................................................3](#_Toc486591156)4

**11 APPENDICES**……………………………………………………………………………..37

Appendix A: Palbociclib (Ibrance) Highlights of Prescribing Information………………...37

Appendix B: Hydroxychloroquine (Plaquenil) Package Insert…………………………….69

Appendix C: Letrozole (Femara Tablets) Package Insert…………………………………77

**ABBREVIATIONS AND LIST OF DEFINITIONS OF TERMS**

**Abbreviation Term/Definition**

A

Akt A growth factor pathway component (serine-threonine kinase)

Alk Phos Alkaline phosphatase

ALT Alanine aminotransferase

ANC Absolute neutrophil count

AST Aspartate aminotransferase

ASCO/CAP American Society of Clinical Oncology/College of American Pathologists

CBC Complete blood count

CCCA Complete cell cycle arrest (Ki67≤ 2.7%)

CDK Cyclin-dependent kinase

DL Dose level

DLT Dose-limiting toxicity

DNA Deoxyribonucleic acid

ECOG Eastern Cooperative Oncology Group

ER Estrogen receptor

ESR1 Estrogen receptor-alpha gene

FDA Food and Drug Administration (United States)

FSH Follicle stimulating hormone

HCQ Hydroxychloroquine

HER2 Human epithelial receptor-2 (HER2/neu)

JAK/STAT Janus kinase/signal transducers and activators of transcription

Ki67 Kiel antigen, a cellular marker for proliferation

L Letrozole

LH Luteinizing hormonal

MBC Metastatic breast cancer

MCF7 A hormone receptor-positive breast cancer cell line

mg Milligrams

mTOR Mammalian target of rapamycin

P Palbociclib

p.o. By mouth

PR Progesterone receptor

PI3K Phosphoinositide 3-kinase

PIK3CA Phosphatidylinositol-4,5-Bisphosphate 3-Kinase Catalytic Subunit Alpha

Rb Retinoblastoma protein

RNA Ribonucleic acid

RP2D Recommended phase II dose

SEER Survey End Epidemiology Results

SMC Safety and Monitoring Committee

T. Bili Total bilirubin

ULN Upper limits of normal (Institutional)

U/S Ultrasound

- 1. **IIntroduction**

**1.1 Background**

- - 1. Hormone receptor-positive breast cancer

Breast cancer is the most common female cancer in the United States. It is estimated that 252,710 American women will be diagnosed with breast cancer and 40,610 will die from the disease in 2017.P^3^P A major cause of death in these patients is disease progression and incurable metastasis. The SEER database estimates that ~40,000 breast cancer patients a year either present at diagnosis (n=13,900) with metastatic disease or exhibit progression with metastatic disease, sometimes many years after completing therapy for early stage disease (n=36,000).P^4^P Of these, an estimated 30,000 have estrogen receptor (ER)-positive breast cancers.P^4^P Hence, patients with ER+ metastatic breast cancer (MBC) represent a majority of breast cancer who die of their disease, the vast majority due to metastatic progression.

- - 1. Therapies for hormone receptor-positive breast cancer

In early stage estrogen receptor-positive (ER+) breast cancer, the use of adjuvant endocrine therapy (tamoxifen or aromatase inhibitors) lowers the relative risk of recurrence at 10 years by 45 to 50% and that of mortality by breast cancer death by 30%.P^5^P In the metastatic setting endocrine therapy can induce responses and delay progression of disease. However, in the metastatic setting, it has been difficult to demonstrate a survival benefit of endocrine therapy. Few modern-day trials have compared endocrine therapy to no endocrine therapy in the absence of chemotherapy since treatment induces clear palliation. More recent trials comparing single-agent to combination endocrine therapy agents (e.g. aromatase inhibitors plus the ER down regulator fulvestrant) have yielded mixed results.P^6, 7^P In the last few years, the addition of biological therapies that were designed to address mechanisms of resistance or with demonstration of preclinical synergy have shown improvements in disease-free survival when added to endocrine therapy, but no impact on overall survival - although these trials were not designed with the statistical power to survival differences, with disease-free survival designated as the primary endpoint. The addition of the mTOR inhibitor everolimus doubled median disease-free survival from 4.1 to 10.6 months when added to the aromatase inhibitor exemestane as second-line therapy and was approved by the FDA with final survival analysis showing no significant difference in that outcome (median survival of 31.0 vs. 26.6 months).P^8, 9^P Other biological agents such as PI3 kinase inhibitors, histone deacetylase inhibitors and cyclin-dependent kinase CDK) inhibitors are being actively tested in first, second and later lines of therapy - so far CDK 4/6 inhibitor palbociclib has been approved by the FDA as outlined in the next section.

In summary, this is a novel investigator initiated study funded by CPRIT grant which aims to evaluate the safety and tolerability (phase I) of adding HCQ to continuous low dose palbociclib and letrozole in metastatic HR+, HER2- breast cancer and thereafter determine whether the inhibition of autophagy with HCQ can decrease proliferation index in a window study (Phase II) in postmenopausal women with stage I-III HR+/HER2- breast cancer. The scientific rationale is based on preclinical work that has been done by the trial investigators and published as is well described in the protocol. Further the trial has been vetted through our departmental HR positive working group as well our department’s protocol review process. A multidisciplinary team of collaborators are involved and it is well funded. Finally, we have had success with accrual demonstrated to other window trials, with several such studies ongoing without any concerns of low accrual. As noted in the trial prioritization, this is #3 of 5 trials in the HR+, HER2- patient population but the other ongoing or planned trials are expected to have little if any overlap with the activation of protocol 2017-0071.

- - 1. Palbociclib and use in breast cancer

Palbociclib is a potent and specific cyclin-dependent kinase 4/6 (CDK4/6) inhibitor and anti-proliferative agent that induces G1 arrest and prevents breast cancer cell growth, most notably in ER+ cell lines and other preclinical models, which led to the clinical development of this class of inhibitors in hormone receptor-positive breast cancer.P^11-13^P Palbociclib was approved by the FDA in February, 2015 based on improved time to disease progression in the first and second-line settings for ER+/HER2-negative metastatic breast cancer (MBC).P^14-17^P In the pivotal first-line Phase III trial, progression-free survival (primary endpoint) was improved from 14.5 to 24.8 months with the addition of palbociclib to the standard first-line aromatase inhibitor letrozole.P^15^P In the second-line setting, the addition of palbociclib to the standard second-line estrogen downregulate fulvestrant in a Phase III trial also demonstrated a significant improvement in time to progression from 3.8 to 9.2 months.

However in the first line setting, 66% of patients on the palbociclib experienced grade 3 and 4 neutropenia, and adverse events necessitate a palbociclib dose reduction in 36% and discontinuation in 7.4% of patients.P^15^P Additionally, palbociclib must be dosed with a 7-day break, which was shown to be associated with a proliferative burst in a separate neoadjuvant serial biopsy study.P^18^P Also, a precise biological mechanism of palbociclib’s action is still unknown and there are no known independent biomarkers to predict response and/or resistance to palbociclib – many patients do not respond to therapy, and most importantly, virtually all eventually develop resistance. Biomarkers that may influence response to endocrine therapy and CDK inhibitors including estrogen receptor-alpha (*ESR1*) and *PIK3CA* mutations, loss of the retinoblastoma protein function, proliferative antigen Ki67 expression, *cyclin D1* gene amplification or loss of the cyclin-dependent kinase inhibitor p16 have not shown to be related to palbociclib-associated response or time to progression.P^13, 17, 19^P Importantly, no survival benefit yet observed in either of the larger randomized trials or smaller Phase II trial, although these trials were not powered for survival differences and diversity of therapies that patients may receive in their subsequent management may cloud future survival update analyses.

**1.2 Mechanisms of Resistance to Endocrine Therapy and Rationale for the use of Autophagy inhibition with Hydroxychloroquine**

While specific mechanisms of resistance to palbociclib have not been well characterized, there are several mechanisms of resistance to conventional endocrine therapies such as tamoxifen and aromatase inhibitors, which include activation of receptor tyrosine kinase signaling pathways (notably the PI3K/Akt/mTOR pathway), activation mutations in ESR1 and other more general alterations such as epigenetic regulation of gene expression other intersecting pathways like NFKB and JAK/STAT.P^20^P As described earlier, mTOR and CDK4/6 inhibition with everolimus and palbociclib are now approved as biological agents in combination with endocrine therapy, while strategies using inhibitors of PI3K, Akt, histone deacetylase are undergoing testing, none so far has been able to induce durable enough responses to affect survival, and clinical resistance still emerges with all available therapies.


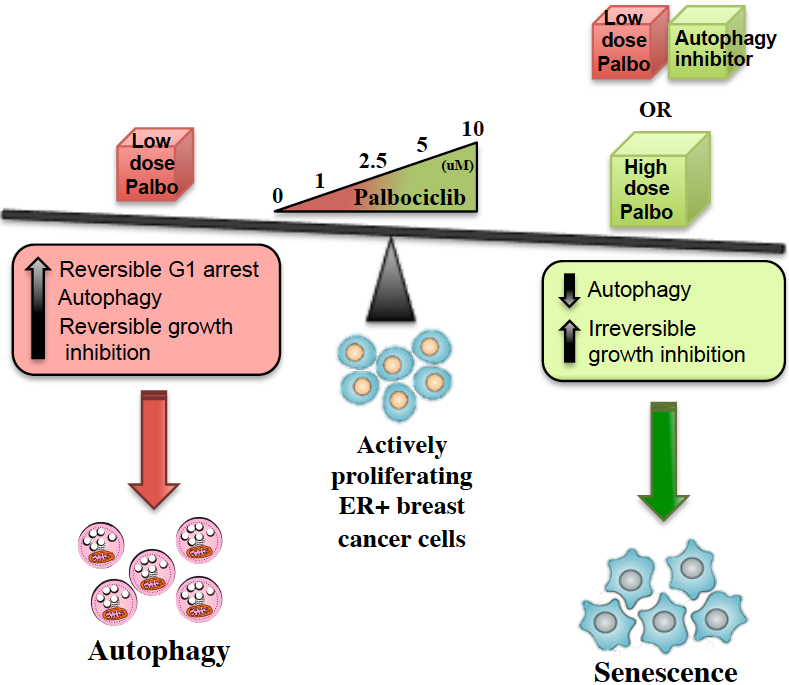
Our laboratory research group, led by Khandan Keyomarsi, PhD, Professor of Experimental Radiation Oncology at MD Anderson, has an extensive investigational track record in area the cell cycle pathways in breast cancer. We have shown that at low concentrations of the palbociclib, ER+ breast cancer cells arrest in the G1 phase of the cell cycle, but this arrest is reversible due to the activation of autophagy. However, at high concentrations of palbociclib, the cells are arrested irreversibly in G1, do not undergo autophagy and instead undergo senescence. The low concentration of palbociclib are on-target and are specific for inhibition of CDK4 and CDK6, as shown with siRNA assays where CDK4 and CDK6 where knocked down. The high concentrations of palbociclib, induce senescence, but these are off target effects of the drug. We also demonstrated that if we combine palbociclib with an autophagy inhibitor such as hydroxychloroquine (HCQ), we can achieve senescence at a much lower (i.e. on- target) and continuous dosing of palbociclib, in both *in vitro* and *in vivo* models. Moreover, concomitant treatment with HCQ and palbociclib can mediate a synergistic response in ER+ xenograft (MCF7) mouse tumor volume and weight compared to palbociclib alone. More strikingly, the tumor volumes with combination treatment did not increase even after the treatment was stopped, while the tumor volumes with either palbociclib or HCQ alone continuously increased during both the treatment and recovery phases of the experiment. Combination treatment showed the desired and expected impairment in autophagic flux. Decrease in Rb phosphorylation, a direct readout of CDK 4/6 inhibition, was more pronounced with the addition of HCQ. These results suggest that autophagy inhibition significantly improves the efficacy of low dose (on target) palbociclib *in vivo* and facilitates the induction of an irreversible tumor growth inhibition. We propose that palbociclib activates the autophagy pathway to protect ER+ breast cancer cells from palbociclib-induced senescence and inhibition of autophagy sensitizes cells to lower doses of palbociclib in vitro and in vivo (Figure 1).

**Figure 1: Model for Hydroxychloroquine to Inhibit Autophagy and Potentiate Palbociclib**

We therefore propose using HCQ to inhibit autophagy in patients treated with low dose (on target) palbociclib and letrozole to demonstrate enhanced anti-proliferative effect. An anti-proliferative effect of endocrine therapy using quantification of the proliferative Ki67 antigen has been validated as a long-term predictor of outcome. HCQ is a commonly used and generally safe drug used to treat malaria and as a remittive agent for various autoimmune diseases (see more expanded description in Section 6.2). This trial will therefore provide evidence of a promising therapeutic effect with lower dose palbociclib plus letrozole for a greater therapeutic effect with less side effects. This is needed before proceeding with a more definitive trial that could be potentially practice-changing.

**1.3 Rationale for the Use of Pre-operative (Neoadjuvant) or “Window” Endocrine Therapy** **80**

We have chosen to perform this trial using the combination of letrozole, palbociclib and HCQ in the pre-operative (neoadjuvant) “window” setting after careful deliberation internally and advice from both internal and external advisory board members during the process of a grant proposal submission that led to the successful funding of both the laboratory and clinical trial aims of this project. However, the initial lead-in Phase I safety trial that includes HCQ dose escalation with the study regimen of continuous low dose palbociclib and letrozole and the determination of the recommended Phase 2 dose (RP2D) for HCQ will be conducted in patients with advanced metastatic breast cancer, followed by activation of the neoadjuvant/window trial.

The following rationale support this approach:

- Palbociclib is already FDA-approved for metastatic breast cancer with an excellent safety record. It is currently in several clinical trials in both the neoadjuvant and adjuvant settings.
- Treatment will include letrozole, a standard endocrine therapy as is used clinically for early stage breast cancer.
- HCQ is an approved drug commonly used for malaria and autoimmune disease and particularly safe when used at the doses and timeframes proposed (see more expanded description in Section 6.2).
- This model allows for safe biopsies as needed for the correlative tissue aims and eliminates artifact and variability due to prior therapies as all patients will be previously untreated.
- The design gives patients and their physicians the option of a brief “window” trial with 4 weeks on study drug (2 weeks on low dose palbociclib plus letrozole, then 2 weeks with the addition of HCQ) then to proceed with surgery or a research biopsy if additional standard of care neoadjuvant therapy is indicated, and then followed by standard of care.
- The very first exposure to the combination will be performed in a cohort of patients with advanced breast cancer using a standard “3+3” Phase I HCQ dose escalation scheme in combination with the study regimen of low dose continuous palbociclib with letrozole. Once safety is established at the specific HCQ dose level, the corresponding arm of the Phase II neoadjuvant/window trial will be activated. This approach will maximize safety by only moving to early stage curable population once safety of the study regimen is established in the advanced setting.

There is ample clinical experience and clinical trial to support the use of neoadjuvant endocrine therapy as method to downstage tumors and allow more patients to undergo breast-conserving surgery.P^21^P Additionally, the neoadjuvant model is a well-established research tool to assess newer drugs when added to endocrine therapy to evaluate both clinical as well as biomarker based-responses.P^22^P For hormone receptor-positive and HER2-negative breast cancer, the use of change in the proliferative antigen Ki67 pre to post treatment has been extensively validated as index that correlates with the likelihood of achieving a complete pathological response and with longer term outcomes specifically in trials.P^23-31^P The key metrics using this model are obtained after 4 weeks of low dose Palbociclib and HCQ (safety) in the Phase I portion and after 2 weeks of low dose palbociclib and then 2 additional weeks with the addition of HCQ (efficacy) in the Phase II portion (see Sections 2.0 and 3.1).

Therefore, patients can come off study after 4 weeks and proceed with standard therapy (“window” approach), which would include standard neoadjuvant endocrine therapy (typically aromatase inhibitor), neoadjuvant chemotherapy if the patient is known to have the stage, grade and other indices that warrant chemotherapy. Alternatively, they can proceed with definitive breast/axillary node surgery. In addition, if the biopsy after both 2 and 4 weeks show suppression of proliferative index that is used as the main endpoint of the study (see Section 2.2.1), patient can stay on therapy for the typical full neoadjuvant endocrine therapy course which is typically 16-24 weeks. These pragmatic alternatives will maximize accrual and allow patients and their physicians to opt for the best path that matches the patient’s clinical situation.

- 1. **Risk/Benefit Assessment**

Patients on this study will be receiving standard of care therapy (endocrine therapy with letrozole) and palbociclib, an FDA-approved and relatively non-toxic therapy that doubles time to progression in the metastatic setting, but not approved in the adjuvant or neoadjuvant setting (see additional description of palbociclib in Section 6.1). HCQ is an approved drug used for malaria and autoimmune disorders that also had a very good safety profile (see additional description of HCQ in Section 6.2). One concern of retinal toxicity that is typically seen only after prolonged exposure, will be mitigated by baseline ophthalmological exam covered by the study funding, and exclusion of those who are felt to be at risk for retinal toxicity as established by the by the American Academy of Ophthalmology to exclude preexisting maculopathy.P^32^P In order to exclude unexpected toxicities of the proposed combination in patients with early stage breast cancer who may have a reasonably good prognosis and chance of cure, a run-in Phase I safety study will be conducted in patients with metastatic breast cancer who are also candidates for standard palbociclib and letrozole therapy. The experimental regimen proposed for the window/neoadjuvant protocol, low dose continuous palbociclib and standard dose letrozole will be used, in combination with increasing doses of HCQ.

The benefits of participation of this trial cannot be fully ascertained, but the addition of palbociclib has been shown to achieve a high rate of complete cell cycle arrest, a surrogate biomarker of long-term benefit.P^18^P While short term exposure (4 weeks) may not affect outcome, patients who stay on study for a total of 16-24 weeks prior to surgery may have a greater degree of down staging and more conservative surgery (e.g. breast conservation, sentinel node biopsy as opposed to axillary dissection).

1. **80T** **STUDY AIMS AND OBJECTIVES**

**80TThe overall study aim is to determine whether autophagy inhibition with HCQ can significantly decrease proliferation index, a validated surrogate of long term outcome, when added to a continuous low dose palbociclib and letrozole.**

- 2. **80TPhase I Safety Component (metastatic disease) Objectives and Endpoints**
     1. **80TThe primary objective of the Phase I portion in the metastatic setting is to determine the safety of adding HCQ to continuous low dose palbociclib and letrozole and to determine the recommended phase II dose for HCQ for the subsequent Phase II study.**

**80TPrimary endpoint is safety, to be assessed continuously using CTCAE V4.03, with physical examination and laboratory assessments as indicated on the study schedule. Dose-limiting toxicities as defined in Section 3.3.**

**80T2.1.2 The secondary objective is to determine the response rate and clinical benefit rate at 8 weeks of the assigned HCQ dose plus continuous low dose palbociclib and letrozole.**

- 1. **80TPhase II Window/Neo-adjuvant Component Objectives and Endpoints**
     1. Phase II - Part 1 Primary Objectives and Endpoints

To determine the dose responsiveness of 2 dose levels (400 mg and RP2D) of hydroxychloroquine added to low dose palbociclib and letrozole on pre and post HCQ breast tumor proliferation index (Ki67), autophagy, senescence and cell cycle control.

- - 1. Phase II - Part 1 Secondary and Additional Objective and Endpoints

Determine longer term clinical tumor responsiveness (tumor volume) and tumor biomarker indices (for patients who have extended pre-operative therapy, maximum 24 weeks).

Perform exploratory studies on blood-based tumor protein, DNA and RNA biomarkers with a focus on pathways of cell proliferation, autophagy, senescence and cell cycle control.

- - 1. Phase II Part 2 Primary Objective and Endpoints

To determine whether hydroxychloroquine added to low dose palbociclib and letrozole can increase the proportion of patients whose tumors achieve complete cell cycle arrest (CCCA, defined as the Ki67 ≤ 2.7%) comparing T2 to T1 as shown in Figure 2, see Section 5.3.4).

The primary endpoint is the increase in percentage of all patients who achieve CCCA with low dose palbociclib and letrozole (at week 2, time T1) compared to that of low dose palbociclib and letrozole with the addition of HCQ (at 5 weeks, day 29, time T2).

A previously conducted trial of neoadjuvant aromatase inhibitor (anastrozole) plus standard dose palbociclib estimated the CCCA probability to be 44% with aromatase inhibitor alone and powered their study to detect a 50% increase of 44% to 66%P^18^P. A 26% CCCA probability was seen with anastrozole alone and 86% with the addition of standard dose palbociclib. We will aim for a CCCA reference probability of 50% with low-dose palbociclib + letrozole (T1) and a CCCA probability of 75% with the addition of HCQ (see Section 8 for statistical plan and samples size calculations for the Phase II portion).

- - 1. Phase II Part 2 Secondary Objectives and Endpoints
- To determine the impact of adding hydroxychloroquine to low dose palbociclib and letrozole on breast tumor indices of proliferation, autophagy, senescence, cell cycle control and other intersecting pathways.
- Determine longer term clinical tumor responsiveness and tumor biomarkers indices (for patients who have extended pre-operative therapy, maximum 24 weeks).
- To determine the dose responsiveness of HCQ (400 mg vs. RP2D) on the primary (proportion with CCCA) and secondary clinical/biological endpoints
- To perform exploratory studies on blood-based tumor protein, DNA and RNA biomarkers.
- Obtain additional safety information for the combination of low dose palbociclib, letrozole and hydroxychloroquine.

1. **80T** **INVESTIGATIONAL PLAN**

   2. **80TOverall Design**

This is an open label Phase I/II prospective interventional trial with a safety component in metastatic disease followed by a “window” trial component in the neo-adjuvant (pre-operative) setting.

**Phase I Safety Component:**

The Phase I is designed to evaluate the safety and tolerability of adding HCQ to continuous low dose palbociclib and letrozole in the metastatic setting and to determine the recommended phase 2 dose for HCQ. A standard 3+3 dose escalation designed will be employed.

This safety component will be completed before the initiation of the window trial (Phase II) in the pre-operative setting.

**Phase II Pre-operative “Window” Component:**

The “window” component of the trial evaluates the combination of pre-operative palbociclib plus letrozole followed by the addition of hydroxychloroquine (HCQ) for postmenopausal patients with Stage I-III estrogen receptor-positive and HER2-negative breast cancer (Figure 2).

Correlative studies using baseline and follow up breast biopsies and blood work are included in the research plan. The Phase II portion will assess the change in the percentage of all patients who achieve CCCA when adding HCQ to the palbociclib/letrozole combination.

- 1. **80TTRIAL SCHEMATA**
     1. **Trial Overview Schema Design and Flow**


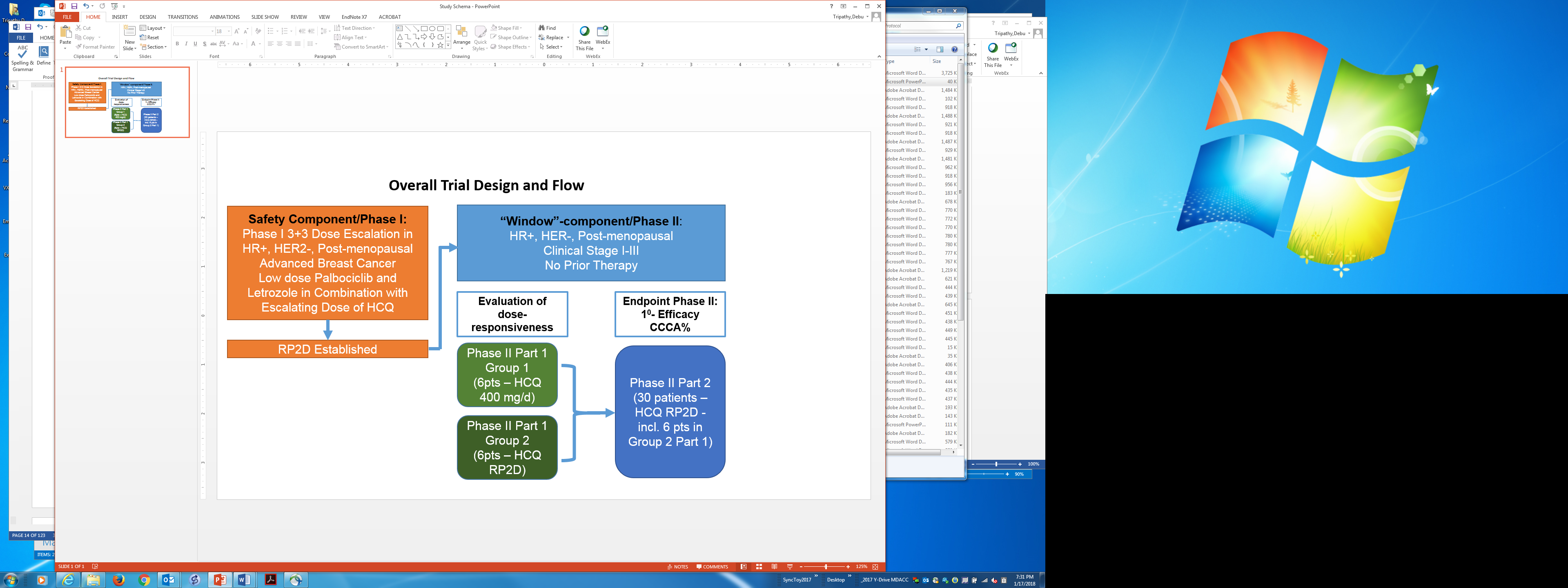


**Figure 1: General schema for both phases of the trial.** The run-in Phase I safety study done in the metastatic setting will establish safety and determine the Phase II dose. The Phase II study will assess two doses of HCQ (400 mg in 6 patients and RP2D in 30 patients) added to continuous low dose palbociclib plus letrozole and assess proliferative index (Ki67) pre and post HCQ administration.

- - 1. **80TWindow Component Schema and Design**


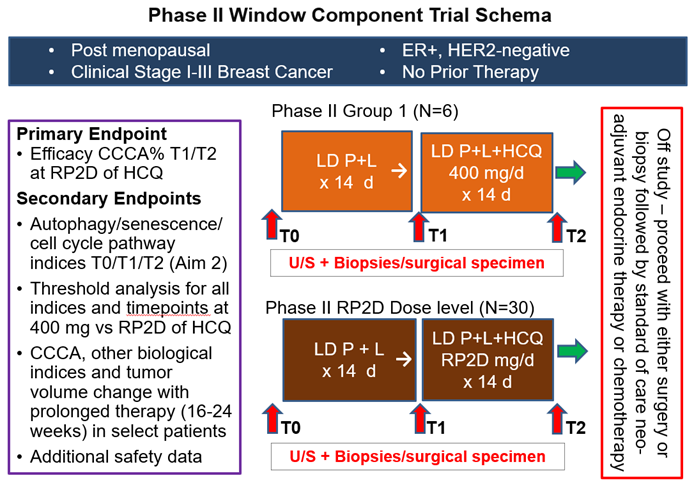


**80T**

**FIGURE 2. Schema for pre-operative “window”/neoadjuvant trial.** Part 1 dose responsiveness evaluation at 400 mg/d HCQ followed by Part 2 at RP2D HCQ using same design and biopsy time points (T0 [baseline/pretreatment, T1 [after 2 weeks P+L], T2 [after addition of HCQ]), with option to proceed with surgery right after T2 biopsy or up to 24 weeks from initiation of therapy. P=continuous low dose palbociclib at 75 mg daily L=letrozole at 2.5 mg daily; HCQ=hydroxychloroquine continuous daily at assigned dose; N=number of patients; U/S=ultrasound; RP2D-recommended Phase II dose (of HCQ).

80T**Design for the Safety Component/Phase I**

**80TA cycle will be defined as 28 days, with dose limiting toxicity (DLT) assessed over the first cycle.**

**80TThe dose of letrozole and palbociclib will be fixed for the safety cohorts at:**

**80T• Palbociclib: 75 mg capsule taken orally once daily on a continuous basis.**

**80T• Letrozole: 2.5mg/d taken orally once daily continuously.**

**80**

**Hydroxychloroquine Dose Escalation Scheme**

| Dose Level (DL) | Hydroxychloroquine | Letrozole | Palbociclib |
| --- | --- | --- | --- |
| 0 (Starting Dose) | 400 mg/d | 2.5 mg daily | 75 mg daily (continuous) |
| +1 | 600 mg/d |  |  |
| +2 | 800 mg/d |  |  |

The dose escalation portion will use a 3+3 dose escalation design to evaluate escalating dose levels of HCQ in combination with palbociclib and letrozole (see dose level table above). Three patients will be enrolled at the first dose level (DL 0, 400 mg daily) of the triplet combination. At least 3 patients must complete a treatment cycle (28 days) with the triple combination and undergo toxicity assessment before any additional patients are enrolled into higher dosing cohorts. Intrapatient dose escalation or change of dosing schedule is not allowed. At least 6 evaluable patients are to be treated at a dose level for a RP2D to be declared.

- If 0 out of 3 patients in a dose level experience a DLT in the defined timeframe another 3 patients will be enrolled at the next higher dose level.
- If no more than 1 patient experienced a DLT at a dose level, then more patients will be added to that cohort to increase the cohort up to six patients at the same dose level
- If no more than one of the first 6 patients treated experiences a DLT, then 3 patients will be enrolled at the next higher dose level.
- If two or more of the first 6 patients treated within a cohort experience DLT, then this dose level will be considered not tolerated and the dose escalation will be halted. If two DLTs are seen in any cohort, no further patients will be enrolled at that level and next level lower will be declared the RP2D.

The RP2D is defined as the highest dose level at which 6 patients have been treated with at most 1 instance of DLT.

If two DLTs are seen at the starting dose of 400 mg (DL 0), then this treatment will be declared to not be feasible as doses of HCQ < 400 mg may be too low for the intended effects.

The Principal Investigator will monitor safety throughout the study, review patient data and make decisions prior to advancing to the next dose level, approve dose escalation, and will also declare the establishment for RP2D and declare when the Phase II part of the study can commence.

- 1. **80TDose Limiting Toxicity (DLT) Definition**

A DLT will be defined as:

- Any death not clearly due to the underlying disease or extraneous causes
- Any grade 3 or higher non-hematologic toxicity
- Liver function abnormalities meeting Hy's law
- Neutropenic fever
- Grade 4 neutropenia or thrombocytopenia > 7 days
- Grade 3 or higher thrombocytopenia with bleeding
- Grade 3 or higher nausea/vomiting or diarrhea ≥ 72 hours with adequate antiemetic and other supportive care
- Grade 3 or higher fatigue ≥ 1 week
- Grade 3 or higher electrolyte abnormality that lasts >72 hours, unless the patient has clinical symptoms, in which case all grade 3 or higher electrolyte abnormality regardless of duration should count as a DLT.

All AEs of the specified grades should count as DLTs except those that are clearly and incontrovertibly due to disease progression or extraneous causes.

The decision to open the Phase II window part of the trial will be made after RP2D has been established. All Safety Data will be reviewed by the Principal investigator and will be used to determine whether the study proceeds to the “window” Phase II trial in the neo-adjuvant setting.

- 1. **80TPhase II Component (Window/Neo-adjuvant Cohorts)**

**80TThe “window” component of the trial evaluates the combination of pre-operative palbociclib plus letrozole followed by the addition of hydroxychloroquine (HCQ) for postmenopausal patients with Stage I-III estrogen receptor-positive and HER2-negative breast cancer (Figure 2).**

**80TCorrelative studies using baseline and follow up breast biopsies and blood work are included in the overall research grant. The Phase II portion will assess the change in the percentage of all patients who achieve CCCA when adding HCQ to the palbociclib/letrozole combination.**

- 1. **80TPhase II Window/Neo-adjuvant Component – Dose Cohorts (Parts 1 and 2)**

**80TTo estimate the dose responsiveness of HCQ in the main outcome measures as stated on Section 2.2.3, two groups will be enrolled at the HCQ levels as shown below:**

| Dose Level  Groups | Number of  patients | Hydroxychloroquine  dose | Letrozole  dose | Palbociclib dose |
| --- | --- | --- | --- | --- |
| Part 1 | 6 | 400 mg/d p.o. | 2.5 mg/d p.o. | 75 mg capsule p.o. daily  continuously, 28-day cycle |
| Part 2 | 30 | RP2D |  |  |

An initial group of 6 patients (part 1) will be enrolled at an HCQ dose of 400 mg daily sequentially, and then a second group of 30 patients (part 2) will be enrolled at the HCQ dose defined in the Phase I portion as RP2D. The main efficacy endpoint statistical plan is based on the 30 patients enrolled in part 2 as described in Section 8.0.

The dose of palbociclib for both parts 1 and 2 is fixed at a dose of 75 mg orally daily continuously without a break. Letrozole and palbociclib are given for the first 2 weeks, then after the second biopsy (T2), HCQ at the assigned dose will be added. After 4 weeks (2 weeks of HCQ) (T2), the primary endpoint will have been obtained with analysis of the surgical specimen (or research biopsy at that timepoint if additional neoadjuvant endocrine therapy or chemotherapy is indicated as standard of care. Details on dosing and dose adjustments are shown in Sections 6.1.4 and 6.2.3. Using the same population, treatment and biopsy schema, 30 patients will be enrolled for the phase II Stage 2 portion of the trial.

- 1. **Phase II Patient Evaluability**

Patients who meet the eligibility criteria and complete a full 4 weeks of therapy (14 days of low-dose palbociclib plus letrozole and 14 days of dose palbociclib plus letrozole with HCQ) will be considered evaluable for Phase II. In addition, the completion of biopsies at T0, T1 and T2 with interpretability of Ki67 are required for evaluability. Baseline Ki67 >5% is required to enhance the interpretability of CCCA fraction change from T1 to T2.

- 1. **Replacement of Patients**

Patients who are not evaluable for dose-limiting toxicity in the Phase I portion and those not evaluable for Ki67 at T0, T1 and T2 in the Phase II portion will be replaced.

- 1. **Target Accrual**

A total of 54 patients will be enrolled to the study. This includes 2 to 18 patients (up to 6 per dose cohort at 400, 600 and 800 mg of HCQ daily and 6 at the RP2D, but as few as 2 patients in the unlikely event that the first 2 patients experience DLT at the lowest dose of HCQ (400 mg daily) and the study is halted.) in the Phase I metastatic portion, 6 patients for neo-adjuvant Phase II Part 1 and 30 patients for the Phase II Part 2 portion (25 needed for statistical plan and 5 expected to drop out). This does not account for replaced patients (as stated in Sections 3.6 and 3.7).

1. **80T** **PATIENT SELECTION**

   2. **Inclusion Criteria**

Patient eligibility criteria include all of the following:

1. Signed written informed consent.
2. Diagnosis of estrogen positive breast cancer, estrogen receptor-positive and HER2-negative by ASCO/CAP criteriaP^1, 2^P.
3. ECOG performance status 0-1.
4. Female and age ≥ 18 years and postmenopausal defined by:
   1. Age ≥ 55 years and 1 year or more of amenorrhea.
   2. Age < 55 years and 1 year or more of amenorrhea with LH and/or FSH levels in the postmenopausal range.
   3. Age < 55 with prior hysterectomy but intact ovaries with LH and/or FSH levels in the postmenopausal range
   4. Chemotherapy or medically induced ovarian suppression with 1 year or more of amenorrhea and with LH and/or FSH levels in the postmenopausal range.
   5. Status after bilateral oophorectomy (≥ 28 days prior to first study treatment).
5. Adequate hematological, renal, hepatic function defined as follows:
   1. ANC ≥ 1500 cells/μl.
   2. Platelet count ≥ 100,000/μl.
   3. Serum creatinine concentration < 1.5 x ULN.
   4. Bilirubin level < 1.5 x ULN.
   5. Aspartate aminotransferase (AST) or alanine aminotransferase (ALT) <3 x ULN or alkaline phosphatase ≤ 2.5 ULN.
6. Metastatic cohorts (Phase I): Diagnosis of Stage IV estrogen positive breast cancer, estrogen receptor-positive and HER2-negative by ASCO/CAP criteriaP^1,2^ P.
7. Metastatic cohorts (Phase I): Must be a candidate for treatment with CDK4/6 inhibitor and endocrine therapy with an aromatase inhibitor as standard of care.
8. Metastatic cohorts (Phase I): No prior exposure to CDK 4/6 inhibitors.
9. Neoadjuvant cohorts (Phase II): Diagnosis of Stage I-III estrogen positive breast cancer, estrogen receptor-positive and HER2-negative by ASCO/CAP criteriaP^1, 2^P. If Stage I, clinical tumor size must be ≥1.5 cm.
10. Neoadjuvant cohorts (Phase II): Baseline tumor Ki67 > 5%.
11. Neoadjuvant cohorts (Phase II): Surgical candidate and appropriate for pre-operative endocrine therapy.
    1. **Exclusion Criteria**

Patient exclusion criteria include any of the following:

1. Prior exposure to CDK 4/6 inhibitor therapy
2. History of retinal disease or active visual disturbances (normal baseline study-specified retinal exam required).
3. Acute illness, including infections requiring medical therapy, known bleeding diathesis or need for anticoagulation.
4. Treatment with any of the following medications within 4 weeks before the baseline diagnostic biopsy is taken:
5. Oral estrogens, including hormone replacement therapy (but prior depot estrogen use not allowed).
6. Investigational agents (or 5 half-lives, whichever is longer).
7. Required concomitant use of any drug that is a strong CYP3A inhibitor or inducer.
8. Psychological, familial, sociological or geographical conditions that do not permit compliance with the study protocol.
9. Life expectancy of less than 6 months.
10. Pregnancy, lactation or planning to be pregnant.
11. Neo-adjuvant cohorts (Phase II): Prior therapy for breast cancer (medical, surgical or radiation therapy)
12. Neo-adjuvant cohorts (Phase II): Clinical T4 disease.
13. Neo-adjuvant cohorts (Phase II): Inoperable or metastatic (Stage IV) breast cancer based on standard evaluation.
14. **80TSTUDY PROCEDURES AND SCHEDULE OF ASSESSMENTS**
    1. **80TPatient Identification**

Eligible patients will be referred by members of the MD Anderson Department of Breast Medical Oncology or screened through electronic medical records and our Departmental database. Process for consent is detailed in Section 9.

- 1. **80TSchedule of Assessments**

Table 1 – Study Procedures and Assessments Phase I

|  | Baseline | C1W1 | C1W2 | C1W3 | C1W4 | C2W5 | C2W6 | C2W7 | C2W8 | C3  W1 | C4+  W1 | Off Study |
| --- | --- | --- | --- | --- | --- | --- | --- | --- | --- | --- | --- | --- |
| Day |  | D1 | D8 | D15 | D22 | D1 | D8 | D15 | D22 | D1 | D1 |  |
| Scheduling timeframe | -14d | +/- 2 | +/- 2 | +/- 2 | +/- 2 | +/- 2 | +/- 2 | +/- 2 | +/- 2 | +/- 2 | +/- 2 | Within 30 D |
| Laboratory | XP^A^ | X | X | X | X | X | X | X | X | X | X | X |
| Adverse event assessment) |  | X | X | X | X | X | X | X | X | X | X | X |
| Physical exam/ Weight/Vital signs | XP^A^ | X | X | X | X | X | X | X | X | X | X | X |
| ECOG performance status evaluation | X |  |  |  |  |  |  |  |  |  |  |  |
| Ophthalmologic Exam | XP^B^ |  |  |  |  |  |  |  |  |  |  | X |
| Imaging Assessments | XP^A^ |  |  |  |  |  |  |  | X^C^ |  |  |  |
| Tumor Biopsy | XP^C^ |  |  |  |  | XP^C^ |  |  |  |  |  |  |
| Palbociclib |  | X | X | X | X | X | X | X | X | X | X |  |
| HCQ |  | X | X | X | X | X | X | X | X | X | X |  |
| Letrozole |  | X | X | X | X | X | X | X | X | X | X |  |

U

Table 2 – Study Procedures and Assessments Phase II

|  | Baseline | W1 | W2 | W3 | W4 | W5 | Off Study |
| --- | --- | --- | --- | --- | --- | --- | --- |
| Day |  | D1 | D8 | D15 | D22 | D29 |  |
| Scheduling Window | -14 D | +/- 2 | +/- 2 | +/- 2 | +/- 2 | +/- 2 | Within 30 D |
| Laboratory | XP^A^ | X | X | X | X | X | X |
| Adverse event assessment) |  | X | X | X | X | X | X |
| Physical Exam/ Weight/Vital Signs | XP^A^ | X | X | X | X | X | X |
| ECOG performance status evaluation | X | X | X | X | X | X | X |
| Ophthalmologic Exam | XP^B^ |  |  |  |  |  | X |
| Breast ultrasound | XP^A^ |  |  |  |  | X |  |
| Research blood | XP^A^ |  |  | X |  | X | X |
| Biopsy (or surgery) | XP^A^ |  |  | X |  | XP^D^ |  |
| Low dose palbociclib + letrozole |  | X | X | X | X |  |  |
| HCQ |  |  |  | X | X |  |  |

UAbbreviations:U W=week (week 1 is the same as day 1 prior to starting palbociclib); HCQ = hydroxychloroquine; D = day; W = week

ULaboratory assessments:U Baseline, then weekly x 8 for Phase I and x 4 for Phase II, then as indicated until off study labs include CBC/differential, electrolytes, BUN, creatinine, glucose, albumin, AST, ALT, total bilirubin, alkaline phosphatase. Baseline only: PT, PTT, urinalysis with microscopic exam. Baseline urine or serum pregnancy test for both Phase I and II.

USuperscripts:U

A= Phase I: Laboratory assessment must be done within 14 days of C1 D1. Any imaging assessments already completed during the regular work-up of the patient within 28 days prior to starting study treatment, including before signing the main study ICF can be considered as the baseline images for this study; Phase II: done within 2 weeks prior to starting therapy (low dose palbociclib/letrozole).

B= done within 8 weeks prior to starting study medication

C= Phase I – baseline biopsy can of metastatic tumor pre-treatment or primary lesion (optional) or can be from archival tissue if available (either primary or metastasis). Week 5 biopsy (optional) can be +/- 1 week. Phase II – biopsies are mandatory- third biopsy done either at W5 for patients going on to surgery at that time, for those waiting until between weeks 20-24, biopsy done pre-operatively. Patients who know in advance that they will proceed with surgery at T2 will not need a biopsy and biomarkers will be measured on the surgical specimen. Imaging will be done at end of Cycle 2 (Week 8) +/- 1 week (prior to cycle 3 drug), and every 8-12 weeks (+/- 2 weeks) thereafter

D= Phase II – Sample of surgical specimen (or core needle biopsy if patients is going on to standard of care neoadjuvant endocrine or chemotherapy) will be taken for analysis as outlined in Section 5.3.

- - 1. **80TBaseline Assessments**

All patients will undergo a baseline standard evaluation for newly diagnosed breast cancer including (note that some baseline tests can be done prior to day 1 (week 1) as shown on Tables 1 and 2):

- Review of systems all organ systems will be examined.
- Physical examination, vital signs, weight, height, and evaluation of ECOG performance status.
- Standard of care pathological diagnosis of breast cancer, imaging (Phase II: breast ultrasound; Phase II: CT, MRI) and clinical staging.
- Laboratory studies: CBC/differential, electrolytes, BUN, creatinine, glucose, albumin, AST, ALT, Total Bilirubin, Alkaline phosphatase, PT, PTT.
- Urinalysis
- Ophthalmological exam per latest guidelines of the American Academy of Ophthalmology (AAO) for baseline screening with HCQ treatment.
- Fresh Tumor Biopsy (Phase II only).
- Research blood draw (Phase II only).
- Baseline urine or serum pregnancy test for both Phase I and II.

| **ECOG performance status** |
| --- |
| **Grade ECOG status** |
| 0 - Fully active, able to carry on all pre-disease performance without restriction.  1 - Restricted in physically strenuous activity but ambulatory and able to carry out work of a light or sedentary nature e.g., light housework, office work.  2 - Ambulatory and capable of all self-care but unable to carry out any work activities. Up and about  more than 50% of waking hours.  3 - Capable of only limited self-care, confined to bed or chair more than 50% of waking hours.  4 - Completely disabled. Cannot carry on any self-care. Totally confined to bed or chair.  5 - Dead |

- - 1. **80TEvaluation During Study Phase II**

**80TPatients will be evaluated with an organ-specific review of systems and physical exam at weeks 2, 3, 4, and those who choose and are eligible to go beyond the “window period” (as described in Sections 1.3, 3.2.2 and 5.2-Table 2) and stay on study until definitive surgery at week 16 to 24 will undergo additional evaluation at weeks 6, 8 and every 4 weeks thereafter until the last pre-operative visit between week 16 and 24 at the discretion of the medical and surgical oncologist as shown on Table 2.**

- - 1. **80TTreatment Duration**
       1. **80TPhase I**

**80TPatients will receive treatment for at least until week 8 as long as there are no DLTs in the first 4 weeks, or significant toxicities requiring treatment discontinuation in the judgment of the treating investigator, and no evidence of clinical progression. At week 8, if tumor assessment by scans show evidence or response by RECIST 1.1 criteria (applies only to patients with measurable disease), they will be allowed to stay on treatment as long as there is no evidence of progression or significant toxicities up to a maximum of 1 year. This maximum timeframe is due to concerns about HCQ toxicity, which could be duration-dependent. Patients who show progression or stability of disease at week 8 will come off study and can continue standard treatment (standard dose palbociclib and letrozole) or alternate therapy at the discretion of the treating physician.**

- - - 1. **80TPhase II**

**80TFollowing the study schema (Figure 2) patients are expected to complete80T** 28 days of study treatment followed by surgery for the “window option”, with an off study visit after surgery, and will be monitored for adverse events up to 30 days after the last dose of study drug.

Patients whose tumors at T1 biopsy show Ki67 > 10% will be required to come off study and per standard of care would typically be recommended to proceed to definitive surgery or standard neoadjuvant chemotherapy. All other patients will continue on study therapy and at T2 (week 5, or day 29), will proceed with definitive surgery or undergo a research biopsy and continue with standard of care neoadjuvant endocrine therapy or chemotherapy as clinically indicated.

- - 1. **80TEnd of treatment visit and Follow up assessments**

Patients who discontinue study treatment, will be followed for 30 days after stopping the study drugs for assessment of safety (i.e., assessment of AEs and/or serious AEs [SAEs] and concomitant medications) and resolution of any treatment related toxicity. Patients continuing to experience toxicity at this point following discontinuation of treatment will continue to be followed at least every 4 weeks until resolution or determination, in the clinical judgment of the investigator, that no further improvement is expected.

- - 1. **Early discontinuation**

Patients who come off trial early will be asked to return for the off-study assessments as detailed in Tables 1 and 2. In the event of a continuing AE, the patient will be asked to return for follow-up until resolution or stabilization of the AE.

- - 1. **Review of Systems and Physical Exam**

A full review of systems that covers all of the domains and systems represented in CTCAE

V 4.03 toxicity criteria will be performed at screening/baseline. Subsequent physical exams may be limited and should be focused on sites of disease to explore clinical signs and symptoms.

- - 1. **Laboratory Assessments**

CBC/differential, electrolytes, BUN, creatinine, glucose, albumin, AST, ALT, total bilirubin, alkaline phosphatase, PT, PTT, and Urinalysis.

- - 1. **80TImaging assessments Phase I**

**80TTumor response will be assessed according to RECIST Version 1.1 using standard contrast-enhanced computed tomographic scanning of the chest, abdomen and pelvis. In the case of iodine contrast allergy or reaction, either non-contrast-enhanced or MRI scanning can be performed. Patients should have at least one documented measurable lesion (per RECIST v1.1) or in the absence of measurable disease, have at least one lytic or mixed (blastic/lytic) bone lesion at study entry.**

**80TImaging assessments will be performed at screening within 28 days prior to first dose of study treatment, at 8 weeks (+/- 1 week) from day one of study treatment and subsequently every 8-12 weeks (+/- 2 weeks) thereafter.**

**80TTumor response should be assessed using the same imaging method throughout the study.**

- - 1. **Imaging assessments Phase II**

Methods for ultrasound evaluation and volumetric measure of response

Grayscale and power Doppler ultrasound will be performed on the breast and regional nodal basins using a Philips iU22 system or Sonoline Antares systems equipped with a 5- to 13-MHz broadband linear transducer. Ultrasound images of the index tumors and index nodes will be captured in the longitudinal and transverse planes with three dimensions measured. The size of the index tumors and index nodes will be reported in three dimensions to allow for volumetric calculation. The percent change in volume will then be calculated and reported to determine response.

- 1. **80TCorrelative Biomarker Studies**
     1. **80TBiopsies, Tissue Handling and Analysis**

All biopsies on Phase I are optional and will be done with appropriate image guidance. Biopsies for Phase II are mandatory and will be done under ultrasound guidance by a member of the research team using standard of care imaging-guided biopsy. Local anesthesia and hemostasis protocols will be used as per standard protocol. Core needles of 18 of 16G size will be used and 3-4 core biopsies will be obtained. The first core will be placed in standard 10% formalin, and all other cores will be placed in a cryovial and snap frozen in liquid nitrogen.

All of the correlative studies (unless otherwise indicated) will be performed in the laboratory of Dr. Khandan Keyomarsi located in the Zayed building at 6565 MD Anderson Blvd, Houston, Texas, 77030. For all the correlative analysis we will use tumor tissues collected from the biopsies and at surgical resections.

Cyclin E, and Rb immunohistochemistry (IHC) assays. FFPE tumor tissue slides will be prepared from paraffin imbedded blocks per standard methodologies and subjected to IHC analysis with 2 different antibodies: cyclin E and Rb:

Cyclin E: the anti-human C-19 polyclonal antibody (Santa Cruz Biologicals) at 1:2000 dilution will be used to stain each slide. Each slide will be scored for percent nuclear and percent cytoplasmic positive staining. Only tumor cells with greater than 5% nuclear or cytoplasmic positivity are considered as cyclin E positive. Nuclear and cytoplasmic staining scores are assigned according to the staining intensity (0 = no staining, 1 = blush staining, 2 = weak staining, 3 = intermediate staining and 4= strong staining). The nuclear and cytoplasmic scores are then combined, and four immunophenotypes are identified. First, breast tumors will be considered to be negative for cyclin E when staining is not detected in the nuclei or cytoplasm (phenotype 1). Second, in cases determined to be cyclin E+, if the nuclear staining score exceeded the cytoplasmic score, cyclin E expression is defined as predominantly nuclear (phenotype 2). Third, when the nuclear and cytoplasmic staining scores are equal, cyclin E expression is considered to be both nuclear and cytoplasmic (phenotype 3). Fourth, if the cytoplasmic staining score is higher than the nuclear score, cyclin E expression is considered to be predominantly cytoplasmic (phenotype 4).

Rb: The anti-human Clone 4H1 mouse monoclonal antibody (Cell Signaling Technology, Denvers, MA) at 1:100 dilution will be used to stain each slide. Each slide will be scored separately for intensity of staining and percentage of positive cells. Staining intensity will be scored as follows: 0, no staining; 1, weak positive (faint yellow staining); 2, intermediate positive; and 3, strong positive (brown staining). The number of positive cells will visually be evaluated and stratified as follows: <1%, 0 (negative); 1 to <5% positive cells, 1 (weak); 5-50% positive cells, 2 (moderate); >50% positive cells, 3 (strong). The sum of the staining intensity and percentage of positive cell scores will be used to determine the staining index for each section, with a minimum score of 0 and maximum score of 6; scores >1 will be defined as Rb positivity.

- - 1. **80TWhole Blood/Plasma Biospecimens**

**80TBlood specimens (Phase II only) will be collected as shown on Table 2 at baseline, days 15 and 29 for patients on the “window” option and for those who stay on study until surgery will be collected on days 43, 57 and every 4 weeks thereafter until surgery. Each blood draw will be to collect three tubes – one for plasma (8 mL CPT top) and the other for serum (6 mLred top) and one for whole blood (6 mLlavender). Blood will be processed immediately following collections – tubes will be gently inverted and kept at room temperature prior to centrifuging, and clotted tube (red top) will have serum aspirated, while the green top tube will have plasma aspirated – both being aliquoted in 0.5 mL cryovials and placed in a -20⁰C freezer (and stored long term at -80⁰C). A laboratory protocol contains additional details.**

- - 1. **Ki67 Tumor Assay and Scoring**

CLIA-approved Ki67 staining will be performed using anti-Ki67 rabbit monoclonal antibody (isotype IgG1κ, clone MIB-1, DAKO) in the diagnostic Immunohistochemistry Laboratory of Department of Pathology using an established protocol. Briefly this protocol includes de-paraffinization (30 minutes at 72°C)and rehydration with antigen retrieval performed at 100°C for 20 minutes with Tris-EDTA buffer, pH 6.0.

Endogenous peroxidase is blocked with 3% peroxide for 5 minutes. Primary anti-Ki-67 antibody (Dako, clone MIB-1) is applied at 1:100 dilution for 15 minutes. Post primary antibody detection is carried out using a commercial polymer system (Bond Polymer Refine Detection, Leica), and stain development is achieved by incubation with DAB and DAB Enhancer (Leica).

Ki67 staining will be evaluated in whole tissue sections without focusing on the hot spots. The staining will be quantitatively assessed by automated image analysis using the Aperio ScanScope AT2 scanner (Leica Biosystems, Inc., 1700 Leider Lane, Buffalo Grove IL, 60089).  All Ki67 immunostained slides will be scanned at 20 X magnification.  As Ki67 is a nuclear stain, the Genie nuclear v9.1 algorithm will be used to create a custom-made classifier.  A color graphic phase of image analysis is afterward performed using red, orange, and yellow  (high, medium, and low reaction, respectively) to represent positive cells, and blue to represent negative ones.  A curvature threshold adjustment is made to de-cluster or break up large groups of closely apposed nuclei when needed. The original factory algorithm is also adjusted to avoid false positives by lowering the Cytoplasmic Intensity settings. All algorithm adjustments are tested to assure accurate detection of positive and negative nuclei.

- 1. **80TBanked Biospecimens**

Leftover blood and tumor samples which are not consumed for planned study testing will be retained for potential additional testing at a later date (i.e. if newer technologies become available) under the same objectives. Samples will be retained at a secure storage facility (in the laboratory of Dr. Keyomarsi located in the Zayed building at 6565 MD Anderson Blvd, Houston, Texas, 77030) in case there is need for retesting or additional testing. Samples will be stored for up to 10 years or until termination of this study. These may be used for future studies only as approved by the MD Anderson IRB and the Principal Investigator.

1. **80T6.INVESTIGATIONAL AND NON – INVESTIGATIONAL AGENTS**

   2. **80TPalbociclib**
      1. **Description of palbociclib**

Palbociclib is an inhibitor of cyclin-dependent kinase (CDK) 4 and 6, which are downstream of signaling pathways that lead to cellular proliferation. In vitro, palbociclib reduces cellular proliferation of ER-positive breast cancer cell lines by blocking progression of the cell from G1 into S phase of the cell cycle. Treatment of breast cancer cell lines with the combination of palbociclib and endocrine therapies leads to decreased retinoblastoma protein (Rb) phosphorylation resulting in reduced E2F expression and signaling, and increased growth arrest compared to treatment with each drug alone. In vitro treatment of ER-positive breast cancer cell lines with the combination of palbociclib and antiestrogens leads to increased cell senescence, which was sustained for up to 6 days following drug removal. In vivo studies using a patient-derived ER-positive breast cancer xenograft model demonstrated that the combination of palbociclib and letrozole increased the inhibition of Rb phosphorylation, downstream signaling and tumor growth compared to each drug alone (Pfizer Pharma United States product insert (USPI), (2015). Highlights of Prescribing Information; IBRANCE® (palbociclib) for oral use. New York: Pfizer, Inc. – details are contained in the package insert in Appendix A).

Complete information for palbociclib may be found in the single reference safety document (SRSD), which for this study is the Pfizer Investigator Brochure for Palbociclib (PD-0332991).

- - 1. **Source of palbociclib**

Palbociclib will be provided by Pfizer Inc. Palbociclib commercial supply will be supplied as 75 mg capsules in High Density Polyethylene (HDPE) bottles, labeled according to investigational pharmacy procedures.

- - 1. **Preparation and dispensing**

Qualified site personnel will provide adequate palbociclib supplies for patient to take home until next scheduled visit.

Patients will receive a drug diary to document dosing. The completed diary must be returned to the site at the next study visit.

- - 1. **Dosing of palbociclib and dose adjustments for palbociclib (holding, discontinuation)**

Patients should be instructed to swallow palbociclib capsules whole and not to manipulate or chew them prior to swallowing. No capsule should be ingested if it is broken, cracked, or otherwise not intact. Patients should be encouraged to take their dose at approximately the same time each day. Patients should be instructed to record daily administration in the patient diary.

Patients should take palbociclib with food.

- Patients who miss a day’s dose entirely must be instructed NOT to “make it up” the next day.
- Patients who vomit any time after taking a dose must be instructed NOT to “make it up,” and to resume treatment the next day as prescribed.
- Patients who inadvertently take 1 extra dose during a day must be instructed to skip the next day’s dose.

Dose adjustments and patient discontinuation criteria are as follows:

- Palbociclib will be held for grade 3 or 4 neutropenia, and CBC/diff will be checked in one week intervals, with treatment resumed at the same dose when neutropenia has recovered to Grade ≤2. If more than two weeks are required for recovery to Grade ≤2, the dose will be lowered to 75 mg by mouth twice a week.
- Once palbociclib dose has been lowered, it will not be re-escalated. If criteria for another dose reduction are met, the patient must be permanently discontinued
- Any episode of febrile neutropenia (fever ≥38.5°C) with ANC <1000 will require discontinuation
- For Grade ≥3 non-hematologic toxicity attributed to palbociclib, palbociclib will be held until the toxicity in question resolves to Grade ≤1 or Grade ≤2 if not considered a risk to the patient.
- Any patient with liver function test abnormalities meeting Hy’s Law criteria must be permanently discontinued
  1. **80THydroxychloroquine**
     1. Description of hydroxychloroquine (HCQ):

Hydroxychloroquine (HCQ) is a small molecule drug that is indicated for the suppressive treatment and treatment of acute attacks of malaria due to *Plasmodium vivax*, *P. malariae*, *P. ovale*, and susceptible strains of *P. falciparum*. It is also indicated for the treatment of discoid and systemic lupus erythematosus, and rheumatoid arthritis, and often used for overlap connective tissue disorders. Although the exact mechanism of action is unknown, it may be based on ability of HCQ to bind to and alter DNA. HCQ has also has been found to be taken up into the acidic food vacuoles of the parasite in the erythrocyte. This increases the pH of the acid vesicles, interfering with vesicle functions and possibly inhibiting phospholipid metabolism. In suppressive treatment, HCQ inhibits the erythrocytic stage of development of plasmodia. In acute attacks of malaria, it interrupts erythrocytic schizogony of the parasite. Its ability to concentrate in parasitized erythrocytes may account for their selective toxicity against the erythrocytic stages of plasmodia infection. As an anti-rheumatic, HCQ is thought to act as a mild immunosuppressant, inhibiting the production of rheumatoid factor and acute phase reactants. It also accumulates in white blood cells, stabilizing lysosomal membranes and inhibiting the activity of many enzymes, including collagenase and the proteases that cause cartilage breakdown.

HCQ is also known to inhibit autophagy, a cellular process that may also lead to resistance to several cancer drugs, and as such, several trials have been conducted and shown safety of HCQ combined with several antineoplastic agents.P^34-37^P However, no trials have tested HCQ in this setting and with the rationale and drug combination proposed in this trial. Additional detail is provided in the package insert in Appendix B.

- - 1. **Source and dispensing of hydroxychloroquine**

Generic HCQ as 200 mg tablets will be obtained from a commercial source, Quality Prescription Drugs (Suite #245, 7360 137th Street, Surrey, and B.C. V3W), a Canada-based company that uses U.S. sources that is both CIPA certified and Pharmacy Checker approved. The drug will be ordered for direct delivery to the Investigational Pharmacy at MD Anderson in batches to maintain at least a 3 months of supply on hand and will be dispensed as an investigational drug with standard logging of drug acquisition, dispensation and final accounting for disposition. Unused or returned drug will be destroyed. Drug will be dispensed to patients with the name and dosage drug, date dispensed, name of patients, name of study and instructions for use.

- - 1. **Dosing of hydroxychloroquine and dose adjustments for hydroxychloroquine**

HCQ will be given by mouth at the assigned dose for the Phase I dose escalation and Phase II fixed dose parts of the trial The dose of 400 mg daily was chosen as the starting dose for the Phase I portion as this is the usual dose used clinically and has been combined with endocrine and other and cancer therapies at this does in prior studies.P^34-37^P There will be no dose adjustments for HCQ, and any grade 3 toxicity that is attributed to HCQ will require discontinuation of drug and for the patient to come off study. Subsequent treatment will be as per standard of care. For patients on the Phase I portion, any Grade III toxicity attributed to HCQ will also be counted as a dose-limiting toxicity if occurring in the first 28 days.

- 1. **Letrozole**

Letrozole at the standard approved dose of 2.5 mg by mouth daily continuous dosing will be prescribed as standard of care for neoadjuvant endocrine therapy through MD Anderson’s or the patient’s choice of pharmacy. There will be no dose adjustments for letrozole. If the patient is felt to be having unacceptable toxicities due to letrozole, then treatment will be stopped and the patient will come off study. Subsequent treatment will be as per standard of care. Additional details are provided in the package insert in Appendix C.

- 1. **Concomitant Medications**

In general, the use of any concomitant medication or therapies deemed necessary for the care of the patient is permitted with the exception of strong CYP3A inhibitors or inducers.

1. **ADVERSE EVENT MONITORING AND REPORTING**

   2. **Definition of an Adverse Event**

**An adverse event is the appearance or worsening of any undesirable sign, symptom, or medical condition occurring after starting the study drug even if the event is not considered to be related to study drug. Medical conditions/diseases present before starting study drug are only considered adverse events if they worsen after starting study drug. Abnormal laboratory values or test results constitute adverse events only if they induce clinical signs or symptoms, are considered clinically significant, or require therapy.**

~~
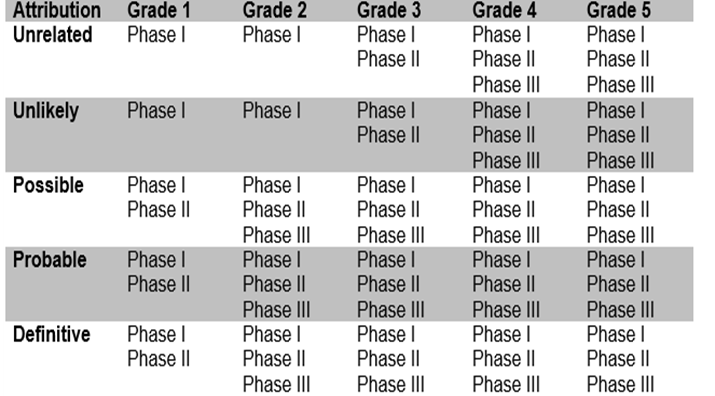
~~

**7.2 Serious Adverse Event (SAE) Reporting**

An adverse event or suspected adverse reaction is considered “serious” if, in the view of either the investigator or the sponsor, it results in any of the following outcomes:

- Death
- A life-threatening adverse drug experience – any adverse experience that places the patient, in the view of the initial reporter, at immediate risk of death from the adverse experience as it occurred. It does not include an adverse experience that, had it occurred in a more severe form, might have caused death.
- Inpatient hospitalization or prolongation of existing hospitalization
- A persistent or significant incapacity or substantial disruption of the ability to conduct normal life functions.
- A congenital anomaly/birth defect.
- Hy’s Law

Important medical events that may not result in death, be life-threatening, or require hospitalization may be considered a serious adverse drug experience when, based upon appropriate medical judgment, they may jeopardize the patient or subject and may require medical or surgical intervention to prevent one of the outcomes listed in this definition. Examples of such medical events include allergic bronchospasm requiring intensive treatment in an emergency room or at home, blood dyscrasias or convulsions that do not result in inpatient hospitalization, or the development of drug dependency or drug abuse (21 CFR 312.32).

- Important medical events as defined above, may also be considered serious adverse events. Any important medical event can and should be reported as an SAE if deemed appropriate by the Principal Investigator or the IND Sponsor, IND Office.
- All events occurring during the conduct of a protocol and meeting the definition of a SAE must be reported to the IRB in accordance with the timeframes and procedures outlined in “The University of Texas M. D. Anderson Cancer Center Institutional Review Board Policy for Investigators on Reporting Serious Unanticipated Adverse Events for Drugs and Devices”. Unless stated otherwise in the protocol, all SAEs, expected or unexpected, must be reported to the IND Office, regardless of attribution (within 5 working days of knowledge of the event).
- **All life-threatening or fatal events,** that are unexpected, and related to the study drug, must have a written report submitted within **24** **hours** (next working day) of knowledge of the event to the Safety Project Manager in the IND Office.
- Unless otherwise noted, the electronic SAE application (eSAE) will be utilized for safety reporting to the IND Office and MDACC IRB.
- Serious adverse events will be captured from the time of the first protocol-specific intervention, until 30 days after the last dose of drug, unless the participant withdraws consent. Serious adverse events must be followed until clinical recovery is complete and laboratory tests have returned to baseline, progression of the event has stabilized, or there has been acceptable resolution of the event.
- Additionally, any serious adverse events that occur after the 30 day time period that are related to the study treatment must be reported to the IND Office. This may include the development of a secondary malignancy.

**Reporting to FDA:**

- Serious adverse events will be forwarded to FDA by the IND Sponsor (Safety Project Manager IND Office) according to 21 CFR 312.32.

**It is the responsibility of the PI and the research team to ensure serious adverse events are reported according to the Code of Federal Regulations, Good Clinical Practices, the protocol guidelines, the sponsor’s guidelines, and Institutional Review Board policy.**

**Adverse Event Reporting Period**

The study period during which all AEs and SAEs must be reported begins after informed consent is obtained and starting protocol intervention and ends 30 days following the last administration of study treatment or study discontinuation/termination, whichever is earlier.

After this period, only SAEs that are attributed to prior study treatment will be reported. All AEs, regardless of attribution, will be collected during Phase I and II studies for at least 30 days after last dose of the study drugs.

- 1. **Recording of Adverse Events**

Medical events that may not result in death, be life-threatening, or require hospitalization may be considered a serious adverse drug experience when, based upon appropriate medical judgment, they may jeopardize the patient or subject and may require medical or surgical intervention to prevent one of the outcomes listed in this definition. Examples of such medical events include allergic bronchospasm requiring intensive treatment in an emergency room or at home, blood dyscrasias or convulsions that do not result in inpatient hospitalization, or the development of drug dependency or drug abuse (21 CFR 312.32).

- Important medical events as defined above, may also be considered serious adverse events.
- Any important medical event can and should be reported as an SAE if deemed appropriate by the Principal Investigator. All events occurring during the conduct of a protocol and meeting the definition of a SAE must be reported to the IRB in accordance with the timeframes and procedures outlined in “The University of Texas M. D. Anderson Cancer Center Institutional Review Board Policy for Investigators on Reporting Serious Unanticipated Adverse Events for Drugs and Devices”. Unless stated otherwise in the protocol, all SAEs, expected or unexpected, must be reported to the IND Office, regardless of attribution (within 5 working days of knowledge of the event).

Serious adverse events will be captured in the RedCap electronic system from the time of the first protocol specific intervention, until 30 days after the last dose of drug, unless the participant withdraws consent. Serious adverse events must be followed until clinical recovery is complete and laboratory tests have returned to baseline, progression of the event has stabilized, or there has been acceptable resolution of the event. It is the responsibility of the PI and the research team to ensure serious adverse events are reported according to the Code of Federal Regulations, Good Clinical Practices, the protocol guidelines, the sponsor’s guidelines, and Institutional Review Board policy.

This study will be performed in compliance with the principles of International Conference on Harmonization (ICH) Good Clinical Practices (GCP) Guideline.

- 1. **80TAssessment of Adverse Events**

The PI or designee will be responsible for assigning attribution of adverse events to the study agent. As far as possible, each adverse event should be evaluated using CTCAE V 4.03 criteria to determine:

- - - The severity grade (mild, moderate, severe) or grade (1-4).
    - Its relationship to the study drug(s) (suspected/not suspected).
    - Its duration (start and end dates or if continuing at the final exam).
    - Action taken (no action taken; study drug dosage adjusted/temporarily interrupted; study drug permanently discontinued due to this adverse event; concomitant medications taken; non-drug therapy given; hospitalization/prolonged hospitalization.
    - Whether it constitutes a serious adverse events (SAE).

All AEs and SAEs, whether volunteered by the patient, discovered by study personnel during questioning, or detected through physical examination, laboratory test, or other means, will be reported appropriately.

Expected AEs are those AEs that are listed or characterized in the Package Insert (PI) or current Investigator’s Brochure.

Unexpected AEs are those not listed in the PI or current Investigator’s Brochure or not identified. This includes AEs for which the specificity or severity is not consistent with the description in the PI or Investigator’s Brochure. For example, under this definition, hepatic necrosis would be unexpected if the PI or Investigator’s Brochure only referred to elevated hepatic enzymes or hepatitis.

The investigator (or physician designee) is responsible for verifying and providing source documentation for all adverse events and assigning the attribution for all adverse events for subjects enrolled.

- 1. **80TCommunications between the Investigator and Supporting Company Pfizer**

SAEs that occur after completion of the reporting time period as defined above are reportable to Pfizer if the Investigator suspects a causal relationship between the Pfizer product and the SAE. Of note, Pfizer is supporting the study only with provision of palbociclib.

In addition, all SAEs must also be reported to Pfizer within 24 hours, directed to “Pfizer U.S. Clinical Trial Department” using institutional MD Anderson SAE forms to FAX number 1-866-997-8322 (See Appendix G in PDOL for Pfizer Reportable Events Fax Form).

1. **80T** **STATISTICAL CONSIDERATIONS**

**Phase I:**

For the phase I portion, a 3+3 design will be used with 3 to 6 patients for each dose escalation cohort (depending on whether a DLT is seen) and 6 patients in the final (RP2D) cohort to assess the primary endpoint of safety and the determination of RP2D.^38^ Therefore a minimum of 12 and maximum of 18 patients will be enrolled. This total does not include patients who may need to be replaced if not evaluable for DLT (see Section 3.3).

**Phase II:**

The Phase II portion will be performed in a different population of patients (early stage) in the pre-operative setting. In order to obtain qualitative information about the dose responsiveness of HCQ when added to letrozole and palbociclib, Part 1 of the Phase II portion will enroll 6 patients at an HCQ dose of 400 mg (if in fact, the RP2D is 600 or 800 mg). Following this, Part 2 will be conducted at the RP2D for HCQ based on the primary endpoint of proportion of patients achieving tumoral CCCA, with the formal statistical plan using a 2-stage Simon optimal design and looking to increase the CCCA rate from 50% with low dose palbociclib + letrozole to 75% after the addition of HCQ. This design requires 11 patients in the first stage and 14 patients in the 2nd stage. It would stop after the 1st stage if no more than 6 patients show CCCA by tumor biopsy after HCQ treatment and declared the treatment as promising if at least 17 out of 25 total evaluable patients exhibit CCCA.

This design has alpha = 4%, 81% power, and a probability of 0.73 of terminating after the 1st stage if the true CCCA rate=50%. For the group of patients at the RP2D, we therefore plan a sample size of 30 patients over 12-15 months, accounting for patient dropout (including those with Ki67 >10% at T1). We have a large eligible patient population and have led national accruals in other multi-center neoadjuvant endocrine trials in post-menopausal women (e.g. ALTERNATE Trial). In addition, recent local experience of conducting window of opportunity trials in this setting have demonstrated the feasibility of accrual at a rate of ~2 patients per month. Thus, we expect to achieve accrual goals.

**8.1 IND Office Reporting**

The Investigator is responsible for completing toxicity/efficacy summary reports and submitting them to the IND office

Medical Affairs and Safety Group for review. These should be submitted as follows:

- Phase I:

After the first 3 evaluable patients, complete 1 cycle of study treatment, and every 3 evaluable patients thereafter, IND Office approval must be obtained prior to advancing/changing dose levels.

- Phase II:

After the first 6 evaluable patients complete 4 weeks of study treatment, and every 6 patients thereafter.

A copy of the cohort summary should be placed in the Investigator’s Regulatory Binder under “sponsor correspondence”.

1. **80T** **INSTITUTIONAL REVIEW, CONSENT, MEDICAL MONITORING AND RECORD-KEEPING AND SAFETY MONITORING**

## **9.1** **Informed Consent**

The informed consent documents must be signed by the subject or the subject’s legally authorized representative before his or her participation in the study. The case history for each subject shall document that informed consent was obtained prior to participation in the study. A copy of the informed consent document must be provided to the subject or the subject's legally authorized representative. If applicable, it will be provided in a certified translation of the local language.

Signed consent forms must remain in each subject’s study file and must be available for verification by study monitors at any time.

##

**9.2 Institutional Review Board Approval**

This protocol, the informed consent document, and relevant supporting information must be submitted to the IRB for review and must be approved before the study is initiated. The study will be conducted in accordance with FDA, applicable national and local health authorities, and IRB requirements.

The Principal Investigator is responsible for keeping the IRB apprised of the progress of the study and of any changes made to the protocol as deemed appropriate, but in any case, the IRB must be updated at least once a year. The Principal Investigator must also keep the IRB informed of any significant AEs.

Investigators are required to promptly notify their respective IRB of all adverse drug reactions that are both serious and unexpected. This generally refers to SAEs that are not already identified in the Investigator’s Brochure and that are considered possibly or probably related to the molecule or study drug by the investigator. Some IRBs may have other specific AE requirements to which investigators are expected to adhere. Investigators must immediately forward to their IRB any written safety report or update provided by Pfizer (e.g., IND safety report, Investigator’s Brochure, safety amendments and updates, etc.).

## **Confidentiality**

Patient medical information obtained by this study is confidential and may be disclosed to third parties only as permitted by the ICF (or separate authorization to use and disclose personal health information) signed by the patient or unless permitted or required by law. Medical information may be given to a patient’s personal physician or other appropriate medical personnel responsible for the patient’s welfare for treatment purposes. Data generated by this study must be available for inspection upon request by representatives of the FDA and other regulatory agencies as applicable.

# Study Medical Monitoring Requirements

This clinical research study will be monitored internally (by the PI) and externally (by the MDACC IRB). In terms of internal review, the PI will continuously monitor and tabulate AEs. Appropriate reporting to the MDACC IRB will be made. The PI of this study will also continuously monitor the conduct, data, and safety of this study to ensure that:

- Interim and safety analyses occur as scheduled,
- Stopping rules for toxicity and/or response are met,
- Risk/benefit ratio is not altered to the detriment of the subjects,
- Appropriate internal monitoring of AEs and outcomes is done,
- Over-accrual does not occur,
- Under-accrual is addressed with appropriate amendments or actions, and
- Data are being appropriately collected in a reasonably timely manner.

Routine monitoring will be carried out via a periodic team conference among investigators during which toxicity data, including all SAEs, will be reviewed and other issues relevant to the study such as interim assessment of accrual, outcome, and compliance with study guidelines, will be discussed. Monitoring will be carried out on an ongoing basis. The severity, relatedness, and whether or not the event is expected will be reviewed. Data will be housed and analyzed on a Redcap database using our institutional and Departmental protocols for data entry and verification.

## **Data Collection**

The study coordinator and investigators are responsible for ensuring that the eligibility checklist is completed in a legible and timely manner for every patient enrolled in the study, and that data are recorded on the appropriate forms and in a timely manner. Any errors on source data should be lined through, but not obliterated, with the correction inserted, initialed, and dated by the study coordinator or PI. All source documents will be available for inspection by the FDA and the MD Anderson IRB.

**REFERENCES**

1. Hammond ME, Hayes DF, Dowsett M, et al. American Society of Clinical Oncology/College Of American Pathologists guideline recommendations for immunohistochemical testing of estrogen and progesterone receptors in breast cancer. J Clin Oncol. 28:2784-95, 2010.
2. Wolff AC, Hammond MEH, Hicks DG, et al. Recommendations for human epidermal growth

factor receptor 2 testing in breast cancer: American Society of Clinical Oncology/College of American Pathologists clinical practice guideline update. J Clin Oncol. 31:3997-4013, 2013.

1. Siegel RL, Miller KD, Jemal A. Cancer Statistics, 2017. CA Cancer J Clin. 67:7-30, 2017.
2. Howlader N, Altekruse SF, Li CI, et al. US incidence of breast cancer subtypes defined by joint hormone receptor and HER2 status. J Natl Cancer Inst. 106:ii, 2014.
3. Early Breast Cancer Trialists' Collaborative Group (EBCTCG), Relevance of breast cancer hormone receptors and other factors to the efficacy of adjuvant tamoxifen: patient-level meta-analysis of randomized trials. Lancet. 378:771-84, 2011.
4. Mehta R, Barlow W, Albain K, et al. Combination anastrozole and fulvestrant in metastatic breast cancer. N Engl J Med. 367: 435–44, 2012.
5. Bergh J, Johnson P, Lidbrink E, et al. Fact: an open-label randomized phase III study of fulvestrant and anastrozole in combination compared with anastrozole alone as first-line therapy for patients with receptor-positive postmenopausal breast cancer. J Clin Oncol 30: 1919–25, 2012.
6. Baselga J, Campone M, Piccart, M, et al. Everolimus in postmenopausal hormone-receptor-positive advanced breast cancer. N Engl J Med 366: 520–29, 2012.
7. Piccart M, Hortobagyi GN, Campone M, et al. Everolimus plus exemestane for hormone-receptor-positive, human epidermal growth factor receptor-2-negative advanced breast cancer: overall survival results from BOLERO-2. Ann Oncol. 25:2357-62, 2014.
8. Rugo HS, Rumble RB, Macrae E, et al. endocrine therapy for hormone receptor-positive metastatic breast cancer: American Society of Clinical Oncology Guideline. J Clin Oncol. 34:3069-103, 2016.
9. Finn RS, Dering J, Conklin D, et al. PD 0332991, a selective cyclin D kinase 4/6 inhibitor, preferentially inhibits proliferation of luminal estrogen receptor-positive human breast cancer cell lines in vitro. Breast Cancer Res. 11:R77, 2009.
10. Finn R, Hurvitz S, Allison M, et al. Phase I study of PD 0332991, a novel, oral, cyclin-D kinase (CDK) 4/6 inhibitor in combination with letrozole, for first-line treatment of metastatic post-menopausal, estrogen receptor-positive (ER+), human epidermal growth factor receptor 2 (HER2)-negative breast cancer. Cancer Res. 69 (24 suppl)**:** abstr 5069, 2009.
11. DeMichele A, Clark AS, Tan KS, et al. CDK 4/6 inhibitor palbociclib (PD0332991) in Rb+ advanced breast cancer: phase II activity, safety, and predictive biomarker assessment. Clin Cancer Res. 21:995-1001, 2015.
12. Finn RS, Crown JP, Lang I, et al. The cyclin-dependent kinase 4/6 inhibitor palbociclib in combination with letrozole versus letrozole alone as first-line treatment of oestrogen receptor-positive, HER2-negative, advanced breast cancer (PALOMA-1/TRIO-18): a randomized phase 2 study. Lancet Oncol. 16:25-35, 2015.
13. Finn RS, Martin M, Rugo HS, et al. Palbociclib and letrozole in advanced breast Cancer. N Engl J Med. 375:1925-36, 2016.
14. Turner NC, Ro J, André F, et al. Palbociclib in Hormone-Receptor-Positive Advanced Breast Cancer. N Engl J Med. 373:209-19, 2015.
15. Cristofanilli M, Turner NC, Bondarenko I, et al. Fulvestrant plus palbociclib versus fulvestrant plus placebo for treatment of hormone-receptor-positive, HER2-negative metastatic breast cancer that progressed on previous endocrine therapy (PALOMA-3): final analysis of the multicentre, double-blind, phase 3 randomized controlled trial. Lancet Oncol. 17:425-39, 2016.
16. Ma CX, Gao F, Northfelt D, et al. A Phase II trial of neoadjuvant palbociclib, a cyclin-dependent kinase (CDK) 4/6 inhibitor, in combination with anastrozole for clinical stage 2 or 3 estrogen receptor positive HER2 negative (ER+HER2-) breast cancer (BC). Cancer Res 76, Abstract S6-05, 2016. Also Ma CX, Gao F, Luo J, et al. NeoPalAna: Neoadjuvant Palbociclib, a Cyclin-Dependent Kinase 4/6 Inhibitor, and Anastrozole for Clinical Stage 2 or 3 Estrogen Receptor-Positive Breast Cancer. 35T[UClin Cancer Res.](https://www.ncbi.nlm.nih.gov/pubmed/?term=ma+cx+palbociclib)U0T35T 0T2017 Mar 7. doi: 10.1158/1078-0432.CCR-16-3206 (In Press).
17. Turner, NC*,* Jiang Y, O’Leary B, et al. Efficacy of palbociclib plus fulvestrant (P+F) in patients with metastatic breast cancer and ESR1 mutations in circulating tumor DNA. J Clin Oncol**.** 34 (suppl; abstr 512), 2016.
18. Turner NC, Neven P, Loibl S, et al. Advances in the treatment of advanced oestrogen-receptor-positive breast cancer. Lancet. 2016 Dec 6. pii: S0140-6736(16)32419-9, 2016. [Epub ahead of print]
19. Spring LM, Gupta A, Reynolds KL, et al. Neoadjuvant endocrine therapy for estrogen receptor-positive breast cancer: A systematic review and meta-analysis. JAMA Oncol. 2:1477-86, 2016.
20. DeMichele A, Yee D, Berry DA, et al. The Neoadjuvant Model Is Still the Future for Drug Development in Breast Cancer. Clin Cancer Res. 21:2911-5, 2015.
21. Dowsett M, Ebbs SR, Dixon JM, et al. Biomarker changes during neoadjuvant anastrozole, tamoxifen, or the combination: influence of hormonal status and HER-2 in breast cancer-a study from the IMPACT trialists. J Clin Oncol 2005; 23:2477-92.
22. Dowsett M, Smith IE, Ebbs SR, et al. Short-term changes in Ki67 during neoadjuvant treatment of primary breast cancer with anastrozole or tamoxifen alone or combined correlate with recurrence-free survival. Clin Cancer Res. 11:951-8s, 2005.
23. Dowsett M, Smith IE, Ebbs SR, et al. Proliferation and apoptosis as markers of benefit in neoadjuvant endocrine therapy of breast cancer. Clin Cancer Res. 12:1024-30s, 2006.
24. Dowsett M, Smith IE, Ebbs SR, et al. Prognostic value of Ki67 expression after short-term presurgical endocrine therapy for primary breast cancer. J Natl Cancer Inst. 99:167-70, 2007.
25. Ellis MJ, Tao Y, Luo J, et al. Outcome prediction for estrogen receptor-positive breast cancer based on post neoadjuvant endocrine therapy tumor characteristics. Natl Cancer Inst. 100:1380-8, 2008.
26. Jones RL, Salter J, A'Hern R, et al. The prognostic significance of Ki67 before and after neoadjuvant chemotherapy in breast cancer. Breast Cancer Res Treat. 116:53-68, 2009.
27. Polychronis A, Sinnett HD, Hadjiminas D, et al. Preoperative gefitinib versus gefitinib and anastrozole in postmenopausal patients with oestrogen-receptor positive and epidermal-growth-factor-receptor positive primary breast cancer: a double-blind placebo-controlled phase II randomized trial. Lancet Oncol. 6:383-91, 2005.
28. Baselga J, Semiglazov V, van Dam, et al. Phase II randomized study of neoadjuvant everolimus plus letrozole compared with placebo plus letrozole in patients with estrogen receptor-positive breast cancer. J Clin Oncol. 27:2630-7, 2009.
29. Smith IE, Walsh G, Skene A, et al. A phase II placebo-controlled trial of neoadjuvant anastrozole alone or with gefitinib in early breast cancer. J Clin Oncol. 25:3816-22, 2007.
30. Marmor MF, Kellner U, Lai TY, et al. Recommendations on screening for chloroquine and hydroxychloroquine retinopathy (2016 Revision). Ophthalmology. 123:1386-94, 2016
31. Pfizer Pharma. (2015). Highlights of prescribing information. IBRANCE (palbociclib). Retrieved from: http://www.Accessdata.fda.gov/drugsatfda_docs/label/2015/207103s000lbl.pdf. Accessed November 16, 2015
32. Mahalingam D, Mita M, Sarantopoulos J, et al. Combined autophagy and HDAC inhibition: a phase I safety, tolerability, pharmacokinetic, and pharmacodynamic analysis of hydroxychloroquine in combination with the HDAC inhibitor vorinostat in patients with advanced solid tumors. Autophagy. 10:1403-14, 2014.
33. Rangwala R, Chang YC, Hu J, et al. Combined MTOR and autophagy inhibition: phase I trial of hydroxychloroquine and temsirolimus in patients with advanced solid tumors and melanoma. Autophagy. 10:1391-1402, 2014.
34. Rangwala R, Leone R, Chang YC, et al. Phase I trial of hydroxychloroquine with dose-intense temozolomide in patients with advanced solid tumors and melanoma. Autophagy. 10:1369-79, 2014.
35. Rosenfeld MR, Ye X, Supko JG, et al. A phase I/II trial of hydroxychloroquine in conjunction with radiation therapy and concurrent and adjuvant temozolomide in patients with newly diagnosed glioblastoma multiform. Autophagy. 10:1359-68, 2014.
36. Le Tourneau C, Lee JJ, Siu LL. Dose escalation methods in Phase I cancer clinical trials. J Natl Cancer Inst. 101:708-20, 2009.

**APPENDIX A - Palbociclib (Ibrance) Highlights of Prescribing Information**

**HIGHLIGHTS OF PRESCRIBING INFORMATION**

**These highlights do not include all the information needed to use IBRANCE safely and effectively. See full prescribing information for IBRANCE.**

**IBRANCE® (palbociclib) capsules, for oral use Initial U.S. Approval: 2015**

**--------------------------- RECENT MAJOR CHANGES --------------------------­**

Indications and Usage (1) 03/2017

Dosage and Administration (2.1, 2.2) 03/2017

Warnings and Precautions (5.1, 5.2) 03/2017 Warnings and Precautions, Pulmonary Embolism (5) Removed 03/2017

**--------------------------- INDICATIONS AND USAGE---------------------------­**

IBRANCE is a kinase inhibitor indicated for the treatment of hormone receptor (HR)-positive, human epidermal growth factor receptor 2

(HER2)-negative advanced or metastatic breast cancer in combination with:

- an aromatase inhibitor as initial endocrine based therapy in postmenopausal women; or
- fulvestrant in women with disease progression following endocrine therapy. (1)

**-----------------------DOSAGE AND ADMINISTRATION ----------------------­**

IBRANCE capsules are taken orally with food in combination with an aromatase inhibitor or fulvestrant. (2)

- Recommended starting dose: 125 mg once daily taken with food for 21 days followed by 7 days off treatment. (2.1)
- Dosing interruption and/or dose reductions are recommended based on individual safety and tolerability. (2.2)

**--------------------- DOSAGE FORMS AND STRENGTHS---------------------­**

Capsules: 125 mg, 100 mg, and 75 mg. (3)

**------------------------------ CONTRAINDICATIONS -----------------------------­**

None. (4)

**----------------------- WARNINGS AND PRECAUTIONS ----------------------­**

- Neutropenia: Monitor complete blood count prior to start of IBRANCE therapy and at the beginning of each cycle, as well as on Day 15 of the first 2 cycles, and as clinically indicated. (2.2, 5.1)
- Embryo-Fetal Toxicity: IBRANCE can cause fetal harm. Advise patients of potential risk to a fetus and to use effective contraception. (5.2, 8.1, 8.3)

**------------------------------ ADVERSE REACTIONS -----------------------------­**

Most common adverse reactions (incidence ≥10%) were neutropenia, infections, leukopenia, fatigue, nausea, stomatitis, anemia, alopecia, diarrhea, thrombocytopenia, rash, vomiting, decreased appetite, asthenia, and

pyrexia. (6)

35T[**To report SUSPECTED ADVERSE REACTIONS, contact Pfizer Inc at 1-800-438-1985 or FDA at 1-800-FDA-1088 or *www.fda.gov/medwatch*.**](http://www.fda.gov/medwatch)

**------------------------------ DRUG INTERACTIONS------------------------------­**

- CYP3A Inhibitors: Avoid concurrent use of IBRANCE with strong CYP3A inhibitors. If the strong inhibitor cannot be avoided, reduce the IBRANCE dose. (2.2, 7.1)
- CYP3A Inducers: Avoid concurrent use of IBRANCE with strong CYP3A inducers. (7.2)
- CYP3A Substrates: The dose of sensitive CYP3A4 substrates with narrow therapeutic indices may need to be reduced when given concurrently with IBRANCE. (7.3)

----------------------------**USE IN SPECIFIC POPULATIONS**------------------­

- Lactation: Advise not to breastfeed. (8.2)

**See 17 for PATIENT COUNSELING INFORMATION and**

**FDA-approved patient labeling.**

**Revised: 03/312017**

**FULL PRESCRIBING INFORMATION: CONTENTS***

1. **INDICATIONS AND USAGE**
2. **DOSAGE AND ADMINISTRATION**
   1. Recommended Dose and Schedule
   2. Dose Modification
3. **DOSAGE FORMS AND STRENGTHS**
4. **CONTRAINDICATIONS**
5. **WARNINGS AND PRECAUTIONS**
   1. Neutropenia
   2. Embryo-Fetal Toxicity
6. **ADVERSE REACTIONS**
   1. Clinical Studies Experience
7. **DRUG INTERACTIONS**
   1. Agents That May Increase Palbociclib Plasma Concentrations
   2. Agents That May Decrease Palbociclib Plasma Concentrations
   3. Drugs That May Have Their Plasma Concentrations Altered by Palbociclib
8. **USE IN SPECIFIC POPULATIONS**
   1. Pregnancy
   2. Lactation
   3. Females and Males of Reproductive Potential
   4. Pediatric Use
   5. Geriatric Use
   6. Hepatic Impairment
   7. Renal Impairment
9. **OVERDOSAGE**
10. **DESCRIPTION**
11. **CLINICAL PHARMACOLOGY**
    1. Mechanism of Action
    2. Pharmacodynamics
    3. Pharmacokinetics
12. **NONCLINICAL TOXICOLOGY**
    1. Carcinogenesis, Mutagenesis, Impairment of Fertility
13. **CLINICAL STUDIES**
14. **HOW SUPPLIED/STORAGE AND HANDLING**
15. **PATIENT COUNSELING INFORMATION**
    - Sections or subsections omitted from the full prescribing information are not listed.


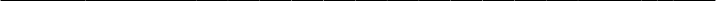


**FULL PRESCRIBING INFORMATION**

**1. INDICATIONS AND USAGE**

IBRANCE is indicated for the treatment of HR-positive, HER2-negative advanced or metastatic breast cancer in combination with:

- - - - an aromatase inhibitor as initial endocrine based therapy in postmenopausal women; or
      - fulvestrant in women with disease progression following endocrine therapy.
  - **DOSAGE AND ADMINISTRATION**
    1. **Recommended Dose and Schedule**

The recommended dose of IBRANCE is a 125 mg capsule taken orally once daily for 21 consecutive days followed by 7 days off treatment to comprise a complete cycle of 28 days. IBRANCE should be taken with food *[see Clinical Pharmacology (12.3)]*.

administer the recommended dose of an aromatase inhibitor when given with IBRANCE. Please refer to the Full Prescribing Information for the aromatase inhibitor being used.

When given with IBRANCE, the recommended dose of fulvestrant is 500 mg administered on Days 1, 15, 29, and once monthly thereafter. Please refer to the Full Prescribing Information of fulvestrant.

Patients should be encouraged to take their dose of IBRANCE at approximately the same time each day.

If the patient vomits or misses a dose, an additional dose should not be taken. The next prescribed dose should be taken at the usual time. IBRANCE capsules should be swallowed whole (do not chew, crush, or open them prior to swallowing). Capsules should not be ingested if they are broken, cracked, or otherwise not intact.

Pre/perimenopausal women treated with the combination IBRANCE plus fulvestrant therapy should be treated with luteinizing hormone-releasing hormone (LHRH) agonists according to current clinical practice standards.

- - 1. **Dose Modification**

The recommended dose modifications for adverse reactions are listed in Tables 1, 2, and 3.

1. **Table 1. Recommended Dose Modification for Adverse Reactions**

| **Dose Level** | **Dose** |
| --- | --- |
| Recommended starting dose | 125 mg/day |
| First dose reduction | 100 mg/day |
| Second dose reduction | 75 mg/day* |

If further dose reduction below 75 mg/day is required, discontinue.

| Monitor complete blood counts prior to the start of IBRANCE therapy and at the beginning of each cycle, as well as on Day 15 of the first 2 cycles, and as clinically indicated.  For patients who experience a maximum of Grade 1 or 2 neutropenia in the first 6 cycles, monitor complete blood counts for subsequent cycles every 3 months, prior to the beginning of a cycle and as clinically indicated. | |
| --- | --- |
| **CTCAE Grade** | **Dose Modifications** |
| Grade 1 or 2 | No dose adjustment is required. |
| Grade 3 | Day 1 of cycle:  Withhold IBRANCE, repeat complete blood count monitoring within 1 week. When recovered to Grade ≤2, start the next cycle at the *same dose*.  Day 15 of first 2 cycles:  If Grade 3 on Day 15, continue IBRANCE at current dose to complete cycle and repeat complete blood count on Day 22.  If Grade 4 on Day 22, see Grade 4 dose modification guidelines below.  Consider dose reduction in cases of prolonged (>1 week) recovery from Grade 3 neutropenia or recurrent Grade 3 neutropenia on Day 1 of subsequent cycles. |
| Grade 3 neutropenia with fever ≥38.5 ºC and/or infection | At any time:  Withhold IBRANCE until recovery to Grade ≤2. Resume at the *next lower dose*. |
| Grade 4 | At any time:  Withhold IBRANCE until recovery to Grade ≤2. Resume at the *next lower dose*. |

1. **Table 2. Dose Modification and Management – Hematologic Toxicities**

Grading according to CTCAE 4.0.

CTCAE=Common Terminology Criteria for Adverse Events; LLN=lower limit of normal.

a. Table applies to all hematologic adverse reactions except lymphopenia (unless associated with clinical events, e.g., opportunistic infections).

b. Absolute neutrophil count (ANC): Grade 1: ANC < LLN - 1500/mm3; Grade 2: ANC 1000 - <1500/mm3; Grade 3: ANC 500 - <1000/mm3; Grade 4: ANC <500/mm3.

1. **Table 3. Dose Modification and Management – Non-Hematologic Toxicities**

| **CTCAE Grade** | **Dose Modifications** |
| --- | --- |
| Grade 1 or 2 | No dose adjustment is required. |
| Grade ≥3 non-hematologic toxicity (if persisting despite optimal medical treatment) | Withhold until symptoms resolve to:  Resume at the *next lower dose*. |

- - - - Grade ≤1;
      - Grade ≤2 (if not considered a safety risk for the patient)

Grading according to CTCAE 4.0. CTCAE=Common Terminology Criteria for Adverse Events.

Refer to the Full Prescribing Information for coadministered endocrine therapy dose adjustment guidelines in the event of toxicity and other relevant safety information or contraindications.

1. **Dose Modifications for Use With Strong CYP3A Inhibitors**

Avoid concomitant use of strong CYP3A inhibitors and consider an alternative concomitant medication with no or minimal CYP3A inhibition. If patients must be coadministered a strong CYP3A inhibitor, reduce the IBRANCE dose to 75 mg once daily. If the strong inhibitor is discontinued, increase the IBRANCE dose (after 3 to 5 half-lives of the inhibitor) to the dose used prior to the initiation of the strong CYP3A inhibitor *[see Drug Interactions (7.1) and Clinical Pharmacology (12.3)].*

- - **DOSAGE FORMS AND STRENGTHS**

125 mg capsules: opaque, hard gelatin capsules, size 0, with caramel cap and body, printed with white ink “Pfizer” on the cap, “PBC 125” on the body.

100 mg capsules: opaque, hard gelatin capsules, size 1, with caramel cap and light orange body, printed with white ink “Pfizer” on the cap, “PBC 100” on the body.

75 mg capsules: opaque, hard gelatin capsules, size 2, with light orange cap and body, printed with white ink “Pfizer” on the cap, “PBC 75” on the body.

1. **CONTRAINDICATIONS**

None.

1. **WARNINGS AND PRECAUTIONS**
   1. **Neutropenia**

Neutropenia was the most frequently reported adverse reaction in Study 1 (PALOMA-2) with an incidence of 80% and Study 2 (PALOMA-3) with an incidence of 83%. A Grade ≥3 decrease in neutrophil counts was reported in 66% of patients receiving IBRANCE plus letrozole in Study 1 and 66% of patients receiving IBRANCE plus fulvestrant in Study 2. In Study 1 and 2, the median time to first episode of any grade neutropenia was 15 days and the median duration of Grade ≥3 neutropenia was 7 days *[see Adverse Reactions (6.1)]*.

Monitor complete blood counts prior to starting IBRANCE therapy and at the beginning of each cycle, as well as on Day 15 of the first 2 cycles, and as clinically indicated. Dose interruption, dose reduction, or delay in starting treatment cycles is recommended for patients who develop Grade 3 or 4 neutropenia *[see Dosage and Administration (2.2)]*.

Febrile neutropenia has been reported in 1.8% of patients exposed to IBRANCE across Studies 1 and 2. One death due to neutropenic sepsis was observed in Study 2. Physicians should inform patients to promptly report any episodes of fever *[see Patient Counseling Information (17)]*.

- 1. **Embryo-Fetal Toxicity**

Based on findings from animal studies and its mechanism of action, IBRANCE can cause fetal harm when administered to a pregnant woman. In animal reproduction studies, administration of palbociclib to pregnant rats and rabbits during organogenesis resulted in embryo-fetal toxicity at maternal exposures that were ≥4 times the human clinical exposure based on area under the curve (AUC). Advise pregnant women of the potential risk to a fetus. Advise females of reproductive potential to use effective contraception during treatment with IBRANCE and for at least 3 weeks after the last dose *[see Use in Specific Populations (8.1 and 8.3) and Clinical Pharmacology (12.1)]*.

**ADVERSE REACTIONS**

The following topic is described below and elsewhere in the labeling: Neutropenia *[see Warnings and Precautions (5.1)].*

- 1. **Clinical Studies Experience**

Because clinical trials are conducted under varying conditions, the adverse reaction rates observed cannot be directly compared to rates in other trials and may not reflect the rates observed in clinical practice.

**Study 1: IBRANCE plus Letrozole**

1. **Patients with estrogen receptor (ER)-positive, HER2-negative advanced or metastatic breast cancer for initial endocrine based therapy**

The safety of IBRANCE (125 mg/day) plus letrozole (2.5 mg/day) versus placebo plus letrozole was evaluated in Study 1 (PALOMA-2). The data described below reflect exposure to IBRANCE in 444 out of 666 patients with ER-positive, HER2-negative advanced breast cancer who received at least 1 dose of IBRANCE plus letrozole in Study 1. The median duration of treatment for IBRANCE plus letrozole was 19.8 months while the median duration of treatment for placebo plus letrozole arm was

13.8 months.

Dose reductions due to an adverse reaction of any grade occurred in 36% of patients receiving IBRANCE plus letrozole. No dose reduction was allowed for letrozole in Study 1.

Permanent discontinuation associated with an adverse reaction occurred in 43 of 444 (9.7%) patients receiving IBRANCE plus letrozole and in 13 of 222 (5.9%) patients receiving placebo plus letrozole. Adverse reactions leading to permanent discontinuation for patients receiving IBRANCE plus letrozole included neutropenia (1.1%) and alanine aminotransferase increase (0.7%).

The most common adverse reactions (≥10%) of any grade reported in patients in the IBRANCE plus letrozole arm by descending frequency were neutropenia, infections, leukopenia, fatigue, nausea, alopecia, stomatitis, diarrhea, anemia, rash, asthenia, thrombocytopenia, vomiting, decreased appetite, dry skin, pyrexia, and dysgeusia.

The most frequently reported Grade >3 adverse reactions (≥5%) in patients receiving IBRANCE plus letrozole by descending frequency were neutropenia, leukopenia, infections, and anemia.

Adverse reactions (≥10%) reported in patients who received IBRANCE plus letrozole or placebo plus letrozole in Study 1 are listed in Table 4.

1. **Table 4. Adverse Reactions (≥10%) in Study 1**

|  | **IBRANCE plus Letrozole (N=444)** | | | **Placebo plus Letrozole (N=222)** | | |
| --- | --- | --- | --- | --- | --- | --- |
| **Adverse Reaction** | **All Grades**  **%** | **Grade 3**  **%** | **Grade 4**  **%** | **All Grades**  **%** | **Grade 3**  **%** | **Grade 4**  **%** |
| Infections and infestations | | | |  | | |
| Infections | 60b | 6 | 1 | 42 | 3 | 0 |
| Blood and lymphatic system disorders | | | |  | | |
| Neutropenia Leukopenia Anemia Thrombocytopenia | 80  39  24  16 | 56  24  5  1 | 10  1  <1  <1 | 6  2  9  1 | 1  0  2  0 | 1  0  0  0 |
| Metabolism and nutrition disorders | | | |  | | |
| Decreased appetite | 15 | 1 | 0 | 9 | 0 | 0 |
| Nervous system disorders | | | |  | | |
| Dysgeusia | 10 | 0 | 0 | 5 | 0 | 0 |
| Gastrointestinal disorders | | | |  | | |
| Stomatitis  Nausea Diarrhea Vomiting | 30  35  26  16 | 1  <1  1  1 | 0  0  0  0 | 14  26  19  17 | 0  2  1  1 | 0  0  0  0 |
| Skin and subcutaneous tissue disorders | | | |  | | |
| Alopecia Rash Dry skin | 33d18  12 | N/A 1  0 | N/A 0  0 | 16e12  6 | N/A 1  0 | N/A 0  0 |
| General disorders and administration site conditions | | | |  | | |
| Fatigue Asthenia Pyrexia | 37  17  12 | 2  2  0 | 0  0  0 | 28  12  9 | 1  0  0 | 0  0  0 |

Grading according to CTCAE 4.0.

CTCAE=Common Terminology Criteria for Adverse Events; N=number of patients; N/A=not applicable;

a. Infections includes all reported preferred terms (PTs) that are part of the System Organ Class Infections and infestations.

b. Most common infections (>1%) include: nasopharyngitis, upper respiratory tract infection, urinary tract infection, oral herpes, sinusitis, rhinitis, bronchitis, influenza, pneumonia, gastroenteritis, conjunctivitis, herpes zoster, pharyngitis, cellulitis, cystitis, lower respiratory tract infection, tooth infection, gingivitis, skin infection, gastroenteritis viral, respiratory tract infection, respiratory tract infection viral, and folliculitis.

c. Stomatitis includes: aphthous stomatitis, cheilitis, glossitis, glossodynia, mouth ulceration, mucosal inflammation,

oral pain, oral discomfort, oropharyngeal pain, and stomatitis.

d. Grade 1 events – 30%; Grade 2 events – 3%.

e. Grade 1 events – 15%; Grade 2 events – 1%.

f. Rash includes the following PTs: rash, rash maculo-papular, rash pruritic, rash erythematous, rash papular, dermatitis, dermatitis acneiform, and toxic skin eruption.

Additional adverse reactions occurring at an overall incidence of <10.0% of patients receiving IBRANCE plus letrozole in Study 1 included alanine aminotransferase increased (9.9%), aspartate aminotransferase increased (9.7%), epistaxis (9.2%), lacrimation increased (5.6%), dry eye (4.1%), vision blurred (3.6%), and febrile neutropenia (2.5%).

1. **Table 5. Laboratory Abnormalities in Study 1**

|  | **IBRANCE plus Letrozole (N=444)** | | | **Placebo plus Letrozole (N=222)** | | |
| --- | --- | --- | --- | --- | --- | --- |
| **Laboratory Abnormality** | **All Grades**  **%** | **Grade 3**  **%** | **Grade 4**  **%** | **All Grades**  **%** | **Grade 3**  **%** | **Grade 4**  **%** |
| WBC decreased | 97 | 35 | 1 | 25 | 1 | 0 |
| Neutrophils decreased | 95 | 56 | 12 | 20 | 1 | 1 |
| Anemia | 78 | 6 | 0 | 42 | 2 | 0 |
| Platelets decreased | 63 | 1 | 1 | 14 | 0 | 0 |
| Aspartate aminotransferase increased | 52 | 3 | 0 | 34 | 1 | 0 |
| Alanine aminotransferase increased | 43 | 2 | <1 | 30 | 0 | 0 |

N=number of patients; WBC=white blood cells.

**Study 2: IBRANCE plus Fulvestrant**

1. **Patients with HR-positive, HER2-negative advanced or metastatic breast cancer who have had disease progression on or after prior adjuvant or metastatic endocrine therapy**

The safety of IBRANCE (125 mg/day) plus fulvestrant (500 mg) versus placebo plus fulvestrant was evaluated in Study 2 (PALOMA-3). The data described below reflect exposure to IBRANCE in 345 out of 517 patients with HR-positive, HER2-negative advanced or metastatic breast cancer who received at least 1 dose of IBRANCE plus fulvestrant in Study 2. The median duration of treatment for IBRANCE plus fulvestrant was 10.8 months while the median duration of treatment for placebo plus fulvestrant arm was 4.8 months.

Dose reductions due to an adverse reaction of any grade occurred in 36% of patients receiving IBRANCE plus fulvestrant. No dose reduction was allowed for fulvestrant in Study 2.

Permanent discontinuation associated with an adverse reaction occurred in 19 of 345 (6%) patients receiving IBRANCE plus fulvestrant, and in 6 of 172 (3%) patients receiving placebo plus fulvestrant. Adverse reactions leading to discontinuation for those patients receiving IBRANCE plus fulvestrant included fatigue (0.6%), infections (0.6%), and thrombocytopenia (0.6%).

The most common adverse reactions (≥10%) of any grade reported in patients in the IBRANCE plus fulvestrant arm by descending frequency were neutropenia, leukopenia, infections, fatigue, nausea, anemia, stomatitis, diarrhea, thrombocytopenia, vomiting, alopecia, rash, decreased appetite, and pyrexia.

The most frequently reported Grade ≥3 adverse reactions (≥5%) in patients receiving IBRANCE plus fulvestrant in descending frequency were neutropenia and leukopenia.

Adverse reactions (≥10%) reported in patients who received IBRANCE plus fulvestrant or placebo plus fulvestrant in Study 2 are listed in Table 6.

1. **Table 6. Adverse Reactions (≥10%) in Study 2**

| **Adverse Reaction** | **IBRANCE plus Fulvestrant (N=345)** | | | **Placebo plus Fulvestrant (N=172)** | | |
| --- | --- | --- | --- | --- | --- | --- |
|  | **All Grades** | **Grade 3** | **Grade 4** | **All Grades** | **Grade 3** | **Grade 4** |
|  | **%** | **%** | **%** | **%** | **%** | **%** |
| Infections and infestations | | | | | | |
| Infections | 47b | 3 | 1 | 31 | 3 | 0 |
| Blood and lymphatic system disorders | | | | | | |
| Neutropenia Leukopenia Anemia Thrombocytopenia | 83  53  30  23 | 55  30  4  2 | 11  1  0  1 | 4  5  13  0 | 1  1  2  0 | 0  1  0  0 |
| Metabolism and nutrition disorders | | | | | | |
| Decreased appetite | 16 | 1 | 0 | 8 | 1 | 0 |
| Gastrointestinal disorders | | | | | | |
| Nausea Stomatitis Diarrhea Vomiting | 34  28  24  19 | 0  1  0  1 | 0  0  0  0 | 28  13  19  15 | 1  0  1  1 | 0  0  0  0 |
| Skin and subcutaneous tissue disorders | | | | | | |
| Alopecia Rash | 18d17 | N/A 1 | N/A 0 | 6e  6 | N/A 0 | N/A 0 |
| General disorders and administration site conditions | | | | | | |
| Fatigue Pyrexia | 41  13 | 2  <1 | 0  0 | 29  5 | 1  0 | 0  0 |

Grading according to CTCAE 4.0.

CTCAE=Common Terminology Criteria for Adverse Events; N=number of patients; N/A=not applicable.

a. Infections includes all reported preferred terms (PTs) that are part of the System Organ Class Infections and infestations.

b. Most common infections (>1%) include: nasopharyngitis, upper respiratory tract infection, urinary tract

infection, bronchitis, rhinitis, influenza, conjunctivitis, sinusitis, pneumonia, cystitis, oral herpes, respiratory tract infection, gastroenteritis, tooth infection, pharyngitis, eye infection, herpes simplex, and paronychia.

c. Stomatitis includes: aphthous stomatitis, cheilitis, glossitis, glossodynia, mouth ulceration, mucosal inflammation, oral pain, oropharyngeal discomfort, oropharyngeal pain, stomatitis.

d. Grade 1 events – 17%; Grade 2 events – 1%.

e. Grade 1 events – 6%.

f. Rash includes: rash, rash maculo-papular, rash pruritic, rash erythematous, rash papular, dermatitis, dermatitis acneiform, toxic skin eruption.

Additional adverse reactions occurring at an overall incidence of <10.0% of patients receiving IBRANCE plus fulvestrant in Study 2 included asthenia (7.5%), aspartate aminotransferase increased (7.5%), dysgeusia (6.7%), epistaxis (6.7%), lacrimation increased (6.4%), dry skin (6.1%), alanine aminotransferase increased (5.8%), vision blurred (5.8%), dry eye (3.8%), and febrile

neutropenia (0.9%).

1. **Table 7. Laboratory Abnormalities in Study 2**

| **Laboratory Abnormality** | **IBRANCE plus Fulvestrant (N=345)** | | | **Placebo plus Fulvestrant (N=172)** | | |
| --- | --- | --- | --- | --- | --- | --- |
|  | **All Grades**  **%** | **Grade 3**  **%** | **Grade 4**  **%** | **All Grades**  **%** | **Grade 3**  **%** | **Grade 4**  **%** |
| WBC decreased | 99 | 45 | 1 | 26 | 0 | 1 |
| Neutrophils decreased | 96 | 56 | 11 | 14 | 0 | 1 |
| Anemia | 78 | 3 | 0 | 40 | 2 | 0 |
| Platelets decreased | 62 | 2 | 1 | 10 | 0 | 0 |
| Aspartate aminotransferase increased | 43 | 4 | 0 | 48 | 4 | 0 |
| Alanine aminotransferase increased | 36 | 2 | 0 | 34 | 0 | 0 |

N=number of patients; WBC=white blood cells.

1. **DRUG INTERACTIONS**

Palbociclib is primarily metabolized by CYP3A and sulfotransferase (SULT) enzyme SULT2A1. In vivo, palbociclib is a time-dependent inhibitor of CYP3A.

- 1. **Agents That May Increase Palbociclib Plasma Concentrations Effect of CYP3A Inhibitors**

Coadministration of a strong CYP3A inhibitor (itraconazole) increased the plasma exposure of palbociclib in healthy subjects by 87%. Avoid concomitant use of strong CYP3A inhibitors

(e.g., clarithromycin, indinavir, itraconazole, ketoconazole, lopinavir/ritonavir, nefazodone, nelfinavir, posaconazole, ritonavir, saquinavir, telaprevir, telithromycin, and voriconazole). Avoid grapefruit or grapefruit juice during IBRANCE treatment. If coadministration of IBRANCE with a strong CYP3A inhibitor cannot be avoided, reduce the dose of IBRANCE *[see Dosage and Administration (2.2) and Clinical Pharmacology (12.3)]*.

- 1. **Agents That May Decrease Palbociclib Plasma Concentrations Effect of CYP3A Inducers**

Coadministration of a strong CYP3A inducer (rifampin) decreased the plasma exposure of palbociclib in healthy subjects by 85%. Avoid concomitant use of strong CYP3A inducers (e.g., phenytoin, rifampin, carbamazepine, enzalutamide, and St John’s Wort) *[see Clinical Pharmacology (12.3)]*.

- 1. **Drugs That May Have Their Plasma Concentrations Altered by Palbociclib**

Coadministration of midazolam with multiple doses of IBRANCE increased the midazolam plasma exposure by 61%, in healthy subjects, compared to administration of midazolam alone. The dose of the sensitive CYP3A substrate with a narrow therapeutic index (e.g., alfentanil, cyclosporine, dihydroergotamine, ergotamine, everolimus, fentanyl, pimozide, quinidine, sirolimus, and tacrolimus) may need to be reduced, as IBRANCE may increase its exposure *[see Clinical Pharmacology (12.3)]*.

1. **USE IN SPECIFIC POPULATIONS**
   1. **Pregnancy Risk Summary**

Based on findings from animal studies and its mechanism of action, IBRANCE can cause fetal harm when administered to a pregnant woman *[see Clinical Pharmacology (12.1)]*. There are no available data in pregnant women to inform the drug-associated risk. In animal reproduction studies, administration of palbociclib to pregnant rats and rabbits during organogenesis resulted in embryofetal toxicity at maternal exposures that were ≥4 times the human clinical exposure based on AUC *[see Data]*. Advise pregnant women of the potential risk to a fetus.

The estimated background risk of major birth defects and miscarriage for the indicated population is unknown. In the U.S. general population, the estimated background risk of major birth defects and miscarriage in clinically recognized pregnancies is 2%-4% and 15%-20%, respectively.

1. **Data**

Animal Data

In a fertility and early embryonic development study in female rats, palbociclib was administered orally for 15 days before mating through to Day 7 of pregnancy, which did not cause embryo toxicity at doses up to 300 mg/kg/day with maternal systemic exposures approximately 4 times the human exposure (AUC) at the recommended dose.

In embryo-fetal development studies in rats and rabbits, pregnant animals received oral doses of palbociclib up to 300 mg/kg/day and 20 mg/kg/day, respectively, during the period of organogenesis. The maternally toxic dose of 300 mg/kg/day was fetotoxic in rats, resulting in reduced fetal body weights. At doses ≥100 mg/kg/day in rats, there was an increased incidence of a skeletal variation (increased incidence of a rib present at the seventh cervical vertebra). At the maternally toxic dose of 20 mg/kg/day in rabbits, there was an increased incidence of skeletal variations, including small phalanges in the forelimb. At 300 mg/kg/day in rats and 20 mg/kg/day in rabbits, the maternal systemic exposures were approximately 4 and 9 times the human exposure (AUC) at the recommended dose, respectively.

CDK4/6 double knockout mice have been reported to die in late stages of fetal development (gestation Day 14.5 until birth) due to severe anemia. However, knockout mouse data may not be predictive of effects in humans due to differences in degree of target inhibition.

- 1. **Lactation Risk Summary**

There is no information regarding the presence of palbociclib in human milk, its effects on milk production, or the breastfed infant. Because of the potential for serious adverse reactions in breastfed infants from IBRANCE, advise a lactating woman not to breastfeed during treatment with IBRANCE and for 3 weeks after the last dose.

- 1. **Females and Males of Reproductive Potential Pregnancy Testing**

Based on animal studies, IBRANCE can cause fetal harm when administered to a pregnant woman [*see Use in Specific Populations (8.1)].*Females of reproductive potential should have a pregnancy test prior to starting treatment with IBRANCE.

1. **Contraception**

Females

IBRANCE can cause fetal harm when administered to a pregnant woman *[see Use in Specific Populations (8.1)]*. Advise females of reproductive potential to use effective contraception during treatment with IBRANCE and for at least 3 weeks after the last dose.

Males

Because of the potential for genotoxicity, advise male patients with female partners of reproductive potential to use effective contraception during treatment with IBRANCE and for 3 months after the last dose *[see Nonclinical Toxicology (13.1)]*.

1. **Infertility**

Males

Based on animal studies, IBRANCE may impair fertility in males of reproductive potential *[see Nonclinical Toxicology (13.1)]*.

- 1. **Pediatric Use**

The safety and efficacy of IBRANCE in pediatric patients have not been studied.

Altered glucose metabolism (glycosuria, hyperglycemia, decreased insulin) associated with changes in the pancreas (islet cell vacuolation), eye (cataracts, lens degeneration), kidney (tubule vacuolation, chronic progressive nephropathy) and adipose tissue (atrophy) were identified in a 27 week repeat-dose toxicology study in rats that were immature at the beginning of the studies and were most prevalent in males at oral palbociclib doses ≥ 30 mg/kg/day (approximately 11 times the adult human exposure [AUC] at the recommended dose). Some of these findings (glycosuria/hyperglycemia, pancreatic islet cell vacuolation, and kidney tubule vacuolation) were present with lower incidence and severity in a 15 week repeat-dose toxicology study in immature rats. Altered glucose metabolism or associated changes in the pancreas, eye, kidney and adipose tissue were not identified in a 27-week repeat-dose toxicology study in rats that were mature at the beginning of the study and in dogs in repeat-dose toxicology studies up to 39 weeks duration.

Toxicities in teeth independent of altered glucose metabolism were observed in rats. Administration of 100 mg/kg palbociclib for 27 weeks (approximately 15 times the adult human exposure [AUC] at the recommended dose) resulted in abnormalities in growing incisor teeth (discolored, ameloblast degeneration/necrosis, mononuclear cell infiltrate). Other toxicities of potential concern to pediatric patients have not been evaluated in juvenile animals.

- 1. **Geriatric Use**

Of 444 patients who received IBRANCE in Study 1, 181 patients (41%) were ≥65 years of age and 48 patients (11%) were ≥75 years of age. Of 347 patients who received IBRANCE in Study 2,

86 patients (25%) were ≥65 years of age and 27 patients (8%) were ≥75 years of age. No overall differences in safety or effectiveness of IBRANCE were observed between these patients and younger patients.

- 1. **Hepatic Impairment**

Based on a population pharmacokinetic analysis that included 183 patients, where 40 patients had mild hepatic impairment (total bilirubin ≤ ULN and AST > ULN, or total bilirubin >1.0 to 1.5 × ULN and any AST), mild hepatic impairment had no effect on the exposure of palbociclib. The pharmacokinetics of palbociclib have not been studied in patients with moderate or severe hepatic impairment (total bilirubin >1.5 × ULN and any AST) *[see Clinical Pharmacology (12.3)]*.

Review the Full Prescribing Information for the aromatase inhibitor or fulvestrant for dose modifications related to hepatic impairment.

- 1. **Renal Impairment**

Based on a population pharmacokinetic analysis that included 183 patients, where 73 patients had mild renal impairment (60 mL/min ≤ CrCl <90 mL/min) and 29 patients had moderate renal impairment

(30 mL/min ≤ CrCl <60 mL/min), mild and moderate renal impairment had no effect on the exposure of palbociclib. The pharmacokinetics of palbociclib have not been studied in patients with severe renal impairment *[see Clinical Pharmacology (12.3)]*.

1. **OVERDOSAGE**

There is no known antidote for IBRANCE. The treatment of overdose of IBRANCE should consist of general supportive measures.

1. **DESCRIPTION**

IBRANCE capsules for oral administration contain 125 mg, 100 mg, or 75 mg of palbociclib, a kinase inhibitor. The molecular formula for palbociclib is C24H29N7O2. The molecular weight is

447.54 daltons. The chemical name is 6-acetyl-8-cyclopentyl-5-methyl-2-{[5-(piperazin-1-yl) pyridin-2­ yl] amino} pyrido [2, 3-*d*] pyrimidin-7(8*H*)-one, and its structural formula is:

Palbociclib is a yellow to orange powder with pKa of 7.4 (the secondary piperazine nitrogen) and

3.9 (the pyridine nitrogen). At or below pH 4, palbociclib behaves as a high-solubility compound. Above pH 4, the solubility of the drug substance reduces significantly.

*Inactive ingredients:*Microcrystalline cellulose, lactose monohydrate, sodium starch glycolate, colloidal silicon dioxide, magnesium stearate, and hard gelatin capsule shells. The light orange, light orange/caramel, and caramel opaque capsule shells contain gelatin, red iron oxide, yellow iron oxide, and titanium dioxide; the printing ink contains shellac, titanium dioxide, ammonium hydroxide, propylene glycol, and simethicone.

1. **CLINICAL PHARMACOLOGY**
   1. **Mechanism of Action**

Palbociclib is an inhibitor of cyclin-dependent kinases (CDK) 4 and 6. Cyclin D1 and CDK4/6 are downstream of signaling pathways which lead to cellular proliferation. In vitro, palbociclib reduced cellular proliferation of estrogen receptor (ER)-positive breast cancer cell lines by blocking progression of the cell from G1 into S phase of the cell cycle. Treatment of breast cancer cell lines with the combination of palbociclib and antiestrogens leads to decreased retinoblastoma (Rb) protein phosphorylation resulting in reduced E2F expression and signaling, and increased growth arrest compared to treatment with each drug alone. In vitro treatment of ER-positive breast cancer cell lines with the combination of palbociclib and antiestrogens led to increased cell senescence compared to each drug alone, which was sustained for up to 6 days following palbociclib removal and was greater if antiestrogen treatment was continued. In vivo studies using a patient-derived ER-positive breast cancer xenograft model demonstrated that the combination of palbociclib and letrozole increased the inhibition of Rb phosphorylation, downstream signaling, and tumor growth compared to each drug alone.

Human bone marrow mononuclear cells treated with palbociclib in the presence or absence of an anti- estrogen in vitro did not become senescent and resumed proliferation following palbociclib withdrawal.

- 1. **Pharmacodynamics Cardiac Electrophysiology**

The effect of palbociclib on the QT interval corrected for heart rate (QTc) was evaluated using time- matched electrocardiograms (ECGs) evaluating the change from baseline and corresponding pharmacokinetic data in 77 patients with breast cancer. Palbociclib had no large effect on QTc (i.e. > 20 ms) at 125 mg once daily (Schedule 3/1).

- 1. **Pharmacokinetics**

The pharmacokinetics (PK) of palbociclib were characterized in patients with solid tumors including advanced breast cancer and in healthy subjects.

1. **Absorption**

The mean maximum observed concentration (Cmax) of palbociclib is generally observed between 6 to 12 hours (time to reach maximum concentration, Tmax) following oral administration. The mean

absolute bioavailability of IBRANCE after an oral 125 mg dose is 46%. In the dosing range of 25 mg to 225 mg, the AUC and Cmax increased proportionally with dose in general. Steady state was achieved within 8 days following repeated once daily dosing. With repeated once daily administration, palbociclib accumulated with a median accumulation ratio of 2.4 (range 1.5 to 4.2).

Food effect: Palbociclib absorption and exposure were very low in approximately 13% of the population under the fasted condition. Food intake increased the palbociclib exposure in this small subset of the

population, but did not alter palbociclib exposure in the rest of the population to a clinically relevant extent. Therefore, food intake reduced the intersubject variability of palbociclib exposure, which supports administration of IBRANCE with food. Compared to IBRANCE given under overnight fasted conditions, the population average area under the concentration-time curve from zero to infinity (AUCINF) and Cmax of palbociclib increased by 21% and 38%, respectively, when given with high-fat, high-calorie food (approximately 800 to 1000 calories with 150, 250, and 500 to 600 calories from protein, carbohydrate, and fat, respectively), by 12% and 27%, respectively, when given with low-fat, low-calorie food (approximately 400 to 500 calories with 120, 250, and 28 to 35 calories from protein, carbohydrate, and fat, respectively), and by 13% and 24%, respectively, when moderate-fat, standard calorie food (approximately 500 to 700 calories with 75 to 105, 250 to 350 and 175 to 245 calories from protein, carbohydrate, and fat, respectively) was given 1 hour before and 2 hours after IBRANCE dosing.

1. **Distribution**

Binding of palbociclib to human plasma proteins in vitro was approximately 85%, with no concentration dependence over the concentration range of 500 ng/mL to 5000 ng/mL. The geometric mean apparent volume of distribution (Vz/F) was 2583 L with a coefficient of variation (CV) of 26%.

1. **Metabolism**

In vitro and in vivo studies indicated that palbociclib undergoes hepatic metabolism in humans. Following oral administration of a single 125 mg dose of [14C]palbociclib to humans, the primary metabolic pathways for palbociclib involved oxidation and sulfonation, with acylation and glucuronidation contributing as minor pathways. Palbociclib was the major circulating drug-derived entity in plasma (23%). The major circulating metabolite was a glucuronide conjugate of palbociclib, although it only represented 1.5% of the administered dose in the excreta. Palbociclib was extensively metabolized with unchanged drug accounting for 2.3% and 6.9% of radioactivity in feces and urine, respectively. In feces, the sulfamic acid conjugate of palbociclib was the major drug-related component, accounting for 26% of the administered dose. In vitro studies with human hepatocytes, liver cytosolic and S9 fractions, and recombinant SULT enzymes indicated that CYP3A and SULT2A1 are mainly involved in the metabolism of palbociclib.

1. **Elimination**

The geometric mean apparent oral clearance (CL/F) of palbociclib was 63.1 L/hr (29% CV), and the mean (± standard deviation) plasma elimination half-life was 29 (±5) hours in patients with advanced breast cancer. In 6 healthy male subjects given a single oral dose of [14C]palbociclib, a median of 91.6% of the total administered radioactive dose was recovered in 15 days; feces (74.1% of dose) was the major route of excretion, with 17.5% of the dose recovered in urine. The majority of the material was excreted as metabolites.

1. **Age, Gender, and Body Weight**

Based on a population pharmacokinetic analysis in 183 patients with cancer (50 male and 133 female patients, age range from 22 to 89 years, and body weight range from 37.9 to 123 kg), gender had no effect on the exposure of palbociclib, and age and body weight had no clinically important effect on the exposure of palbociclib.

1. **Pediatric Population**

Pharmacokinetics of IBRANCE have not been evaluated in patients <18 years of age.

1. **Drug Interactions**

In vitro data indicate that CYP3A and SULT enzyme SULT2A1 are mainly involved in the metabolism of palbociclib. Palbociclib is a weak time-dependent inhibitor of CYP3A following daily 125 mg dosing to steady state in humans. In vitro, palbociclib is not an inhibitor of CYP1A2, 2A6, 2B6, 2C8, 2C9, 2C19, and 2D6, and is not an inducer of CYP1A2, 2B6, 2C8, and 3A4 at clinically relevant concentrations.

*CYP3A Inhibitors:*Data from a drug interaction trial in healthy subjects (N=12) indicate that coadministration of multiple 200 mg daily doses of itraconazole with a single 125 mg IBRANCE dose increased palbociclib AUCINF and the Cmax by approximately 87% and 34%, respectively, relative to a single 125 mg IBRANCE dose given alone *[see Drug Interactions (7.1)]*.

*CYP3A Inducers:*Data from a drug interaction trial in healthy subjects (N=15) indicate that coadministration of multiple 600 mg daily doses of rifampin, a strong CYP3A inducer, with a single 125 mg IBRANCE dose decreased palbociclib AUCINF and Cmax by 85% and 70%, respectively, relative to a single 125 mg IBRANCE dose given alone. Data from a drug interaction trial in healthy subjects (N=14) indicate that coadministration of multiple 400 mg daily doses of modafinil, a moderate

CYP3A inducer, with a single 125 mg IBRANCE dose decreased palbociclib AUCINF and Cmax by 32% and 11%, respectively, relative to a single 125 mg IBRANCE dose given alone *[see Drug Interactions (7.2)]*.

*CYP3A Substrates:*Palbociclib is a weak time-dependent inhibitor of CYP3A following daily 125 mg dosing to steady state in humans. In a drug interaction trial in healthy subjects (N=26), coadministration of midazolam with multiple doses of IBRANCE increased the midazolam AUCINF and the Cmax values by 61% and 37%, respectively, as compared to administration of midazolam alone *[see Drug Interactions (7.3)]*.

*Gastric pH Elevating Medications:*In a drug interaction trial in healthy subjects, coadministration of a single 125 mg dose of IBRANCE with multiple doses of the proton pump inhibitor (PPI) rabeprazole under fed conditions decreased palbociclib Cmax by 41%, but had limited impact on AUCINF

(13% decrease), when compared to a single dose of IBRANCE administered alone. Given the reduced effect on gastric pH of H2-receptor antagonists and local antacids compared to PPIs, the effect of these classes of acid-reducing agents on palbociclib exposure under fed conditions is expected to be minimal. Under fed conditions there is no clinically relevant effect of PPIs, H2-receptor antagonists, or local antacids on palbociclib exposure. In another healthy subject study, coadministration of a single dose of IBRANCE with multiple doses of the PPI rabeprazole under fasted conditions decreased palbociclib AUCINF and Cmax by 62% and 80%, respectively, when compared to a single dose of IBRANCE administered alone.

*Letrozole:*Data from a clinical trial in patients with breast cancer showed that there was no drug interaction between palbociclib and letrozole when the 2 drugs were coadministered.

*Fulvestrant:*Data from a clinical trial in patients with breast cancer showed that there was no clinically relevant drug interaction between palbociclib and fulvestrant when the 2 drugs were coadministered.

*Goserelin:*Data from a clinical trial in patients with breast cancer showed that there was no clinically relevant drug interaction between palbociclib and goserelin when the 2 drugs were coadministered.

*Anastrozole or exemestane:*No clinical data are available to evaluate drug interactions between anastrozole or exemestane and palbociclib. A clinically significant drug interaction between anastrozole or exemestane and palbociclib is not expected based on analyses of the effects of anastrozole, exemestane and palbociclib on or by metabolic pathways or transporter systems.

*Effect of Palbociclib on Transporters:*In vitro evaluations indicated that palbociclib has a low potential to inhibit the activities of drug transporters P-glycoprotein (P-gp), breast cancer resistance protein (BCRP), organic anion transporter (OAT)1, OAT3, organic cation transporter (OCT)2, and organic anion transporting polypeptide (OATP)1B1, OATP1B3 at clinically relevant concentrations.

*Effect of Transporters on Palbociclib:*Based on in vitro data, P-gp and BCRP mediated transport are unlikely to affect the extent of oral absorption of palbociclib at therapeutic doses.

1. **NONCLINICAL TOXICOLOGY**
   1. **Carcinogenesis, Mutagenesis, Impairment of Fertility**

Carcinogenicity studies have not been conducted with palbociclib.

Palbociclib was aneugenic in Chinese Hamster Ovary cells in vitro and in the bone marrow of male rats at doses ≥100 mg/kg/day for 3 weeks. Palbociclib was not mutagenic in an in vitro bacterial reverse mutation (Ames) assay and was not clastogenic in the in vitro human lymphocyte chromosome aberration assay.

In a fertility study in female rats, palbociclib did not affect mating or fertility at any dose up to

300 mg/kg/day (approximately 4 times human clinical exposure based on AUC) and no adverse effects were observed in the female reproductive tissues in repeat-dose toxicity studies up to 300 mg/kg/day in the rat and 3 mg/kg/day in the dog (approximately 6 times and similar to human exposure [AUC], at the recommended dose, respectively).

The adverse effects of palbociclib on male reproductive function and fertility were observed in the repeat-dose toxicology studies in rats and dogs and a male fertility study in rats. In repeat-dose toxicology studies, palbociclib-related findings in the testis, epididymis, prostate, and seminal vesicle at

≥30 mg/kg/day in rats and ≥0.2 mg/kg/day in dogs included decreased organ weight, atrophy or degeneration, hypospermia, intratubular cellular debris, and decreased secretion. Partial reversibility of male reproductive organ effects was observed in the rat and dog following a 4- and 12-week non-dosing period, respectively. These doses in rats and dogs resulted in approximately ≥10 and 0.1 times, respectively, the exposure [AUC] in humans at the recommended dose. In the fertility and early embryonic development study in male rats, palbociclib caused no effects on mating but resulted in a slight decrease in fertility in association with lower sperm motility and density at 100 mg/kg/day with projected exposure levels [AUC] of 20 times the exposure in humans at the recommended dose.

1. **14 CLINICAL STUDIES Study 1: IBRANCE plus Letrozole**
2. **Patients with ER-positive, HER2-negative advanced or metastatic breast cancer for initial endocrine based therapy**

Study 1 (PALOMA-2) was an international, randomized, double-blind, parallel-group, multicenter study of IBRANCE plus letrozole versus placebo plus letrozole conducted in postmenopausal women with

ER-positive, HER2-negative advanced breast cancer who had not received previous systemic treatment for their advanced disease. A total of 666 patients were randomized 2:1 to IBRANCE plus letrozole or placebo plus letrozole. Randomization was stratified by disease site (visceral versus non-visceral), disease-free interval (de novo metastatic versus ≤12 months from the end of adjuvant treatment to disease recurrence versus >12 months from the end of adjuvant treatment to disease recurrence), and nature of prior (neo) adjuvant anticancer therapies (prior hormonal therapies versus no prior hormonal therapy). IBRANCE was given orally at a dose of 125 mg daily for 21 consecutive days followed by

7 days off treatment. Patients received study treatment until objective disease progression, symptomatic deterioration, unacceptable toxicity, death, or withdrawal of consent, whichever occurred first. The major efficacy outcome of the study was investigator-assessed progression-free survival (PFS) evaluated according to Response Evaluation Criteria in Solid Tumors Version 1.1 (RECIST).

Patients enrolled in this study had a median age of 62 years (range 28 to 89). The majority of patients were White (78%), and most patients had an Eastern Cooperative Oncology Group (ECOG) performance status (PS) of 0 or 1 (98%). Forty-eight percent of patients had received chemotherapy and 56% had received antihormonal therapy in the neoadjuvant or adjuvant setting prior to their diagnosis of advanced breast cancer. Thirty-seven percent of patients had no prior systemic therapy in the neoadjuvant or adjuvant setting. The majority of patients (97%) had metastatic disease. Twenty-three percent of patients had bone only disease, and 49% of patients had visceral disease.

Major efficacy results from Study 1 are summarized in Table 8 and Figure 1. Consistent results were observed across patient subgroups of disease-free interval (DFI), disease site, and prior therapy. The treatment effect of the combination on PFS was also supported by an independent review of radiographs. The overall survival (OS) data were not mature at the time of the final PFS analysis (20% of patients had died). Patients will continue to be followed for the final analysis.

1. **Table 8. Efficacy Results – Study 1 (Investigator Assessment, Intent-to-Treat Population)**

|  | **IBRANCE**  **plus Letrozole** | **Placebo plus Letrozole** |
| --- | --- | --- |
| **Progression-free survival for ITT** | **N=444** | **N=222** |
| Number of PFS events (%) | 194 (43.7) | 137 (61.7) |
| Median progression-free survival (months, 95% CI) | 24.8 (22.1, NE) | 14.5 (12.9, 17.1) |
| Hazard ratio (95% CI) and p-value | 0.576 (0.463, 0.718), p<0.0001 | |
| **Objective Response for patients with measurable disease** | **N=338** | **N=171** |
| Objective response rate* (%, 95% CI) | 55.3 (49.9, 60.7) | 44.4 (36.9, 52.2) |
|  |  | |

*Response based on confirmed responses

CI=confidence interval; ITT=Intent-to-Treat; N=number of patients; NE=not estimable.

1. **Figure 1. Kaplan-Meier Plot of Progression-Free Survival – Study 1 (Investigator Assessment, Intent-to-Treat Population)**

**Study 2: IBRANCE plus Fulvestrant**

1. **Patients with HR-positive, HER2-negative advanced or metastatic breast cancer who have had disease progression on or after prior adjuvant or metastatic endocrine therapy**

Study 2 (PALOMA-3) was an international, randomized, double-blind, parallel group, multicenter study of IBRANCE plus fulvestrant versus placebo plus fulvestrant conducted in women with HR-positive, HER2-negative advanced breast cancer, regardless of their menopausal status, whose disease progressed on or after prior endocrine therapy. A total of 521 pre/postmenopausal women were randomized 2:1 to IBRANCE plus fulvestrant or placebo plus fulvestrant and stratified by documented sensitivity to prior hormonal therapy, menopausal status at study entry (pre/peri versus postmenopausal), and presence of visceral metastases. IBRANCE was given orally at a dose of 125 mg daily for 21 consecutive days followed by 7 days off treatment. Pre/perimenopausal women were enrolled in the study and received the LHRH agonist goserelin for at least 4 weeks prior to and for the duration of Study 2. Patients continued to receive assigned treatment until objective disease progression, symptomatic deterioration, unacceptable toxicity, death, or withdrawal of consent, whichever occurred first. The major efficacy outcome of the study was investigator-assessed PFS evaluated according to RECIST 1.1.

Patients enrolled in this study had a median age of 57 years (range 29 to 88). The majority of patients on study were White (74%), all patients had an ECOG PS of 0 or 1, and 80% were postmenopausal. All patients had received prior systemic therapy, and 75% of patients had received a previous chemotherapy regimen. Twenty-five percent of patients had received no prior therapy in the metastatic disease setting, 60% had visceral metastases, and 23% had bone only disease.

The results from the investigator-assessed PFS from Study 2 are summarized in Table 9 and Figure 2. Consistent results were observed across patient subgroups of disease site, sensitivity to prior hormonal therapy and menopausal status. The overall survival (OS) data were not mature at the time of the final PFS analysis (11% of patients had died). Patients will continue to be followed for the final analysis.

**Table 9. Efficacy Results – Study 2 (Investigator Assessment, Intent-to-Treat Population)**

**Figure 2. Kaplan-Meier Plot of Progression-Free Survival (Investigator Assessment, Intent-to- Treat Population) – Study 2**

**16. HOW SUPPLIED/STORAGE AND HANDLING**

IBRANCE is supplied in the following strengths and package configurations:

| **IBRANCE Capsules** | | | |
| --- | --- | --- | --- |
| **Package Configuration** | **Capsule Strength (mg)** | **NDC** | **Capsule Description** |
| Bottles of 21 capsules | 125 | NDC 0069-0189-21 | opaque, hard gelatin capsules, size 0, with caramel cap and body, printed with white ink “Pfizer” on the cap, |

| **IBRANCE Capsules** | | | |
| --- | --- | --- | --- |
| **Package Configuration** | **Capsule Strength (mg)** | **NDC** | **Capsule Description** |
|  |  |  | “PBC 125” on the body |
| Bottles of 21 capsules | 100 | NDC 0069-0188-21 | opaque, hard gelatin capsules, size 1, with caramel cap and light orange body, printed with white ink “Pfizer” on the cap, “PBC 100” on the body |
| Bottles of 21 capsules | 75 | NDC 0069-0187-21 | opaque, hard gelatin capsules, size 2, with light orange cap and body, printed with white ink “Pfizer” on the cap, “PBC 75” on the body |

Store at 20 oC to 25 oC (68 oF to 77 oF); excursions permitted between 15 oC to 30 oC (59 oF to 86 oF).

*[see USP Controlled Room Temperature]*.

**17. PATIENT COUNSELING INFORMATION**

Advise the patient to read the FDA-approved patient labeling (Patient Information). Myelosuppression/Infection

- Advise patients to immediately report any signs or symptoms of myelosuppression or infection,

such as fever, chills, dizziness, shortness of breath, weakness, or any increased tendency to bleed and/or to bruise *[see Warnings and Precautions (5.1)]*.

Drug Interactions

- Grapefruit may interact with IBRANCE. Patients should not consume grapefruit products while on treatment with IBRANCE.
- Inform patients to avoid strong CYP3A inhibitors and strong CYP3A inducers.
- Advise patients to inform their health care providers of all concomitant medications, including prescription medicines, over-the-counter drugs, vitamins, and herbal products *[see Drug Interactions (7)]*.

Dosing and Administration

- Advise patients to take IBRANCE with food.
- If the patient vomits or misses a dose, an additional dose should not be taken. The next prescribed dose should be taken at the usual time. IBRANCE capsules should be swallowed whole (do not chew, crush, or open them prior to swallowing). No capsule should be ingested if it is broken, cracked, or otherwise not intact.

Pregnancy, Lactation, and Fertility

- Embryo-Fetal Toxicity
  - Advise females of reproductive potential of the potential risk to a fetus and to use effective contraception during treatment with IBRANCE therapy and for at least 3 weeks after the last dose. Advise females to inform their healthcare provider of a known or suspected pregnancy *[see Warnings and Precautions (5.2) and Use in Specific Populations (8.1 and 8.3)]*.
  - Advise male patients with female partners of reproductive potential to use effective contraception during treatment with IBRANCE and for at least 3 months after the last dose *[see Use in Specific Populations (8.3)].*
- Lactation: Advise women not to breastfeed during treatment with IBRANCE and for 3 weeks after the last dose *[see Use in Specific Populations (8.2)].*

35T[This product’s label may have been updated. For full prescribing information, please visit www.IBRANCE.com.](http://www.ibrance.com/)


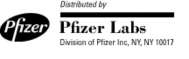


LAB-0723-2.1

| **PATIENT INFORMATION**  **IBRANCE® (EYE-brans) (palbociclib)**  Capsules |
| --- |
| **What is the most important information I should know about IBRANCE? IBRANCE may cause serious side effects, including:**  **Low white blood cell counts (neutropenia).**Low white blood cell counts are very common when taking  IBRANCE and may cause serious infections that can lead to death. Your healthcare provider should check your white blood cell counts before and during treatment.  If you develop low white blood cell counts during treatment with IBRANCE, your healthcare provider may stop your treatment, decrease your dose, or may tell you to wait to begin your treatment cycle. Tell your healthcare provider right away if you have signs and symptoms of low white blood cell counts or infections such as fever and chills.  **See “What are the possible side effects of IBRANCE?” for more information about side effects.** |
| **What is IBRANCE?**  IBRANCE is a prescription medicine used to treat hormone receptor (HR)-positive, human epidermal growth factor receptor 2 (HER2)-negative breast cancer that has spread to other parts of the body (metastatic) in combination with:   - an aromatase inhibitor as the first hormonal based therapy in women who have gone through menopause, or - fulvestrant in women with disease progression following hormonal therapy. It is not known if IBRANCE is safe and effective in children. |
| **What should I tell my healthcare provider before taking IBRANCE?**  Before you take IBRANCE, tell your healthcare provider if you:   - have fever, chills, or any other signs or symptoms of infection. - have liver or kidney problems. - have any other medical conditions. - are pregnant, or plan to become pregnant. IBRANCE can harm your unborn baby.   - Females who are able to become pregnant and who take IBRANCE should use effective birth control during treatment and for at least 3 weeks after stopping IBRANCE.   - Males who are taking IBRANCE, with female partners who can become pregnant should use effective birth control during treatment with IBRANCE for 3 months after the final dose of IBRANCE.   - Talk to your healthcare provider about birth control methods that may be right for you during this time.   - If you become pregnant or think you are pregnant, tell your healthcare provider right away. - are breastfeeding or plan to breastfeed. It is not known if IBRANCE passes into your breast milk. You and your healthcare provider should decide if you will take IBRANCE or breastfeed. You should not do both.   **Tell your healthcare provider about all of the medicines you take, including**prescription and over-the-counter medicines, vitamins, and herbal supplements. IBRANCE and other medicines may affect each other causing side effects.  Know the medicines you take. Keep a list of them to show your healthcare provider or pharmacist when you get a new medicine. |
| **How should I take IBRANCE?**   - Take IBRANCE exactly as your healthcare provider tells you. - Take IBRANCE with food. - Swallow IBRANCE capsules whole. Do not chew, crush or open IBRANCE capsules before swallowing them. - Do not take any IBRANCE capsules that are broken, cracked, or that look damaged. - Avoid grapefruit and grapefruit products during treatment with IBRANCE. Grapefruit may increase the amount of IBRANCE in your blood. - Do not change your dose or stop taking IBRANCE unless your healthcare provider tells you. - If you miss a dose of IBRANCE or vomit after taking a dose of IBRANCE, do not take another dose on that day. Take your next dose at your regular time. - If you take too much IBRANCE, call your healthcare provider right away or go to the nearest hospital emergency room. |

| **What are the possible side effects of IBRANCE?**  **IBRANCE may cause serious side effects. See “What is the most important information I should know about IBRANCE?”**  Common side effects of IBRANCE when used with either letrozole or fulvestrant include:   - Low red blood cell counts and low platelet counts are common with IBRANCE. Call your healthcare provider right away if you develop any of these symptoms during treatment:   - dizziness o bleeding or bruising more easily   - shortness of breath   - nosebleeds   - weakness - infections (see “What is the most important information I should know about IBRANCE?”) - diarrhea - hair thinning or hair loss - tiredness - vomiting - nausea - rash - sore mouth - loss of appetite - abnormalities in liver blood tests    BRANCE may cause fertility problems in males. This may affect your ability to father a child. Talk to your healthcare provider if this is a concern for you.  Tell your healthcare provider if you have any side effect that bothers you or that does not go away.  These are not all of the possible side effects of IBRANCE. For more information, ask your healthcare provider or pharmacist.  Call your doctor for medical advice about side effects. You may report side effects to FDA at 1-800-FDA-1088. |
| --- |
| **How should I store IBRANCE?**   - Store IBRANCE at 68 °F to 77 °F (20 °C to 25 °C).   **Keep IBRANCE and all medicines out of the reach of children.** |
| **General information about the safe and effective use of IBRANCE**  Medicines are sometimes prescribed for purposes other than those listed in a Patient Information leaflet. Do not use IBRANCE for a condition for which it was not prescribed. Do not give IBRANCE to other people, even if they have the same symptoms you have. It may harm them.  If you would like more information, talk with your healthcare provider. You can ask your pharmacist or healthcare provider for more information about IBRANCE that is written for health professionals.  [35TFor more information, go to](http://www.ibrance.com/)35Twww.IBRANCE.com or call 1-800-438-1985. |
| **What are the ingredients in IBRANCE?**  Active ingredient: palbociclib  Inactive ingredients: Microcrystalline cellulose, lactose monohydrate, sodium starch glycolate, colloidal silicon dioxide, magnesium stearate, and hard gelatin capsule shells.  Light orange, light orange/caramel and caramel opaque capsule shells contain: gelatin, red iron oxide, yellow iron oxide, and titanium dioxide.  Printing ink contains: shellac, titanium dioxide, ammonium hydroxide, propylene glycol, and simethicone.  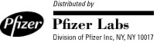  LAB-0724-2.1 |

This Patient Information has been approved by the U.S. Food and Drug Administration.

**APPENDIX B - Hydroxychloroquine (Plaquenil) Package Insert**

**PLAQUENIL®**

**HYDROXYCHLOROQUINE SULFATE, USP**

**WARNING**

PHYSICIANS SHOULD COMPLETELY FAMILIARIZE THEMSELVES WITH THE COMPLETE CONTENTS OF THIS LEAFLET BEFORE PRESCRIBING HYDROXYCHLOROQUINE.

1. **DESCRIPTION**

Hydroxychloroquine sulfate is a colorless crystalline solid, soluble in water to at least 20 percent; chemically the drug is 2-[[4-[(7-Chloro-4-quinolyl) amino]pentyl] ethylamino] ethanol sulfate (1:1).

PLAQUENIL (hydroxychloroquine sulfate) tablets contain 200 mg hydroxychloroquine sulfate, equivalent to 155 mg base, and are for oral administration.

*Inactive Ingredients:* Dibasic Calcium Phosphate, Hydroxypropyl Methylcellulose, Magnesium Stearate, Polyethylene glycol 400, Polysorbate 80, Starch, Titanium Dioxide.

1. **ACTIONS**

The drug possesses antimalarial actions and also exerts a beneficial effect in lupus erythematosus (chronic discoid or systemic) and acute or chronic rheumatoid arthritis. The precise mechanism of action is not known.

1. **INDICATIONS**

PLAQUENIL is indicated for the suppressive treatment and treatment of acute attacks of malaria due to *Plasmodium vivax*, *P. malariae*, *P. ovale*, and susceptible strains of *P. falciparum*. It is also indicated for the treatment of discoid and systemic lupus erythematosus, and rheumatoid arthritis.

1. **CONTRAlNDICATIONS**

Use of this drug is contraindicated (1) in the presence of retinal or visual field changes attributable to any 4-aminoquinoline compound, (2) in patients with known hypersensitivity to 4- aminoquinoline compounds, and (3) for long-term therapy in children.

1. **WARNINGS, General**

PLAQUENIL is not effective against chloroquine-resistant strains of *P. falciparum*.

Children are especially sensitive to the 4-aminoquinoline compounds. A number of fatalities have been reported following the accidental ingestion of chloroquine, sometimes in relatively small doses (0.75 g or 1 g in one 3-year-old child). Patients should be strongly warned to keep these drugs out of the reach of children.

Use of PLAQUENIL in patients with psoriasis may precipitate a severe attack of psoriasis. When used in patients with porphyria the condition may be exacerbated. The preparation should not be used in these conditions unless in the judgment of the physician the benefit to the patient outweighs the possible hazard.

***Usage in Pregnancy***—Usage of this drug during pregnancy should be avoided except in the suppression or treatment of malaria when in the judgment of the physician the benefit outweighs the possible hazard. It should be noted that radioactively-tagged chloroquine administered intravenously to pregnant, pigmented CBA mice passed rapidly across the placenta. It accumulated selectively in the melanin structures of the fetal eyes and was retained in the ocular tissues for five months after the drug had been eliminated from the rest of the body.

1. **PRECAUTIONS, General**

Antimalarial compounds should be used with caution in patients with hepatic disease or alcoholism or in conjunction with known hepatotoxic drugs.

Periodic blood cell counts should be made if patients are given prolonged therapy. If any severe blood disorder appears which is not attributable to the disease under treatment, discontinuation of the drug should be considered. The drug should be administered with caution in patients having G-6-PD (glucose-6-phosphate dehydrogenase) deficiency.

1. **OVERDOSAGE**

The 4-aminoquinoline compounds are very rapidly and completely absorbed after ingestion, and in accidental overdosage, or rarely with lower doses in hypersensitive patients, toxic symptoms may occur within 30 minutes. These consist of headache, drowsiness, visual disturbances, cardiovascular collapse, and convulsions, followed by sudden and early respiratory and cardiac arrest. The electrocardiogram may reveal atrial standstill, nodal rhythm, prolonged intraventricular conduction time, and progressive bradycardia leading to ventricular fibrillation and/or arrest. Treatment is symptomatic and must be prompt with immediate evacuation of the stomach by emesis (at home, before transportation to the hospital) or gastric lavage until the stomach is completely emptied. If finely powdered, activated charcoal is introduced by the stomach tube, after lavage, and within 30 minutes after ingestion of the tablets, it may inhibit further intestinal absorption of the drug. To be effective, the dose of activated charcoal should be at least five times the estimated dose of hydroxychloroquine ingested. Convulsions, if present, should be controlled before attempting gastric lavage. If due to cerebral stimulation, cautious administration of an ultrashort-acting barbiturate may be tried but, if due to anoxia, it should be corrected by oxygen administration, artificial respiration or, in shock with hypotension, by vasopressor therapy. Because of the importance of supporting respiration,

tracheal intubation or tracheostomy, followed by gastric lavage, may also be necessary. Exchange transfusions have been used to reduce the level of 4-aminoquinoline drug in the blood.

A patient who survives the acute phase and is asymptomatic should be closely observed for at least six hours. Fluids may be forced, and sufficient ammonium chloride (8 g daily in divided doses for adults) may be administered for a few days to acidify the urine to help promote urinary excretion in cases of both overdosage and sensitivity.


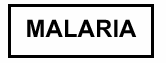


1. **Actions**

Like chloroquine phosphate, USP, PLAQUENIL is highly active against the erythrocytic forms of *P. vivax* and *malariae* and most strains of *P. falciparum* (but not the gametocytes of *P. falciparum*).

PLAQUENIL does not prevent relapses in patients with *vivax* or *malariae* malaria because it is not effective against exo-erythrocytic forms of the parasite, nor will it prevent *vivax* or *malariae* infection when administered as a prophylactic. It is highly effective as a suppressive agent in patients with *vivax* or *malariae* malaria, in terminating acute attacks, and significantly lengthening the interval between treatment and relapse. In patients with *falciparum* malaria, it abolishes the acute attack and effects complete cure of the infection, unless due to a resistant strain of *P. falciparum*.

1. **Indications**

PLAQUENIL is indicated for the treatment of acute attacks and suppression of malaria.

1. **Warning**

In recent years, it has been found that certain strains of *P. falciparum* have become resistant to 4-aminoquinoline compounds (including hydroxychloroquine) as shown by the fact that normally adequate doses have failed to prevent or cure clinical malaria or parasitemia.

Treatment with quinine or other specific forms of therapy is therefore advised for patients infected with a resistant strain of parasites.

1. **Adverse Reactions**

Following the administration in doses adequate for the treatment of an acute malarial attack, mild and transient headache, dizziness, and gastrointestinal complaints (diarrhea, anorexia, nausea, abdominal cramps and, on rare occasions, vomiting) may occur.

Cardiomyopathy has been rarely reported with high daily dosages of hydroxychloroquine.

1. **Dosage and Administration**

One tablet of 200 mg of hydroxychloroquine sulfate is equivalent to 155 mg base.

***Malaria:*** Suppression— *In adults*, 400 mg (=310 mg base) on exactly the same day of each week. *In infants and children*, the weekly suppressive dosage is 5 mg, calculated as base, per kg of body weight, but should not exceed the adult dose regardless of weight.

If circumstances permit, suppressive therapy should begin two weeks prior to exposure. However, failing this, in adults an initial double (loading) dose of 800 mg (=620 mg base), or in children 10 mg base/kg may be taken in two divided doses, six hours apart. The suppressive therapy should be continued for eight weeks after leaving the endemic area.

Treatment of the acute attack— *In adults*, an initial dose of 800 mg (= 620 mg base) followed by 400 mg (=310 mg base) in six to eight hours and 400 mg (=310 mg base) on each of two consecutive days (total 2 g hydroxychloroquine sulfate or 1.55 g base). An alternative method, employing a single dose of 800 mg (=620 mg base), has also proved effective.

The dosage for adults may also be calculated on the basis of body weight; this method is preferred for infants and children. A total dose representing 25 mg of base per kg of body weight is administered in three days, as follows:

First dose: 10 mg base per kg (but not exceeding a single dose of 620 mg base).

Second dose: 5 mg base per kg (but not exceeding a single dose of 310 mg base) 6 hours after first dose.

Third dose: 5 mg base per kg 18 hours after second dose. Fourth dose: 5 mg base per kg 24 hours after third dose.

For radical cure of *vivax* and *malariae* malaria concomitant therapy with an 8- aminoquinoline compound is necessary.

**LUPUS ERYTHEMATOSUS AND**

**RHEUMATOID ARTHRITIS**

1. **Indications**

PLAQUENIL is useful in patients with the following disorders who have not responded satisfactorily to drugs with less potential for serious side effects: lupus erythematosus (chronic discoid and systemic) and acute or chronic rheumatoid arthritis.

1. **Warnings**

PHYSICIANS SHOULD COMPLETELY FAMILIARIZE THEMSELVES WITH THE COMPLETE CONTENTS OF THIS LEAFLET BEFORE PRESCRlBlNG PLAQUENIL.

Irreversible retinal damage has been observed in some patients who had received long- term or high-dosage 4-aminoquinoline therapy for discoid and systemic lupus erythematosus, or rheumatoid arthritis. Retinopathy has been reported to be dose-related.

When prolonged therapy with any Antimalarial compound is contemplated, initial (base line) and periodic (every three months) ophthalmologic examinations (including visual acuity, expert slit-lamp, funduscopic, and visual field tests) should be performed.

If there is any indication of abnormality in the visual acuity, visual field, or retinal macular areas (such as pigmentary changes, loss of foveal reflex), or any visual symptoms (such as light flashes and streaks) which are not fully explainable by difficulties of accommodation or corneal opacities, the drug should be discontinued immediately and the patient closely observed

for possible progression. Retinal changes (and visual disturbances) may progress even after cessation of therapy.

All patients on long-term therapy with this preparation should be questioned and examined periodically, including the testing of knee and ankle reflexes, to detect any evidence of muscular weakness. If weakness occurs, discontinue the drug.

In the treatment of rheumatoid arthritis, if objective improvement (such as reduced joint swelling, increased mobility) does not occur within six months, the drug should be discontinued. Safe use of the drug in the treatment of juvenile arthritis has not been established.

1. **Precautions**

Dermatologic reactions to PLAQUENIL may occur and, therefore, proper care should be exercised when it is administered to any patient receiving a drug with a significant tendency to produce dermatitis.

The methods recommended for early diagnosis of “chloroquine retinopathy” consist of

1. funduscopic examination of the macula for fine pigmentary disturbances or loss of the foveal reflex and (2) examination of the central visual field with a small red test object for pericentral or paracentral scotoma or determination of retinal thresholds to red. Any unexplained visual symptoms, such as light flashes or streaks should also be regarded with suspicion as possible manifestations of retinopathy.

If serious toxic symptoms occur from overdosage or sensitivity, it has been suggested that ammonium chloride (8 g daily in divided doses for adults) be administered orally three or four days a week for several months after therapy has been stopped, as acidification of the urine increases renal excretion of the 4-aminoquinoline compounds by 20 to 90 percent. However, caution must be exercised in patients with impaired renal function and/or metabolic acidosis.

1. **Adverse Reactions**

Not all of the following reactions have been observed with every 4-aminoquinoline compound during long-term therapy, but they have been reported with one or more and should be borne in mind when drugs of this class are administered. Adverse effects with different compounds vary in type and frequency.

***CNS Reactions:*** Irritability, nervousness, emotional changes, nightmares, psychosis, headache, dizziness, vertigo, tinnitus, nystagmus, nerve deafness, convulsions, ataxia.

***Neuromuscular Reactions:*** Skeletal muscle palsies or skeletal muscle myopathy or neuromyopathy leading to progressive weakness and atrophy of proximal muscle groups which may be associated with mild sensory changes, depression of tendon reflexes and abnormal nerve conduction.

***Ocular Reactions:***

- 1. *Ciliary body*: Disturbance of accommodation with symptoms of blurred vision. This reaction is dose-related and reversible with cessation of therapy.
  2. *Cornea*: Transient edema, punctate to lineal opacities, decreased corneal sensitivity. The corneal changes, with or without accompanying symptoms (blurred vision, halos around lights, photophobia), are fairly common, but reversible. Corneal deposits may appear as early as three weeks following initiation of therapy.

The incidence of corneal changes and visual side effects appears to be considerably lower with hydroxychloroquine than with chloroquine.

- 1. *Retina: Macula*: Edema, atrophy, abnormal pigmentation (mild pigment stippling to a “bull’s-eye” appearance), loss of foveal reflex, increased macular recovery time following exposure to a bright light (photo-stress test), elevated retinal threshold to red light in macular, paramacular, and peripheral retinal areas.

Other fundus changes include optic disc pallor and atrophy, attenuation of retinal arterioles, fine granular pigmentary disturbances in the peripheral retina and prominent choroidal patterns in advanced stage.

- 1. *Visual field defects*: Pericentral or paracentral scotoma, central scotoma with decreased visual acuity, rarely field constriction, abnormal color vision.

The most common visual symptoms attributed to the retinopathy are: reading and seeing difficulties (words, letters, or parts of objects missing), photophobia, blurred distance vision, missing or blacked out areas in the central or peripheral visual field, light flashes and streaks.

Retinopathy appears to be dose related and has occurred within several months (rarely) to several years of daily therapy; a small number of cases have been reported several years after antimalarial drug therapy was discontinued. It has not been noted during prolonged use of weekly doses of the 4-aminoquinoline compounds for suppression of malaria.

Patients with retinal changes may have visual symptoms or may be asymptomatic (with or without visual field changes). Rarely scotomatous vision or field defects may occur without obvious retinal change.

Retinopathy may progress even after the drug is discontinued. In a number of patients, early retinopathy (macular pigmentation sometimes with central field defects) diminished or regressed completely after therapy was discontinued. Paracentral scotoma to red targets (sometimes called “premaculopathy”) is indicative of early retinal dysfunction which is usually reversible with cessation of therapy.

A small number of cases of retinal changes have been reported as occurring in patients who received only hydroxychloroquine. These usually consisted of alteration in retinal pigmentation which was detected on periodic ophthalmologic examination; visual field defects were also present in some instances. A case of delayed retinopathy has been reported with loss of vision starting one year after administration of hydroxychloroquine had been discontinued.

***Dermatologic Reactions:*** Bleaching of hair, alopecia, pruritus, skin and mucosal pigmentation, photosentivity, and skin eruptions (urticarial, morbilliform, lichenoid, maculopapular, purpuric, erythema annulare centrifugum, Stevens-Johnson syndrome, acute generalized exanthematous pustulosis, and exfoliative dermatitis).

***Hematologic Reactions:*** Various blood dyscrasias such as aplastic anemia, agranulocytosis, leukopenia, anemia, thrombocytopenia (hemolysis in individuals with glucose- 6-phosphate dehydrogenase (G-6-PD) deficiency).

***Gastrointestinal Reactions:*** Anorexia, nausea, vomiting, diarrhea, and abdominal cramps. Isolated cases of abnormal liver function and fulminant hepatic failure.

***Allergic reactions:*** Urticaria, angioedema and bronchospasm have been reported.

***Miscellaneous Reactions:*** Weight loss, lassitude, exacerbation or precipitation of porphyria and nonlight-sensitive psoriasis.

Cardiomyopathy has been rarely reported with high daily dosages of hydroxychloroquine.

1. **Dosage and Administration**

One tablet of hydroxychloroquine sulfate, 200 mg, is equivalent to 155 mg base.

***Lupus erythematosus***—Initially, the average *adult* dose is 400 mg (=310 mg base) once or twice daily. This may be continued for several weeks or months, depending on the response of the patient. For prolonged maintenance therapy, a smaller dose, from 200 mg to 400 mg (=155 mg to 310 mg base) daily will frequently suffice.

The incidence of retinopathy has been reported to be higher when this maintenance dose is exceeded.

***Rheumatoid arthritis***—The compound is cumulative in action and will require several weeks to exert its beneficial therapeutic effects, whereas minor side effects may occur relatively early. Several months of therapy may be required before maximum effects can be obtained. If objective improvement (such as reduced joint swelling, increased mobility) does not occur within six months, the drug should be discontinued. Safe use of the drug in the treatment of juvenile rheumatoid arthritis has not been established.

*Initial dosage*—In **adults**, from 400 mg to 600 mg (=310 mg to 465 mg base) daily, each dose to be taken with a meal or a glass of milk. In a small percentage of patients, troublesome side effects may require temporary reduction of the initial dosage. Later (usually from five to ten days), the dose may gradually be increased to the optimum response level, often without return of side effects.

*Maintenance dosage*—When a good response is obtained (usually in four to twelve weeks), the dosage is reduced by 50 percent and continued at a usual maintenance level of 200 mg to 400 mg (=155 mg to 310 mg base) daily, each dose to be taken with a meal or a glass of milk. The incidence of retinopathy has been reported to be higher when this maintenance dose is exceeded.

Should a relapse occur after medication is withdrawn, therapy may be resumed or continued on an intermittent schedule if there are no ocular contraindications.

***Corticosteroids and salicylates*** may be used in conjunction with this compound, and they can generally be decreased gradually in dosage or eliminated after the drug has been used for several weeks. When gradual reduction of steroid dosage is indicated, it may be done by reducing every four to five days the dose of cortisone by no more than from 5 mg to 15 mg; of hydrocortisone from 5 mg to 10 mg; of prednisolone and prednisone from 1 mg to 2.5 mg; of methylprednisolone and triamcinolone from 1 mg to 2 mg; and of dexamethasone from 0.25 mg to 0.5 mg.

1. **HOW SUPPLIED**

PLAQUENIL tablets are white, to off-white, film coated tablets imprinted “PLAQUENIL” on one face in black ink. Each tablet contains 200 mg hydroxychloroquine sulfate (equivalent to 155 mg base). Bottles of 100 tablets (NDC 0024-1562-10).

Dispense in a tight, light-resistant container as defined in the USP/NF. Store at room temperature up to 30° C (86° F).

sanofi-aventis U.S. LLC, Bridgewater, NJ 08807

Revised October 2006

©2006 sanofi-aventis U.S. LLC

**APPENDIX C – Letrozole Package Insert**

**HIGHLIGHTS OF PRESCRIBING INFORMATION**

**These highlights do not include all the information needed to use FEMARA safely and effectively. See full prescribing information for FEMARA.**

**Femara (letrozole) tablets, for oral use Initial U.S. Approval: 1997**

**----------------------------RECENT MAJOR CHANGES--------------------------**

Contraindications [(4)](#_bookmark12) 7/2017

Warnings and Precautions, Embryo-Fetal Toxicity [(5.6)](#_bookmark19) 7/2017

**----------------------------INDICATIONS AND USAGE---------------------------**

Femara is an aromatase inhibitor indicated for:

- Adjuvant treatment of postmenopausal women with hormone receptor positive early breast cancer [(1.1)](#_bookmark1)
- Extended adjuvant treatment of postmenopausal women with early breast cancer who have received prior standard adjuvant tamoxifen therapy [(1.2)](#_bookmark2)
- First and second-line treatment of postmenopausal women with hormone receptor positive or unknown advanced breast cancer [(1.3)](#_bookmark3)

**-------------------------DOSAGE AND ADMINISTRATION---------------------**

Femara tablets are taken orally without regard to meals [(2)](#_bookmark4):

- Recommended dose: 2.5.mg once daily [(2.1)](#_bookmark5)
- Patients with cirrhosis or severe hepatic impairment: 2.5 mg every other day [(2.5,](#_bookmark9) [5.3)](#_bookmark16)

**-----------------------DOSAGE FORMS AND STRENGTHS--------------------**

2.5 mg tablets [(3)](#_bookmark11)

**--------------------------------CONTRAINDICATIONS-----------------------------**

- Pregnancy [(4)](#_bookmark12)
- Known hypersensitivity to the active substance, or to any of the excipients [(4)](#_bookmark12)

**-----------------------WARNINGS AND PRECAUTIONS-----------------------**

- Decreases in bone mineral density may occur. Consider bone mineral density monitoring [(5.1)](#_bookmark14)
- Increases in total cholesterol may occur. Consider cholesterol monitoring. [(5.2](#_bookmark15))
- Fatigue, dizziness and somnolence may occur. Exercise caution when operating machinery [(5.4)](#_bookmark17)
- Embryo-Fetal toxicity: Can cause fetal harm when administered to pregnant women. Obtain a pregnancy test in females of reproductive potential. Advise females of reproductive potential to use effective contraception [(5.6,](#_bookmark19) [8.1,](#_bookmark25) [8.3)](#_bookmark27)

**------------------------------ADVERSE REACTIONS-------------------------------**

The most common adverse reactions (greater than 20%) were hot flashes, arthralgia; flushing, asthenia, edema, arthralgia, headache, dizziness, hypercholesterolemia, sweating increased, bone pain; and musculoskeletal [(6)](#_bookmark20).

**To report SUSPECTED ADVERSE REACTIONS, contact Novartis Pharmaceuticals Corporation at 1-888-669-6682 or FDA at 1-800-FDA- 1088 or** [**www.fda.gov/medwatch.**](http://www.fda.gov/medwatch)

**------------------------------USE IN SPECIFIC POPULATIONS-----------------**

- Lactation: Advise not to breastfeed. [(8.2)](#_bookmark26)

**See 17 for PATIENT COUNSELING INFORMATION.**

**Revised: 11/2017**

**FULL PRESCRIBING INFORMATION: CONTENTS***

1. [**INDICATIONS AND USAGE**](#_bookmark0)
   1. [Adjuvant Treatment of Early Breast Cancer](#_bookmark1)
   2. [Extended Adjuvant Treatment of Early Breast Cancer](#_bookmark2)
   3. [First and Second-Line Treatment of Advanced Breast Cancer](#_bookmark3)
2. [**DOSAGE AND ADMINISTRATION**](#_bookmark4)
   1. [Recommended Dose](#_bookmark5)
   2. [Use in Adjuvant Treatment of Early Breast Cancer](#_bookmark6)
   3. [Use in Extended Adjuvant Treatment of Early Breast Cancer](#_bookmark7)
   4. [Use in First and Second-Line Treatment of Advanced Breast](#_bookmark8) [Cancer](#_bookmark8)
   5. [Use in Hepatic Impairment](#_bookmark9)
   6. [Use in Renal Impairment](#_bookmark10)
3. [**DOSAGE FORMS AND STRENGTHS**](#_bookmark11)
4. [**CONTRAINDICATIONS**](#_bookmark12)
5. [**WARNINGS AND PRECAUTIONS**](#_bookmark13)
   1. [Bone Effects](#_bookmark14)
   2. [Cholesterol](#_bookmark15)
   3. [Hepatic Impairment](#_bookmark16)
   4. [Fatigue and Dizziness](#_bookmark17)
   5. [Laboratory Test Abnormalities](#_bookmark18)
   6. [Embryo-Fetal Toxicity](#_bookmark19)
6. [**ADVERSE REACTIONS**](#_bookmark20)
   1. [Clinical Trials Experience](#_bookmark21)
   2. [Postmarketing Experience](#_bookmark22)
7. [**DRUG INTERACTIONS**](#_bookmark23)
8. [**USE IN SPECIFIC POPULATIONS**](#_bookmark24)
   1. [Pregnancy](#_bookmark25)
   2. [Lactation](#_bookmark26)
   3. [Females and Males of Reproductive Potential](#_bookmark27)
   4. [Pediatric Use](#_bookmark28)
   5. [Geriatric Use](#_bookmark29)
9. [**OVERDOSAGE**](#_bookmark30)
10. [**DESCRIPTION**](#_bookmark31)
11. [**CLINICAL PHARMACOLOGY**](#_bookmark32)
    1. [Mechanism of Action](#_bookmark33)
    2. [Pharmacodynamics](#_bookmark34)
    3. [Pharmacokinetics](#_bookmark35)
12. [**NONCLINICAL TOXICOLOGY**](#_bookmark36)
    1. [Carcinogenesis, Mutagenesis, Impairment of Fertility](#_bookmark37)
13. [**CLINICAL STUDIES**](#_bookmark38)
    1. [Updated Adjuvant Treatment of Early Breast Cancer](#_bookmark39)
    2. [Extended Adjuvant Treatment of Early Breast Cancer, Median](#_bookmark40) [Treatment Duration of 24 Months](#_bookmark40)
    3. [Updated Analyses of Extended Adjuvant Treatment of Early](#_bookmark41) [Breast Cancer, Median Treatment Duration of 60 Months](#_bookmark41)
    4. [First-Line Treatment of Advanced Breast Cancer](#_bookmark42)
    5. [Second-Line Treatment of Advanced Breast Cancer](#_bookmark43)
14. [**HOW SUPPLIED/STORAGE AND HANDLING**](#_bookmark44)
15. [**PATIENT COUNSELING INFORMATION**](#_bookmark45)

*Sections or subsections omitted from the full prescribing information are not listed

# FULL PRESCRIBING INFORMATION

1. **INDICATIONS AND USAGE**
   1. **Adjuvant Treatment of Early Breast Cancer**

Femara (letrozole) is indicated for the adjuvant treatment of postmenopausal women with hormone receptor positive early breast cancer.

- 1. **Extended Adjuvant Treatment of Early Breast Cancer**

Femara is indicated for the extended adjuvant treatment of early breast cancer in postmenopausal women, who have received 5 years of adjuvant tamoxifen therapy. The effectiveness of Femara in extended adjuvant treatment of early breast cancer is based on an analysis of disease-free survival in patients treated with Femara for a median of 60 months *[see Clinical Studies (14.2, 14.3)]*.

- 1. **First and Second-Line Treatment of Advanced Breast Cancer**

Femara is indicated for first-line treatment of postmenopausal women with hormone receptor positive or unknown, locally advanced or metastatic breast cancer. Femara is also indicated for the treatment of advanced breast cancer in postmenopausal women with disease progression following antiestrogen therapy *[see Clinical Studies (14.4, 14.5)].*

1. **DOSAGE AND ADMINISTRATION**
   1. **Recommended Dose**

The recommended dose of Femara is one 2.5 mg tablet administered once a day, without regard to meals.

# Use in Adjuvant Treatment of Early Breast Cancer

In the adjuvant setting, the optimal duration of treatment with letrozole is unknown. In both the adjuvant study and the post approval adjuvant study, median treatment duration was 5 years. Treatment should be discontinued at relapse *[see Clinical Studies (14.1)].*

# Use in Extended Adjuvant Treatment of Early Breast Cancer

In the extended adjuvant setting, the optimal treatment duration with Femara is not known. The planned duration of treatment in the study was 5 years. In the final updated analysis, conducted at a median follow-up of 62 months, the median treatment duration for Femara was 60 months. Seventy-one (71%) percent of patients were treated for at least 3 years and 58% of patients completed at least

4.5 years of extended adjuvant treatment. The treatment should be discontinued at tumor relapse *[see Clinical Studies (14.2)].*

# Use in First and Second-Line Treatment of Advanced Breast Cancer

In patients with advanced disease, treatment with Femara should continue until tumor progression is evident *[see Clinical Studies (14.4, 14.5)]*.

# Use in Hepatic Impairment

No dosage adjustment is recommended for patients with mild to moderate hepatic impairment, although Femara blood concentrations were modestly increased in subjects with moderate hepatic impairment due to cirrhosis. The dose of Femara in patients with cirrhosis and severe hepatic dysfunction should be reduced by 50% *[see Warnings and Precautions (5.3)].* The recommended dose of Femara for such patients is 2.5 mg administered every other day. The effect of hepatic impairment on Femara exposure in noncirrhotic cancer patients with elevated bilirubin levels has not been determined.

# Use in Renal Impairment

No dosage adjustment is required for patients with renal impairment if creatinine clearance is greater than or equal to 10 mL/min *[see Clinical Pharmacology (12.3)]*.

# DOSAGE FORMS AND STRENGTHS

2.5 mg tablets: dark yellow, film-coated, round, slightly biconvex, with beveled edges (imprinted with the letters FV on one side and CG on the other side).

# CONTRAINDICATIONS

- Pregnancy: Letrozole can cause fetal harm *[see Use in Specific Populations (8.1)]*.
- Known hypersensitivity to the active substance, or to any of the excipients *[see Adverse Reactions (6)]*.

# WARNINGS AND PRECAUTIONS

- 1. **Bone Effects**

Use of Femara may cause decreases in bone mineral density (BMD). Consideration should be given to monitoring BMD. Results of a safety study to evaluate safety in the adjuvant setting comparing the effect on lumbar spine (L2-L4) BMD of adjuvant treatment with letrozole to that with tamoxifen showed at 24 months a median decrease in lumbar spine BMD of 4.1% in the letrozole arm compared to a median increase of 0.3% in the tamoxifen arm (difference = 4.4%) (*P* <0.0001) *[see Adverse Reactions (6)]*. Updated results from the BMD substudy (MA-17B) in the extended adjuvant setting demonstrated that at 2 years patients receiving letrozole had a median decrease from baseline of 3.8% in hip BMD compared to a median decrease of 2.0% in the placebo group. The changes from baseline in lumbar spine BMD in letrozole and placebo treated groups were not significantly different *[see Adverse Reactions (6)].*

In the adjuvant trial (BIG 1-98) the incidence of bone fractures at any time after randomization was 14.7% for letrozole and 11.4% for tamoxifen at a median follow-up of 96 months. The incidence of osteoporosis was 5.1% for letrozole and 2.7% for tamoxifen *[see Adverse Reactions (6)]*. In the extended adjuvant trial (MA-17), the incidence of bone fractures at any time after randomization was 13.3% for letrozole and 7.8% for placebo at a median follow-up of 62 months. The incidence of new osteoporosis was 14.5% for letrozole and 7.8% for placebo *[see Adverse Reactions (6)].*

# Cholesterol

Consideration should be given to monitoring serum cholesterol. In the adjuvant trial (BIG 1-98), hypercholesterolemia was reported in 52.3% of letrozole patients and 28.6% of tamoxifen patients. Grade 3-4 hypercholesterolemia was reported in 0.4% of letrozole patients and 0.1% of tamoxifen patients. Also in the adjuvant setting, an increase of greater than or equal to 1.5 x upper limit of normal (ULN) in total cholesterol (generally nonfasting) was observed in patients on monotherapy who had baseline total serum cholesterol within the normal range (i.e., less than =1.5 x ULN) in 155/1843 (8.4%) patients on letrozole vs 71/1840 (3.9%) patients on tamoxifen Lipid lowering medications were required for 29% of patients on letrozole and 20% on tamoxifen *[see Adverse Reactions (6)].*

- 1. **Hepatic Impairment**

Subjects with cirrhosis and severe hepatic impairment who were dosed with 2.5 mg of Femara experienced approximately twice the exposure to Femara as healthy volunteers with normal liver function *[see Clinical Pharmacology (12.3)]*. Therefore, a dose reduction is recommended for this patient population. The effect of hepatic impairment on Femara exposure in cancer patients with elevated bilirubin levels has not been determined *[see Dosage and Administration (2.5)].*

# Fatigue and Dizziness

Because fatigue, dizziness, and somnolence have been reported with the use of Femara, caution is advised when driving or using machinery until it is known how the patient reacts to Femara use.

# Laboratory Test Abnormalities

No dose-related effect of Femara on any hematologic or clinical chemistry parameter was evident. Moderate decreases in lymphocyte counts, of uncertain clinical significance, were observed in some patients receiving Femara 2.5 mg. This depression was transient in about half of those affected. Two patients on Femara developed thrombocytopenia; relationship to the study drug was unclear. Patient withdrawal due to laboratory abnormalities, whether related to study treatment or not was infrequent.

# Embryo-Fetal Toxicity

Based on post-marketing reports, findings from animal studies and the mechanism of action, Femara can cause fetal harm and is contraindicated for use in pregnant women. In post-marketing reports, use of letrozole during pregnancy resulted in cases of spontaneous abortions and congenital birth defects. Letrozole caused embryo-fetal toxicities in rats and rabbits at maternal exposures that were below the maximum recommended human dose (MHRD) on a mg/m2 basis. Advise pregnant women of the potential risk to a fetus. Advise females of reproductive potential to use effective contraception during therapy with Femara and for at least 3 weeks after the last dose *[see Adverse Reactions (6.2), Use in Specific Populations (8.1, 8.3) and Clinical Pharmacology (12.1)]*.

# ADVERSE REACTIONS

The following adverse reactions are discussed in greater detail in other sections of the labeling.

- Bone effects *[see Warnings and Precautions (5.1)]*
- Increases in cholesterol *[see Warnings and Precautions (5.2)]*
- Fatigue and Dizziness *[see Warnings and Precautions (5.4)]*

# Clinical Trials Experience

Because clinical trials are conducted under widely varying conditions, adverse reactions rates observed in the clinical trials of a drug cannot be directly compared to rates in the clinical trials of another drug and may not reflect the rates observed in practice.

***Adjuvant Treatment of Early Breast Cancer***

In study, BIG 1-98, the median treatment duration of adjuvant treatment was 60 months and the median duration of follow-up for safety was 96 months for patients receiving Femara and tamoxifen.

Certain adverse reactions were prospectively specified for analysis (see Table 1), based on the known pharmacologic properties and side effect profiles of the two drugs.

Adverse reactions were analyzed irrespective of whether a symptom was present or absent at baseline. Most adverse reactions reported (approximately 75% of patients who reported AEs) were Grade 1 or Grade 2 applying the Common Toxicity Criteria (CTC) Version 2.0/Common Terminology Criteria for Adverse Events (CTCAE), Version 3.0. Table 1 describes adverse reactions (Grades 1-4 and Grades 3-4) irrespective of relationship to study treatment in the adjuvant trial for the monotherapy arms analysis (safety population).

# Table 1: Patients with Adverse Reactions (CTC Grades 1-4,) in the Adjuvant Study – Monotherapy Arms Analysis (Median Follow-up 96 Months; Median Treatment 60 Months)

| **Grades 1-4** | | | |  |  | **Grades 3-4** | |  |
| --- | --- | --- | --- | --- | --- | --- | --- | --- |
|  | **Femara** |  | **Tamoxifen** | | **Femara** |  | **Tamoxifen** | |
| **Adverse Reactions** | **N=2448** |  | **N=2447** |  | **N=2448** |  | **N=2447** | |
|  | **n (%)** |  | **n (%)** |  | **n (%)** |  | **n (%)** | |
| Patients with any adverse reaction | 2309 ( | 94.3) | 2212 | (90.4) | 636 ( | 26.0) | 606 | (24.8) |
| Hypercholesterolemia* | 1280 ( | 52.3) | 700 | (28.6) | 11 | (0.4) | 6 | (0.2) |
| Hot flashes* | 819 ( | 33.5) | 929 | (38.0) | - | - | - | - |
| Arthralgia/arthritis* | 621 ( | 25.4) | 504 | (20.6) | 84 | (3.4) | 50 | (2.0) |
| Bone fractures1 | 361 ( | 14.7) | 280 | (11.4) | - | - | - | - |
| Night sweats* | 356 ( | 14.5) | 426 | (17.4) | - | - | - | - |
| Weight increase* | 317 ( | 12.9) | 378 | (15.4) | 27 | (1.1) | 39 | (1.6) |
| Nausea* | 284 ( | 11.6) | 277 | (11.3) | 6 | (0.2) | 9 | (0.4) |
| Bone fractures**2 | 249 ( | 10.2) | 175 | (7.2) | - | - | - | - |
| Fatigue (lethargy, malaise, asthenia)* | 235 | (9.6) | 250 | (10.2) | 6 | (0.2) | 7 | (0.3) |
| Myalgia* | 221 | (9.0) | 212 | (8.7) | 18 | (0.7) | 14 | (0.6) |
| Vaginal bleeding* | 129 | (5.3) | 320 | (13.1) | 1 ( | <0.1) | 8 | (0.3) |
| Edema* | 164 | (6.7) | 160 | (6.5) | 3 | (0.1) | 1 | (<0.1) |
| Weight decrease | 140 | (5.7) | 129 | (5.3) | 8 | (0.3) | 5 | (0.2) |
| Osteoporosis** | 126 | (5.1) | 67 | (2.7) | 10 | (0.4) | 5 | (0.2) |
| Back pain | 125 | (5.1) | 136 | (5.6) | 7 | (0.3) | 11 | (0.4) |
| Bone pain | 123 | (5.0) | 109 | (4.5) | 6 | (0.2) | 4 | (0.2) |
| Depression | 119 | (4.9) | 114 | (4.7) | 16 | (0.7) | 14 | (0.6) |
| Vaginal irritation* | 112 | (4.6) | 77 | (3.1) | 2 ( | <0.1) | 2 | (<0.1) |
| Headache* | 105 | (4.3) | 94 | (3.8) | 8 | (0.3) | 4 | (0.2) |
| Pain in extremity | 103 | (4.2) | 79 | (3.2) | 6 | (0.2) | 4 | (0.2) |
| Osteopenia* | 87 | (3.6) | 76 | (3.1) | 0 | - | 3 | (0.1) |
| Dizziness/light-headedness* | 84 | (3.4) | 80 | (3.3) | 1 ( | <0.1) | 6 | (0.2) |
| Alopecia | 83 | (3.4) | 84 | (3.4) | - | - | - | - |
| Vomiting* | 80 | (3.3) | 80 | (3.3) | 3 | (0.1) | 5 | (0.2) |
| Cataract* | 49 | (2.0) | 54 | (2.2) | 16 | (0.7) | 17 | (0.7) |
| Constipation* | 49 | (2.0) | 71 | (2.9) | 3 | (0.1) | 1 | (<0.1) |

| Myocardial infarction1 | 42 | (1.7) | 28 | (1.1) | - | - | - | - |
| --- | --- | --- | --- | --- | --- | --- | --- | --- |
| Breast pain* | 37 | (1.5) | 43 | (1.8) | 1 | (<0.1) | - | - |
| Anorexia* | 20 | (0.8) | 20 | (0.8) | 1 | (<0.1) | 1 | (<0.1) |
| Endometrial proliferation disorders* | 14 | (0.6) | 86 | (3.5) | 0 | - | 14 | (0.6) |
| Ovarian cyst* | 11 | (0.4) | 18 | (0.7) | 4 | (0.2) | 4 | (0.2) |
| Endometrial hyperplasia/cancer**1 | 11 | (0.4) | 72 | (2.9) | - | - | - | - |
| Endometrial hyperplasia/cancer**,3 | 6/1909 | (0.3) | 57/1943 | (2.9) | - | - | - | - |
| Other endometrial disorders* | 2 | (<0.1) | 3 | (0.1) | 0 | - | 0 | - |
| Myocardial infarction**2 | 24 | (1.0) | 12 | (0.5) | - | - | - | - |
| Myocardial ischemia | 6 | (0.2) | 9 | (0.4) | - | - | - | - |
| Cerebrovascular accident/TIA**1 | 74 | (3.0) | 68 | (2.8) | - | - | - | - |
| Cerebrovascular accident/TIA**2 | 51 | (2.1) | 47 | (1.9) | - | - | - | - |
| Angina requiring surgery**1 | 35 | (1.4) | 33 | (1.3) | - | - | - | - |
| Angina requiring surgery**2 | 25 | (1.0) | 25 | (1.0) | - | - | - | - |
| Thromboembolic event**1 | 79 | (3.2) | 113 | (4.6) | - | - | - | - |
| Thromboembolic event**2 | 51 | (2.1) | 89 | (3.6) | - | - | - | - |
| Cardiac failure1 | 39 | (1.6) | 34 | (1.4) | - | - | - | - |
| Cardiac failure2 | 27 | (1.1) | 15 | (0.6) | - | - | - | - |
| Hypertension1 | 160 | (6.5) | 175 | (7.2) | - | - | - | - |
| Hypertension2 | 138 | (5.6) | 139 | (5.7) | - | - | - | - |
| Other cardiovascular**1 | 172 | (7.0) | 174 | (7.1) | - | - | - | - |
| Other cardiovascular**2 | 120 | (4.9) | 119 | (4.9) | - | - | - | - |
| Second primary malignancy1 | 129 | (5.3) | 150 | (6.1) | - | - | - | - |
| Second primary malignancy2 | 54 | (2.2) | 79 | (3.2) | - | - | - | - |
| * Target events pre-specified for analysis  ** Events pre-printed on CRF | | | | | | | | |
| 1At median follow-up of 96 months (i.e. any time after randomization) for Femara (range up to 144 months) and 95 months for  tamoxifen (range up to 143 months )  2At median treatment duration of 60 months (i.e. during treatment + 30 days after discontinuation of treatment) for Femara and tamoxifen (range up to 68 months)  3Excluding women who had undergone hysterectomy before study entry TIA = Transient ischemic attack  Note: Cardiovascular events (including cerebrovascular and thromboembolic events), skeletal and urogenital/endometrial events and second primary malignancies were collected life -long. All of these events were assumed to be of CTC Grade 3 to 5 and were not individually graded | | | | | | | | |

When considering all grades during study treatment, a higher incidence of events was seen for Femara regarding fractures (10.1% vs 7.1%), myocardial infarctions (1.0% vs 0.5%), and arthralgia (25.2% vs 20.4%) (Femara vs tamoxifen respectively). A higher incidence was seen for tamoxifen regarding thromboembolic events (2.1% vs 3.6%), endometrial hyperplasia/cancer (0.3% vs 2.9%), and endometrial proliferation disorders (0.3% vs 1.8%) (Femara vs tamoxifen respectively).

At a median follow-up of 96 months, a higher incidence of events was seen for Femara (14.7%) than for tamoxifen (11.4%) regarding fractures. A higher incidence was seen for tamoxifen compared to Femara regarding thromboembolic events (4.6% vs 3.2%), and endometrial hyperplasia or cancer (2.9% vs 0.4%) (tamoxifen vs Femara, respectively).

***Bone Study***: Results of a safety trial in 263 postmenopausal women with resected receptor positive early breast cancer in the adjuvant setting comparing the effect on lumbar spine (L2-L4) BMD of adjuvant treatment with letrozole to that with tamoxifen showed at 24 months a median decrease in lumbar spine BMD of 4.1% in the letrozole arm compared to a median increase of 0.3% in the tamoxifen arm (difference = 4.4%) (*P* <0.0001). No patients with a normal BMD at baseline became osteoporotic over the 2 years and only 1 patient with osteopenia at baseline (T score of -1.9) developed

osteoporosis during the treatment period (assessment by central review). The results for total hip BMD were similar, although the differences between the two treatments were less pronounced. During the 2 year period, fractures were reported by 4 of 103 patients (4%) in the letrozole arm, and 6 of 97 patients (6%) in the tamoxifen arm.

***Lipid Study***: In a safety trial in 263 postmenopausal women with resected receptor positive early breast cancer at 24 months comparing the effects on lipid profiles of adjuvant letrozole to tamoxifen, 12% of patients on letrozole had at least one total cholesterol value of a higher CTCAE grade than at baseline compared with 4% of patients on tamoxifen. In another postapproval randomized, multicenter, open label, study of letrozole vs anastrozole in the adjuvant treatment of postmenopausal women with hormone receptor and node positive breast cancer (FACE, NCT00248170), the median duration of treatment was 60 months for both treatment arms. Table 2 describes adverse reactions (Grades 1-4 and Grades 3-4) irrespective of relationship to study treatment in the adjuvant study (safety population).

**Table 2: Adverse Reactions (CTC Grades 1-4), Occurring in at least 5% of Patients in Either Treatment Arm, by Preferred Term (Safety set)**

| **Adverse Reactions** | **Letrozole N = 2049 n (%)** | | **Anastrozole N = 2062**  **n (%)** | |
| --- | --- | --- | --- | --- |
|  | **Grade 3/4 n (%)** | **All grades n (%)** | **Grade 3/4 n (%)** | **All grades n (%)** |
| Patients with at least one AR | 628 (30.6) | 2049 (100.0) | 591 (28.7) | 2062 (100.0) |
| Arthralgia | 80 (3.9) | 987 (48.2) | 69 (3.3) | 987 (47.9) |
| Hot flush | 17 (0.8) | 666 (32.5) | 9 (0.4) | 666 (32.3) |
| Fatigue | 8 (0.4) | 345 (16.8) | 10 (0.5) | 343 (16.6) |
| Osteoporosis | 5 (0.2) | 223 (10.9) | 11 (0.5) | 225 (10.9) |
| Myalgia | 16 (0.8) | 233 (11.4) | 15 (0.7) | 212 (10.3) |
| Back pain | 11 (0.5) | 212 (10.3) | 17 (0.8) | 193 (9.4) |
| Osteopenia | 4 (0.2) | 203 (9.9) | 1 (0.0) | 173 (8.4) |
| Pain in extremity | 9 (0.4) | 168 (8.2) | 3 (0.1) | 174 (8.4) |
| Lymphoedema | 5 (0.2) | 159 (7.8) | 2 (0.1) | 179 (8.7) |
| Insomnia | 7 (0.3) | 160 (7.8) | 3 (0.1) | 149 (7.2) |
| Hypercholesterolaemia | 2 (0.1) | 155 (7.6) | 1 (0.0) | 151 (7.3) |
| Hypertension | 25 (1.2) | 156 (7.6) | 20 (1.0) | 149 (7.2) |
| Depression | 16 (0.8) | 147 (7.2) | 13 (0.6) | 137 (6.6) |
| Bone pain | 10 (0.5) | 138 (6.7) | 9 (0.4) | 122 (5.9) |
| Nausea | 6 (0.3) | 137 (6.7) | 5 (0.2) | 152 (7.4) |
| Headache | 3 (0.1) | 130 (6.3) | 5 (0.2) | 168 (8.1) |
| Alopecia | 2 (0.1) | 127 (6.2) | 0 (0.0) | 134 (6.5) |
| Musculoskeletal pain | 6 (0.3) | 123 (6.0) | 9 (0.4) | 147 (7.1) |
| Radiation skin injury | 11 (0.5) | 120 (5.9) | 6 (0.3) | 88 (4.3) |
| Dyspnoea | 16 (0.8) | 118 (5.8) | 10 (0.5) | 96 (4.7) |
| Cough | 1 (0.0) | 106 (5.2) | 1 (0.0) | 120 (5.8) |
| Musculoskeletal stiffness | 2 (0.1) | 102 (5.0) | 2 (0.1) | 84 (4.1) |
| Dizziness | **2 (0.2)** | **94 (4.6)** | **7 (0.3)** | **109 (5.3)** |

The following adverse reactions were also identified in less than 5% of the 2049 patients treated with letrozole and not included in the table: fall, vertigo, hyperbilirubinemia, jaundice, and chest pain.

***Extended Adjuvant Treatment of Early Breast Cancer, Median Treatment Duration of 24 Months***

In study MA-17, the median duration of extended adjuvant treatment was 24 months ~~a~~nd the median duration of follow-up for safety was 28 months for patients receiving Femara and placebo.

Table 3 describes the adverse reactions occurring at a frequency of at least 5% in any treatment group during treatment. Most adverse reactions reported were Grade 1 and Grade 2 based on the CTC Version 2.0. In the extended adjuvant setting, the reported drug-related adverse reactions that were significantly different from placebo were hot flashes, arthralgia/arthritis, and myalgia.

**Table 3: Adverse Reactions Occurring in at least 5% of Patients in either Treatment Arm**

|  | **Number (%) of Patients with Grade 1-4 Adverse Reactions** | | **Number (%) of Patients with Grade 3-4 Adverse Reactions** | |
| --- | --- | --- | --- | --- |
|  | **Femara** | **Placebo** | **Femara** | **Placebo** |
|  | **N=2563** | **N=2573** | **N=2563** | **N=2573** |
| **Any Adverse Reactions** | 2232 (87.1) | 2174 (84.5) | 419 (16.3) | 389 (15.1) |
| **Vascular Disorders** | 1375 (53.6) | 1230 (47.8) | 59 (2.3) | 74 (2.9) |
| Flushing | 1273 (49.7) | 1114 (43.3) | 3 (0.1) | 0 |
| **General Disorders** | 1154 (45) | 1090 (42.4) | 30 (1.2) | 28 (1.1) |
| Asthenia | 862 (33.6) | 826 (32.1) | 16 (0.6) | 7 (0.3) |
| Edema NOS | 471 (18.4) | 416 (16.2) | 4 (0.2) | 3 (0.1) |
| **Musculoskeletal Disorders** | 978 (38.2) | 836 (32.5) | 71 (2.8) | 50 (1.9) |
| Arthralgia | 565 (22) | 465 (18.1) | 25 (1) | 20 (0.8) |
| Arthritis NOS | 173 (6.7) | 124 (4.8) | 10 (0.4) | 5 (0.2) |
| Myalgia | 171 (6.7) | 122 (4.7) | 8 (0.3) | 6 (0.2) |
| Back Pain | 129 (5) | 112 (4.4) | 8 (0.3) | 7 (0.3) |
| **Nervous System Disorders** | 863 (33.7) | 819 (31.8) | 65 (2.5) | 58 (2.3) |
| Headache | 516 (20.1) | 508 (19.7) | 18 (0.7) | 17 (0.7) |
| Dizziness | 363 (14.2) | 342 (13.3) | 9 (0.4) | 6 (0.2) |
| **Skin Disorders** | 830 (32.4) | 787 (30.6) | 17 (0.7) | 16 (0.6) |
| Sweating Increased | 619 (24.2) | 577 (22.4) | 1 (<0.1) | 0 |
| **Gastrointestinal Disorders** | 725 (28.3) | 731 (28.4) | 43 (1.7) | 42 (1.6) |
| Constipation | 290 (11.3) | 304 (11.8) | 6 (0.2) | 2 (<0.1) |
| Nausea | 221 (8.6) | 212 (8.2) | 3 (0.1) | 10 (0.4) |
| Diarrhea NOS | 128 (5) | 143 (5.6) | 12 (0.5) | 8 (0.3) |
| **Metabolic Disorders** | 551 (21.5) | 537 (20.9) | 24 (0.9) | 32 (1.2) |
| Hypercholesterolemia | 401 (15.6) | 398 (15.5) | 2 (<0.1) | 5 (0.2) |
| **Reproductive Disorders** | 303 (11.8) | 357 (13.9) | 9 (0.4) | 8 (0.3) |
| Vaginal Hemorrhage | 123 (4.8) | 171 (6.6) | 2 (<0.1) | 5 (0.2) |
| Vulvovaginal Dryness | 137 (5.3) | 127 (4.9) | 0 | 0 |
| **Psychiatric Disorders** | 320 (12.5) | 276 (10.7) | 21 (0.8) | 16 (0.6) |
| Insomnia | 149 (5.8) | 120 (4.7) | 2 (<0.1) | 2 (<0.1) |
| **Respiratory Disorders** | 279 (10.9) | 260 (10.1) | 30 (1.2) | 28 (1.1) |
| Dyspnea | 140 (5.5) | 137 (5.3) | 21 (0.8) | 18 (0.7) |
| **Investigations** | 184 (7.2) | 147 (5.7) | 13 (0.5) | 13 (0.5) |
| **Infections and Infestations** | 166 (6.5) | 163 (6.3) | 40 (1.6) | 33 (1.3) |
| **Renal Disorders** | 130 (5.1) | 100 (3.9) | 12 (0.5) | 6 (0.2) |

Based on a median follow-up of patients for 28 months,the incidence of clinical fractures from the core randomized study in patients who received Femara was 5.9% (152) and placebo was 5.5% (142). The incidence of self-reported osteoporosis was higher in patients who received Femara 6.9% (176) than in patients who received placebo 5.5% (141). Bisphosphonates were administered to 21.1% of the patients who received Femara and 18.7% of the patients who received placebo.

The incidence of cardiovascular ischemic events from the core randomized study was comparable between patients who received Femara 6.8% (175) and placebo 6.5% (167).

A patient-reported measure that captures treatment impact on important symptoms associated with estrogen deficiency demonstrated a difference in favor of placebo for vasomotor and sexual symptom domains.

***Bone Substudy:*** *[see Warnings and Precautions (5.1)]*

***Lipid Substudy:*** In the extended adjuvant setting, based on a median duration of follow-up of 62 months, there was no significant difference between Femara and placebo in total cholesterol or in any lipid fraction at any time over 5 years. Use of lipid lowering drugs or dietary management of elevated lipids was allowed *[see Warnings and Precautions (5.2)].*

## *Updated Analysis, Extended Adjuvant Treatment of Early Breast Cancer, Median Treatment Duration of 60 Months*

The extended adjuvant treatment trial (MA-17) was unblinded early *[see Adverse Reactions (6)]*. At the updated (final analysis), overall the side effects seen were consistent to those seen at a median treatment duration of 24 months.

During treatment or within 30 days of stopping treatment (median duration of treatment 60 months) a higher rate of fractures was observed for Femara (10.4%) compared to placebo (5.8%), as also a higher rate of osteoporosis (Femara 12.2% vs placebo 6.4%).

Based on 62 months median duration of follow-up in the randomized letrozole arm in the safety population the incidence of new fractures at any time after randomization was 13.3% for letrozole and 7.8% for placebo. The incidence of new osteoporosis was 14.5% for letrozole and 7.8% for placebo.

During treatment or within 30 days of stopping treatment (median duration of treatment 60 months), the incidence of cardiovascular events was 9.8% for Femara and 7.0% for placebo.

Based on 62 months median duration of follow-up in the randomized letrozole arm in the safety population the incidence of cardiovascular disease at any time after randomization was 14.4% for letrozole and 9.8% for placebo.

***Lipid substudy***: In the extended adjuvant setting (MA-17), based on a median duration of follow-up of 62 months, there was no significant difference between Femara and placebo in total cholesterol or in any lipid fraction over 5 years. Use of lipid lowering drugs or dietary management of elevated lipids was allowed *[see Warnings and Precautions (5.2)].*

## *First-Line Treatment of Advanced Breast Cancer*

In study P025 a total of 455 patients were treated for a median time of exposure of 11 months in the Femara arm (median 6 months in the tamoxifen arm). The incidence of adverse reactions was similar for Femara and tamoxifen. The most frequently reported adverse reactions were bone pain, hot flushes, back pain, nausea, arthralgia and dyspnea. Discontinuations for adverse reactions other than progression of tumor occurred in 10/455 (2%) of patients on Femara and in 15/455 (3%) of patients on tamoxifen.

Adverse reactions that were reported in at least 5% of the patients treated with Femara 2.5 mg or tamoxifen 20 mg in the first-line treatment study are shown in Table 4.

# Table 4: Adverse Reactions Occurring in at least 5% of Patients in either Treatment Arm

| **Adverse** | **Femara** | **Tamoxifen** |
| --- | --- | --- |
| **Reactions** | **2.5 mg** | **20 mg** |
|  | **(N=455)** | **(N=455)** |
|  | **%** | **%** |
| **General Disorders** |  |  |
| Fatigue | 13 | 13 |
| Chest Pain | 8 | 9 |
| Edema Peripheral | 5 | 6 |
| Pain NOS | 5 | 7 |
| Weakness | 6 | 4 |
| **Investigations** |  |  |
| Weight Decreased | 7 | 5 |
| **Vascular Disorders** |  |  |
| Hot Flushes | 19 | 16 |
| Hypertension | 8 | 4 |
| **Gastrointestinal Disorders** |  |  |
| Nausea | 17 | 17 |
| Constipation | 10 | 11 |
| Diarrhea | 8 | 4 |
| Vomiting | 7 | 8 |
| **Infections/Infestations** |  |  |
| Influenza | 6 | 4 |
| Urinary Tract Infection NOS | 6 | 3 |
| **Injury, Poisoning and Procedural Complications** |  |  |
| Post-Mastectomy Lymphedema | 7 | 7 |
| **Metabolism and Nutrition Disorders** |  |  |
| Anorexia | 4 | 6 |
| **Musculoskeletal and Connective Tissue Disorders** |  |  |
| Bone Pain | 22 | 21 |
| Back Pain | 18 | 19 |
| Arthralgia | 16 | 15 |
| Pain in Limb | 10 | 8 |
| **Nervous System Disorders** |  |  |
| Headache NOS | 8 | 7 |

| **Psychiatric Disorders** |  |  |
| --- | --- | --- |
| Insomnia | 7 | 4 |
| **Reproductive System and Breast Disorders** |  |  |
| Breast Pain | 7 | 7 |
| **Respiratory, Thoracic and Mediastinal Disorders** |  |  |
| Dyspnea | 18 | 17 |
| Cough | 13 | 13 |
| Chest Wall Pain | 6 | 6 |

Other less frequent (less than or equal to 2%) adverse reactions considered consequential for both treatment groups, included peripheral thromboembolic events, cardiovascular events, and cerebrovascular events. Peripheral thromboembolic events included venous thrombosis, thrombophlebitis, portal vein thrombosis and pulmonary embolism. Cardiovascular events included angina, myocardial infarction, myocardial ischemia, and coronary heart disease. Cerebrovascular events included transient ischemic attacks, thrombotic or hemorrhagic strokes and development of hemiparesis.

## *Second-Line Treatment of Advanced Breast Cancer*

Study discontinuations in the megestrol acetate comparison study (AR/BC2) for adverse reactions other than progression of tumor were 5/188 (2.7%) on Femara 0.5 mg, in 4/174 (2.3%) on Femara 2.5 mg, and in 15/190 (7.9%) on megestrol acetate. There were fewer thromboembolic events at both Femara doses than on the megestrol acetate arm (0.6% vs 4.7%). There was also less vaginal bleeding (0.3% vs 3.2%) on Femara than on megestrol acetate. In the aminoglutethimide comparison study (AR/BC3), discontinuations for reasons other than progression occurred in 6/193 (3.1%) on 0.5 mg Femara, 7/185 (3.8%) on 2.5 mg Femara, and 7/178 (3.9%) of patients on aminoglutethimide.

Comparisons of the incidence of adverse reactions revealed no significant differences between the high and low dose Femara groups in either study. Most of the adverse reactions observed in all treatment groups were mild to moderate in severity and it was generally not possible to distinguish adverse reactions due to treatment from the consequences of the patient’s metastatic breast cancer, the effects of estrogen deprivation, or intercurrent illness.

Adverse reactions that were reported in at least 5% of the patients treated with Femara 0.5 mg, Femara 2.5 mg, megestrol acetate, or aminoglutethimide in the two controlled trials AR/BC2 and AR/BC3 are shown in Table 5.

# Table 5: Adverse Reactions Occurring at a Frequency of at Least 5% of Patients in Either Treatment Arm

| **Adverse** | **Pooled** | **Pooled** | **megestrol** |  |
| --- | --- | --- | --- | --- |
| **Reactions** | **Femara** | **Femara** | **acetate** | **aminoglutethimide** |
|  | **2.5 mg** | **0.5 mg** | **160 mg** | **500 mg** |
|  | **(N=359)** | **(N=380)** | **(N=189)** | **(N=178)** |
|  | **%** | **%** | **%** | **%** |
| **Body as a Whole** |  |  |  |  |
| Chest Pain | 6 | 3 | 7 | 3 |
| Peripheral Edema1 | 5 | 5 | 8 | 3 |
| Asthenia | 4 | 5 | 4 | 5 |
| Weight Increase | 2 | 2 | 9 | 3 |
| **Cardiovascular** |  |  |  |  |
| Hypertension | 5 | 7 | 5 | 6 |
| **Digestive System** |  |  |  |  |
| Nausea | 13 | 15 | 9 | 14 |
| Vomiting | 7 | 7 | 5 | 9 |
| Constipation | 6 | 7 | 9 | 7 |
| Diarrhea | 6 | 5 | 3 | 4 |
| Pain-Abdominal | 6 | 5 | 9 | 8 |
| Anorexia | 5 | 3 | 5 | 5 |
| Dyspepsia | 3 | 4 | 6 | 5 |
| **Infections/Infestations** |  |  |  |  |
| Viral Infection | 6 | 5 | 6 | 3 |
| **Lab Abnormality** |  |  |  |  |
| Hypercholesterolemia | 3 | 3 | 0 | 6 |
| **Musculoskeletal System** |  |  |  |  |
| Musculoskeletal2 | 21 | 22 | 30 | 14 |
| Arthralgia | 8 | 8 | 8 | 3 |
| **Nervous System** |  |  |  |  |

| Headache | 9 | 12 | 9 | 7 |
| --- | --- | --- | --- | --- |
| Somnolence | 3 | 2 | 2 | 9 |
| Dizziness | 3 | 5 | 7 | 3 |
| **Respiratory System** |  |  |  |  |
| Dyspnea | 7 | 9 | 16 | 5 |
| Coughing | 6 | 5 | 7 | 5 |
| **Skin and Appendages** |  |  |  |  |
| Hot Flushes | 6 | 5 | 4 | 3 |
| Rash3 | 5 | 4 | 3 | 12 |
| Pruritus | 1 | 2 | 5 | 3 |

1Includes peripheral edema, leg edema, dependent edema, edema

2Includes musculoskeletal pain, skeletal pain, back pain, arm pain, leg pain

3Includes rash, erythematous rash, maculopapular rash, psoriasiform rash, vesicular rash

Other less frequent (less than 5%) adverse reactions considered consequential and reported in at least 3 patients treated with Femara, included hypercalcemia, fracture, depression, anxiety, pleural effusion, alopecia, increased sweating and vertigo.

## *First and Second-Line Treatment of Advanced Breast Cancer*

In the combined analysis of the first- and second-line metastatic trials and postmarketing experiences other adverse reactions that were reported were cataract, eye irritation, palpitations, cardiac failure, tachycardia, dysesthesia (including hypesthesia/paresthesia), arterial thrombosis, memory impairment, irritability, nervousness, urticaria, increased urinary frequency, leukopenia, stomatitis cancer pain, pyrexia, vaginal discharge, appetite increase, dryness of skin and mucosa (including dry mouth), and disturbances of taste and thirst.

# Postmarketing Experience

The following adverse reactions have been identified during postapproval use of Femara. Because these reactions are reported voluntarily from a population of uncertain size, it is not always possible to reliably estimate their frequency or establish a causal relationship to drug exposure.

- - - Eye Disorders: blurred vision
    - Hepatobiliary Disorders: increased hepatic enzymes, hepatitis
    - Immune System Disorders: anaphylactic reactions, hypersensitivity reactions
    - Nervous System Disorders: carpal tunnel syndrome, trigger finger
    - Pregnancy: spontaneous abortions, congenital birth defects
    - Skin and subcutaneous disorders: angioedema, toxic epidermal necrolysis, erythema multiforme

# DRUG INTERACTIONS

#### *Tamoxifen*

Coadministration of Femara and tamoxifen 20 mg daily resulted in a reduction of letrozole plasma levels of 38% on average (study P015). Clinical experience in the second-line breast cancer trials (AR/BC2 and AR/BC3) indicates that the therapeutic effect of Femara therapy is not impaired if Femara is administered immediately after tamoxifen.

#### *Cimetidine*

A pharmacokinetic interaction study with cimetidine (study P004) showed no clinically significant effect on letrozole pharmacokinetics.

#### *Warfarin*

An interaction study (P017) with warfarin showed no clinically significant effect of letrozole on warfarin pharmacokinetics.

#### *Other anticancer agents*

There is no clinical experience to date on the use of Femara in combination with other anticancer agents.

# USE IN SPECIFIC POPULATIONS

- 1. **Pregnancy**

Risk Summary

Based on postmarketing reports, findings from animal studies and the mechanism of action, Femara can cause fetal harm and is contraindicated for use in pregnant women. In post-marketing reports, use of letrozole during pregnancy resulted in cases of spontaneous abortions and congenital birth defects; however, the data are insufficient to inform a drug-associated risk *[see Contraindications (4), Warnings and Precautions (5.6), Adverse Reactions (6.2), and Clinical Pharmacology (12.1)]*.

In animal reproduction studies, administration of letrozole to pregnant animals during organogenesis resulted in increased post- implantation pregnancy loss and resorption, fewer live fetuses, and fetal malformation affecting the renal and skeletal systems in rats and rabbits at doses approximately 0.1 times the daily maximum recommended human dose (MRHD) on a mg/m2 basis *(see Data)*.

The background risk of major birth defects and miscarriage for the indicated population is unknown. However, the background risk in the U.S. general population of major birth defects is 2%-4% and of miscarriage is 15%-20% of clinically recognized pregnancies.

Data

*Animal Data*

In a fertility and early embryonic development toxicity study in female rats, oral administration of letrozole starting 2 weeks before mating until pregnancy day 6 resulted in an increase in pre-implantation loss at doses ≥ 0.003 mg/kg/day (approximately 0.01 times the maximum recommended human dose on a mg/m2 basis).

In an embryo-fetal developmental toxicity study in rats, daily administration of oral letrozole during the period of organogenesis at doses ≥ 0.003 mg/kg (approximately 0.01 time the maximum recommended human dose on a mg/m2 basis) resulted in embryo-fetal toxicity including intrauterine mortality, increased resorptions and postimplantation loss, decreased numbers of live fetuses and fetal anomalies including absence and shortening of renal papilla, dilation of ureter, edema and incomplete ossification of frontal skull and metatarsals. Letrozole was teratogenic to rats at a dose of 0.03 mg/kg (approximately 0.01 times the maximum recommended human dose on a mg/m2 basis) and caused fetal domed head and cervical/centrum vertebral fusion.

In the embryo-fetal development toxicity study in rabbits, daily administration of oral letrozole during the period of organogenesis at doses ≥ 0.002 mg/kg (approximately 0.01 times the maximum recommended human dose on a mg/m2 basis) resulted in embryo-fetal toxicity including intrauterine mortality, increased resorption, increased postimplantation loss and decreased numbers of live fetuses. Fetal anomalies included incomplete ossification of the skull, sternebrae, and fore- and hind legs.

# Lactation

Risk Summary

It is not known if letrozole is present in human milk. There are no data on the effects of letrozole on the breastfed infant or milk production. Exposure of lactating rats to letrozole was associated with impaired reproductive performance of the male offspring *(see Data)*. Because of the potential for serious adverse reactions in breastfed infants from Femara, advise lactating women not to breastfeed while taking Femara and for at least 3 weeks after the last dose.

Data

*Animal Data*

In a postnatal developmental toxicity study in lactating rats, letrozole was administered orally at doses of 1, 0.003, 0.03 or 0.3 mg/kg/day on day 0 through day 20 of lactation. The reproductive performance of the male offspring was impaired at letrozole dose as low as 0.003 mg/kg/day (approximately 0.01 times the maximum recommended human dose on a mg/m2 basis), as reflected by decreased mating and pregnancy ratios. There were no effects on the reproductive performance of female offspring.

# Females and Males of Reproductive Potential

Pregnancy Testing

Based on animal studies, Femara can cause fetal harm when administered to a pregnant woman *[see Use in Specific Populations (8.1)]*. Females of reproductive potential should have a pregnancy test prior to starting treatment with Femara.

Contraception

*Females*

Based on animal studies, Femara can cause fetal harm when administered to a pregnant woman *[see Use in Specific Populations (8.1)]*. Advise females of reproductive potential to use effective contraception during treatment with Femara and for at least 3 weeks after the last dose.

Infertility

*Females*

Based on studies in female animals, Femara may impair fertility in females of reproductive potential *[see Nonclinical Toxicology (13.1)]*.

*Males*

Based on studies in male animals, Femara may impair fertility in males of reproductive potential *[see Nonclinical Toxicology (13.1)]*.

# Pediatric Use

The safety and effectiveness in pediatric patients have not been established.

Letrozole administration to young (postnatal day 7) rats for 12 weeks duration at 0.003, 0.03, 0.3 mg/kg/day by oral gavage resulted in adverse skeletal/growth effects (bone maturation, bone mineral density) and neuroendocrine and reproductive developmental perturbations of the hypothalamic-pituitary axis. Administration of 0.3 mg/kg/day resulted in AUC values that were similar to the AUC in adult patients receiving the recommended dose of 2.5 mg/day. Decreased fertility was accompanied by hypertrophy of the hypophysis and testicular changes that included degeneration of the seminiferous tubular epithelium and atrophy of the female reproductive tract. Young rats in this study were allowed to recover following discontinuation of letrozole treatment for 42 days.

Histopathological changes were not reversible at clinically relevant exposures.

# Geriatric Use

The median age of patients in all studies of first-line and second-line treatment of metastatic breast cancer was 64-65 years. About 1/3 of the patients were greater than or equal to 70 years old. In the first-line study, patients greater than or equal to 70 years of age experienced longer time to tumor progression and higher response rates than patients less than 70.

For the extended adjuvant setting (MA-17), more than 5,100 postmenopausal women were enrolled in the clinical study. In total, 41% of patients were aged 65 years or older at enrollment, while 12% were 75 or older. In the extended adjuvant setting, no overall differences in safety or efficacy were observed between these older patients and younger patients, and other reported clinical experience has not identified differences in responses between the elderly and younger patients, but greater sensitivity of some older individuals cannot be ruled out.

In the adjuvant setting (BIG 1-98), more than 8,000 postmenopausal women were enrolled in the clinical study. In total, 36% of patients were aged 65 years or older at enrollment, while 12% were 75 or older. More adverse reactions were generally reported in elderly patients irrespective of study treatment allocation. However, in comparison to tamoxifen, no overall differences with regards to the safety and efficacy profiles were observed between elderly patients and younger patients.

# OVERDOSAGE

Isolated cases of Femara overdose have been reported. In these instances, the highest single dose ingested was 62.5 mg or 25 tablets. While no serious adverse reactions were reported in these cases, because of the limited data available, no firm recommendations for treatment can be made. However, emesis could be induced if the patient is alert. In general, supportive care and frequent monitoring of vital signs are also appropriate. In single-dose studies, the highest dose used was 30 mg, which was well tolerated; in multiple-dose trials, the largest dose of 10 mg was well tolerated.

Lethality was observed in mice and rats following single oral doses that were equal to or greater than 2,000 mg/kg (about 4,000 to 8,000 times the daily maximum recommended human dose on a mg/m2 basis); death was associated with reduced motor activity, ataxia and dyspnea. Lethality was observed in cats following single IV doses that were equal to or greater than 10 mg/kg (about 50 times the daily maximum recommended human dose on a mg/m2 basis); death was preceded by depressed blood pressure and arrhythmias.

# DESCRIPTION

Femara tablets for oral administration contains 2.5 mg of letrozole, a nonsteroidal aromatase inhibitor (inhibitor of estrogen synthesis). It is chemically described as 4, 4’-(1H-1, 2, 4-Triazol-1-ylmethylene) dibenzonitrile, and its structural formula is


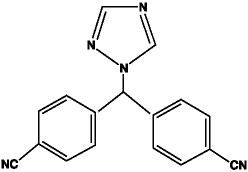


Letrozole is a white to yellowish crystalline powder, practically odorless, freely soluble in dichloromethane, slightly soluble in ethanol, and practically insoluble in water. It has a molecular weight of 285.31, empirical formula C17H11N5, and a melting range of 184°C to 185°C.

Femara is available as 2.5 mg tablets for oral administration.

*Inactive Ingredients:* Colloidal silicon dioxide, ferric oxide, hydroxypropyl methylcellulose, lactose monohydrate, magnesium stearate, maize starch, microcrystalline cellulose, polyethylene glycol, sodium starch glycolate, talc, and titanium dioxide.

# CLINICAL PHARMACOLOGY

- 1. **Mechanism of Action**

The growth of some cancers of the breast is stimulated or maintained by estrogens. Treatment of breast cancer thought to be hormonally responsive (i.e., estrogen and/or progesterone receptor positive or receptor unknown) has included a variety of efforts to decrease estrogen levels (ovariectomy, adrenalectomy, hypophysectomy) or inhibit estrogen effects (antiestrogens and progestational agents). These interventions lead to decreased tumor mass or delayed progression of tumor growth in some women.

In postmenopausal women, estrogens are mainly derived from the action of the aromatase enzyme, which converts adrenal androgens (primarily androstenedione and testosterone) to estrone and estradiol. The suppression of estrogen biosynthesis in peripheral tissues and in the cancer tissue itself can therefore be achieved by specifically inhibiting the aromatase enzyme.

Letrozole is a nonsteroidal competitive inhibitor of the aromatase enzyme system; it inhibits the conversion of androgens to estrogens. In adult nontumor- and tumor-bearing female animals, letrozole is as effective as ovariectomy in reducing uterine weight, elevating serum LH, and causing the regression of estrogen-dependent tumors. In contrast to ovariectomy, treatment with letrozole does not lead to an increase in serum FSH. Letrozole selectively inhibits gonadal steroidogenesis but has no significant effect on adrenal mineralocorticoid or glucocorticoid synthesis.

Letrozole inhibits the aromatase enzyme by competitively binding to the heme of the cytochrome P450 subunit of the enzyme, resulting in a reduction of estrogen biosynthesis in all tissues. Treatment of women with letrozole significantly lowers serum estrone, estradiol and estrone sulfate and has not been shown to significantly affect adrenal corticosteroid synthesis, aldosterone synthesis, or synthesis of thyroid hormones.

# Pharmacodynamics

In postmenopausal patients with advanced breast cancer, daily doses of 0.1 mg to 5 mg Femara (letrozole) suppress plasma concentrations of estradiol, estrone, and estrone sulfate by 75% to 95% from baseline with maximal suppression achieved within two- three days. Suppression is dose-related, with doses of 0.5 mg and higher giving many values of estrone and estrone sulfate that were below the limit of detection in the assays. Estrogen suppression was maintained throughout treatment in all patients treated at 0.5 mg or higher.

Letrozole is highly specific in inhibiting aromatase activity. There is no impairment of adrenal steroidogenesis. No clinically-relevant changes were found in the plasma concentrations of cortisol, aldosterone, 11-deoxycortisol, 17-hydroxy-progesterone, ACTH or in plasma renin activity among postmenopausal patients treated with a daily dose of Femara 0.1 mg to 5 mg. The ACTH stimulation test performed after 6 and 12 weeks of treatment with daily doses of 0.1, 0.25, 0.5, 1, 2.5, and 5 mg did not indicate any attenuation of aldosterone or cortisol production. Glucocorticoid or mineralocorticoid supplementation is, therefore, not necessary.

No changes were noted in plasma concentrations of androgens (androstenedione and testosterone) among healthy postmenopausal women after 0.1, 0.5, and 2.5 mg single doses of Femara or in plasma concentrations of androstenedione among postmenopausal patients treated with daily doses of 0.1 mg to 5 mg. This indicates that the blockade of estrogen biosynthesis does not lead to accumulation of androgenic precursors. Plasma levels of LH and FSH were not affected by letrozole in patients, nor was thyroid function as evaluated by TSH levels, T3 uptake, and T4 levels.

#

***Absorption and Distribution:*** Letrozole is rapidly and completely absorbed from the gastrointestinal tract and absorption is not affected by food. It is metabolized slowly to an inactive metabolite whose glucuronide conjugate is excreted renally, representing the major clearance pathway. About 90% of radiolabeled letrozole is recovered in urine. Letrozole’s terminal elimination half-life is about 2 days and steady-state plasma concentration after daily 2.5 mg dosing is reached in 2-6 weeks. Plasma concentrations at steady state are 1.5 to 2 times higher than predicted from the concentrations measured after a single dose, indicating a slight non-linearity in the pharmacokinetics of letrozole upon daily administration of 2.5 mg. These steady-state levels are maintained over extended periods, however, and continuous accumulation of letrozole does not occur. Letrozole is weakly protein bound and has a large volume of distribution (approximately 1.9 L/kg).

Elimination

***Metabolism and Excretion:*** Metabolism to a pharmacologically-inactive carbinol metabolite (4,4'-methanol-bisbenzonitrile) and renal excretion of the glucuronide conjugate of this metabolite is the major pathway of letrozole clearance. Of the radiolabel recovered in urine, at least 75% was the glucuronide of the carbinol metabolite, about 9% was two unidentified metabolites, and 6% was unchanged letrozole.

In human microsomes with specific CYP isozyme activity, CYP3A4 metabolized letrozole to the carbinol metabolite while CYP2A6 formed both this metabolite and its ketone analog. In human liver microsomes, letrozole inhibited CYP2A6 and CYP2C19, however, the clinical significance of these findings is unknown.

Specific Populations

***Pediatric, Geriatric and Race:*** In the study populations (adults ranging in age from 35 to greater than 80 years), no change in pharmacokinetic parameters was observed with increasing age. Differences in letrozole pharmacokinetics between adult and pediatric populations have not been studied. Differences in letrozole pharmacokinetics due to race have not been studied.

***Renal Impairment:*** In a study of volunteers with varying renal function (24-hour creatinine clearance: 9 to 116 mL/min), no effect of renal function on the pharmacokinetics of single doses of 2.5 mg of Femara was found. In addition, in a study (AR/BC2) of 347 patients with advanced breast cancer, about half of whom received 2.5 mg Femara and half 0.5 mg Femara, renal impairment (calculated creatinine clearance: 20 to 50 mL/min) did not affect steady-state plasma letrozole concentrations.

***Hepatic Impairment:*** In a study of subjects with mild to moderate non-metastatic hepatic dysfunction (e.g., cirrhosis, Child-Pugh classification A and B), the mean area under curve (AUC) values of the volunteers with moderate hepatic impairment were 37% higher than in normal subjects, but still within the range seen in subjects without impaired function.

In a pharmacokinetic study, subjects with liver cirrhosis and severe hepatic impairment (Child-Pugh classification C, which included bilirubins about 2-11 times ULN with minimal to severe ascites) had two-fold increase in exposure (AUC) and 47% reduction in systemic clearance. Breast cancer patients with severe hepatic impairment are thus expected to be exposed to higher levels of letrozole than patients with normal liver function receiving similar doses of this drug *[see Dosage and Administration (2.5)].*

# NONCLINICAL TOXICOLOGY

- 1. **Carcinogenesis, Mutagenesis, Impairment of Fertility**

A conventional carcinogenesis study in mice at doses of 0.6 to 60 mg/kg/day (about 1 to 100 times the daily maximum recommended human dose on a mg/m2 basis) administered by oral gavage for up to 2 years revealed a dose-related increase in the incidence of benign ovarian stromal tumors. The incidence of combined hepatocellular adenoma and carcinoma showed a significant trend in females when the high dose group was excluded due to low survival. In a separate study, plasma AUC0-12hr levels in mice at 60 mg/kg/day were 55 times higher than the AUC0-24hr level in breast cancer patients at the recommended dose. The carcinogenicity study in rats at oral doses of 0.1 to 10 mg/kg/day (about 0.4 to 40 times the daily maximum recommended human dose on a mg/m2 basis) for up to 2 years also produced an increase in the incidence of benign ovarian stromal tumors at 10 mg/kg/day. Ovarian hyperplasia was observed in females at doses equal to or greater than 0.1 mg/kg/day. At 10 mg/kg/day, plasma AUC0-24hr levels in rats were 80 times higher than the level in breast cancer patients at the recommended dose. The benign ovarian stromal tumors observed in mice and rats were considered to be related to the pharmacological inhibition of estrogen synthesis and may be due to increased luteinizing hormone resulting from the decrease in circulating estrogen.

Femara (letrozole) was not mutagenic in *in vitro* tests (Ames and E.coli bacterial tests) but was observed to be a potential clastogen in

*in vitro* assays (CHO K1 and CCL 61 Chinese hamster ovary cells). Letrozole was not clastogenic *in vivo* (micronucleus test in rats).

In a fertility and early embryonic development toxicity study in female rats, oral administration of letrozole starting 2 weeks before mating until pregnancy day 6 resulted in an increase in pre-implantation loss at doses ≥ 0.03 mg/kg/day (approximately 0.1 times the maximum recommended human dose on a mg/m2 basis). In repeat-dose toxicity studies, administration of letrozole caused sexual

inactivity in females and atrophy of the reproductive tract in males and females at doses of 0.6, 0.1 and 0.03 mg/kg in mice, rats and dogs, respectively (approximately 1, 0.4 and 0.4 times the daily maximum recommended human dose on a mg/m2 basis, respectively).

# CLINICAL STUDIES

- 1. **Updated Adjuvant Treatment of Early Breast Cancer**

In a multicenter study (BIG 1-98, NCT00004205) enrolling over 8,000 postmenopausal women with resected, receptor- positive early breast cancer, one of the following treatments was randomized in a double-blind manner:

Option 1:

- - 1. tamoxifen for 5 years
    2. Femara for 5 years
    3. tamoxifen for 2 years followed by Femara for 3 years
    4. Femara for 2 years followed by tamoxifen for 3 years Option 2:

1. tamoxifen for 5 years
2. Femara for 5 years

The study in the adjuvant setting, BIG 1-98 was designed to answer two primary questions: whether Femara for 5 years was superior to Tamoxifen for 5 years (Primary Core Analysis) and whether switching endocrine treatments at 2 years was superior to continuing the same agent for a total of 5 years (Sequential Treatments Analysis). Selected baseline characteristics for the study population are shown in Table 6.

The primary endpoint of this trial was disease-free survival (DFS) (i.e., interval between randomization and earliest occurrence of a local, regional, or distant recurrence, or invasive contralateral breast cancer, or death from any cause). The secondary endpoints were overall survival (OS), systemic disease-free survival (SDFS), invasive contralateral breast cancer, time to breast cancer recurrence (TBR) and time to distant metastasis (TDM).

The Primary Core Analysis (PCA) included all patients and all follow-up in the monotherapy arms in both randomization options, but follow-up in the two sequential treatments arms was truncated 30 days after switching treatments. The PCA was conducted at a median treatment duration of 24 months and a median follow-up of 26 months. Femara was superior to tamoxifen in all endpoints except overall survival and contralateral breast cancer [e.g., DFS: hazard ratio, HR 0.79; 95% CI (0.68, 0.92); *P*=0.002; SDFS: HR 0.83; 95% CI (0.70, 0.97); TDM: HR 0.73; 95% CI (0.60, 0.88); OS: HR 0.86; 95% CI (0.70, 1.06).

In 2005, based on recommendations by the independent Data Monitoring Committee, the tamoxifen arms were unblinded and patients were allowed to complete initial adjuvant therapy with Femara (if they had received tamoxifen for at least 2 years) or to start extended adjuvant treatment with Femara (if they had received tamoxifen for at least 4.5 years) if they remained alive and disease-free. In total, 632 patients crossed to Femara or another aromatase inhibitor. Approximately 70% (448) of these 632 patients crossed to Femara to complete initial adjuvant therapy and most of these crossed in years 3 to 4. All of these patients were in Option 1. A total of 184 patients started extended adjuvant therapy with Femara (172 patients) or with another aromatase inhibitor (12 patients). To explore the impact of this selective crossover, results from analyses censoring follow-up at the date of the selective crossover (in the tamoxifen arm) are presented for the MAA.

The PCA allowed the results of Femara for 5 years compared with tamoxifen for 5 years to be reported in 2005 after a median follow- up of only 26 months. The design of the PCA is not optimal to evaluate the effect of Femara after a longer time (because follow-up was truncated in two arms at around 25 months). The MAA (ignoring the two sequential treatment arms) provided follow-up equally as long in each treatment and did not over-emphasize early recurrences as the PCA did. The MAA thus provides the clinically appropriate updated efficacy results in answer to the first primary question, despite the confounding of the tamoxifen reference arm by the selective crossover to Femara. The updated results for the MAA are summarized in Table 7. Median follow-up for this analysis is 73 months.

The Sequential Treatments Analysis (STA) addresses the second primary question of the study. The primary analysis for the STA was from switch (or equivalent time-point in monotherapy arms) + 30 days (STA-S) with a two-sided test applied to each pair-wise comparison at the 2.5% level. Additional analyses were conducted from randomization (STA-R) but these comparisons (added in light of changing medical practice) were under-powered for efficacy.

**Table 6: Adjuvant Study - Patient and Disease Characteristics (ITT Population)**

| **Primary Core Analysis (PCA)** | | | **Monotherapy Arms Analysis (MAA)** | |
| --- | --- | --- | --- | --- |
|  | **Femara** | **Tamoxifen** | **Femara** | **Tamoxifen** |
|  | **N=4003** | **N=4007** | **N=2463** | **N=2459** |
| **Characteristic** | **n (%)** | **n (%)** | **n (%)** | **n (%)** |
| Age (median, years) | 61 | 61 | 61 | 61 |
| Age range (years) | 38-89 | 39-90 | 38-88 | 39-90 |
| Hormone receptor status (%) |  |  |  |  |
| ER+ and/or PgR+ | 99.7 | 99.7 | 99.7 | 99.7 |
| Both unknown | 0.3 | 0.3 | 0.3 | 0.3 |
| Nodal status (%) |  |  |  |  |
| Node negative | 52 | 52 | 50 | 52 |
| Node positive | 41 | 41 | 43 | 41 |
| Nodal status unknown | 7 | 7 | 7 | 7 |
| Prior adjuvant chemotherapy (%) | 24 | 24 | 24 | 24 |

**Table 7: Updated Adjuvant Study Results - Monotherapy Arms Analysis (Median Follow-up 73 Months)**

|  |  | **Femara N=2463** |  | **Tamoxifen N=2459** | | **Hazard ratio** |  |
| --- | --- | --- | --- | --- | --- | --- | --- |
|  |  | **Events (%)** | **5-year rate** | **Events (%)** | **5-year rate** | **(95% CI)** | ***P*** |
| Disease-free survival1 | ITT | 445 (18.1) | 87.4 | 500 (20.3) | 84.7 | 0.87 (0.76, 0.99) | 0.03 |
|  | Censor | 445 | 87.4 | 483 | 84.2 | 0.84 (0.73, 0.95) |  |
| 0 positive nodes | ITT | 165 | 92.2 | 189 | 90.3 | 0.88 (0.72, 1.09) |  |
| 1-3 positive nodes | ITT | 151 | 85.6 | 163 | 83.0 | 0.85 (0.68, 1.06) |  |
| >=4 positive nodes | ITT | 123 | 71.2 | 142 | 62.6 | 0.81 (0.64, 1.03) |  |
| Adjuvant chemotherapy | ITT | 119 | 86.4 | 150 | 80.6 | 0.77 (0.60, 0.98) |  |
| No chemotherapy | ITT | 326 | 87.8 | 350 | 86.1 | 0.91 (0.78, 1.06) |  |
| Systemic DFS2 | ITT | 401 | 88.5 | 446 | 86.6 | 0.88 (0.77,1.01) |  |
| Time to distant metastasis3 | ITT | 257 | 92.4 | 298 | 90.1 | 0.85 (0.72, 1.00) |  |
| Adjuvant chemotherapy | ITT | 84 | - | 109 | - | 0.75 (0.56-1.00) |  |
| No chemotherapy | ITT | 173 | - | 189 | - | 0.90 (0.73,1.11) |  |
| Distant DFS4 | ITT | 385 | 89.0 | 432 | 87.1 | 0.87 (0.76,1.00) |  |
| Contralateral breast cancer | ITT | 34 | 99.2 | 44 | 98.6 | 0.76 (0.49, 1.19) |  |
| Overall survival | ITT | 303 | 91.8 | 343 | 90.9 | 0.87 (0.75, 1.02) |  |
|  | Censor | 303 | 91.8 | 338 | 90.1 | 0.82 (0.70, 0.96) |  |
| 0 positive nodes | ITT | 107 | 95.2 | 121 | 94.8 | 0.90 (0.69.1.16) |  |
| 1-3 positive nodes | ITT | 99 | 90.8 | 114 | 90.6 | 0.81(0.62,1.06) |  |
| >=4 positive nodes | ITT | 92 | 80.2 | 104 | 73.6 | 0.86 (0.65, 1.14) |  |
| Adjuvant chemotherapy | ITT | 76 | 91.5 | 96 | 88.4 | 0.79 (0.58, 1.06) |  |
| No chemotherapy | ITT | 227 | 91.9 | 247 | 91.8 | 0.91 (0.76, 1.08) |  |
| Definition of:  1Disease-free survival: Interval from randomization to earliest event of invasive loco-regional recurrence, distant metastasis, invasive contralateral breast cancer, or death without a prior event.  2Systemic disease-free survival: Interval from randomization to invasive regional recurrence, distant metastasis, or death without a prior cancer event.  3Time to distant metastasis: Interval from randomization to distant metastasis.  4Distant disease-free survival: Interval from randomization to earlier event of relapse in a distant site or death from any cause. ITT analysis ignores selective crossover in tamoxifen arms.  Censored analysis censors follow-up at the date of selective crossover in 632 patients who crossed to Femara or another aromatase inhibitor after the tamoxifen arms were unblinded in 2005. | | | | | | | |

Figure 1 shows the Kaplan-Meier curves for Disease-Free Survival Monotherapy Analysis


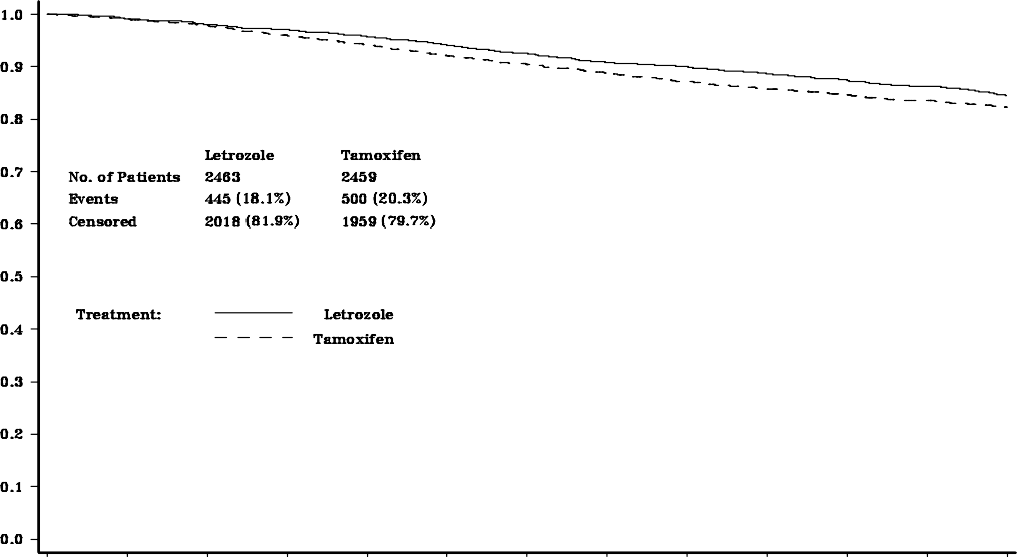
**Figure 1: Disease-Free Survival (Median follow-up 73 months, ITT Approach)**


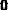

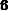

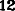

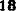

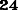

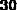

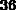

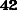

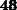

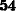

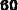

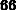

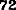


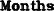


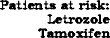

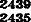

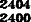

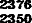

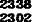

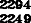

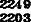

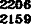

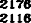

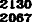

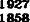

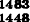

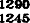


DFS events defined as loco-regional recurrence, distant metastasis, invasive contralateral breast cancer, or death from any cause (i.e., definition excludes second non-breast primary cancers).

The medians of overall survival for both arms were not reached for the MAA. There was no statistically significant difference in overall survival. The hazard ratio for survival in the Femara arm compared to the tamoxifen arm was 0.87, with 95% CI (0.75, 1.02) (see Table 7).

There were no significant differences in DFS, OS, SDFS, and Distant DFS from switch in the Sequential Treatments Analysis with respect to either monotherapy (e.g., [tamoxifen 2 years followed by] Femara 3 years versus tamoxifen beyond 2 years, DFS HR 0.89; 97.5% CI 0.68, 1.15 and [Femara 2 years followed by] tamoxifen 3 years versus Femara beyond 2 years, DFS HR 0.93; 97.5% CI

0.71, 1.22).

There were no significant differences in DFS, OS, SDFS, and Distant DFS from randomization in the Sequential Treatments Analyses.

# Extended Adjuvant Treatment of Early Breast Cancer, Median Treatment Duration of 24 Months

A double-blind, randomized, placebo-controlled trial (MA-17, NCT00003140) of Femara was performed in over 5,100 postmenopausal women with receptor-positive or unknown primary breast cancer who were disease free after 5 years of adjuvant treatment with tamoxifen.

The planned duration of treatment for patients in the study was 5 years, but the trial was terminated early because of an interim analysis showing a favorable Femara effect on time without recurrence or contralateral breast cancer. At the time of unblinding, women had been followed for a median of 28 months, 30% of patients had completed 3 or more years of follow-up and less than 1% of patients had completed 5 years of follow-up.

Selected baseline characteristics for the study population are shown in Table 8.

# Table 8: Selected Study Population Demographics (Modified ITT Population)

| **Baseline Status** | **Femara** | **Placebo** |
| --- | --- | --- |
|  | **N=2582** | **N=2586** |
| **Hormone Receptor Status (%)** |  |  |
| ER+ and/or PgR+ | 98 | 98 |
| Both Unknown | 2 | 2 |
| **Nodal Status (%)** |  |  |

| Node Negative | 50 | 50 |
| --- | --- | --- |
| Node Positive | 46 | 46 |
| Nodal Status Unknown | 4 | 4 |
| **Chemotherapy** | 46 | 46 |

Table 9 shows the study results. Disease-free survival was measured as the time from randomization to the earliest event of loco- regional or distant recurrence of the primary disease or development of contralateral breast cancer or death. DFS by hormone receptor status, nodal status and adjuvant chemotherapy were similar to the overall results. Data were premature for an analysis of survival.

# Table 9: Extended Adjuvant Study Results

|  | **Femara**  **N = 2582** | **Placebo**  **N = 2586** | **Hazard Ratio**  **(95% CI)** | ***P*-Value** |
| --- | --- | --- | --- | --- |
| **Disease Free Survival (DFS)1 Events** | 122 (4.7%) | 193 (7.5%) | 0.62 (0.49, 0.78)2 | 0.00003 |
| Local Breast Recurrence | 9 | 22 |  |  |
| Local Chest Wall Recurrence | 2 | 8 |  |  |
| Regional Recurrence | 7 | 4 |  |  |
| Distant Recurrence | 55 | 92 | 0.61 (0.44 - 0.84) | 0.003 |
| Contralateral Breast Cancer | 19 | 29 |  |  |
| Deaths Without Recurrence or Contralateral Breast Cancer | 30 | 38 |  |  |
| CI = confidence interval for hazard ratio. Hazard ratio of less than 1.0 indicates difference in favor of Femara (lesser risk of recurrence); hazard ratio greater than 1.0 indicates difference in favor of placebo (higher risk of recurrence with Femara).  1First event of loco-regional recurrence, distant relapse, contralateral breast cancer or death from any cause.  2Analysis stratified by receptor status, nodal status and prior adjuvant chemotherapy (stratification factors as at randomization). *P*-value based on stratified log-rank test. | | | | |

- 1. **Updated Analyses of Extended Adjuvant Treatment of Early Breast Cancer, Median Treatment Duration of 60 Months**

**Table 10: Update of Extended Adjuvant Study Results**

|  | **Femara N = 2582 (%)** | **Placebo N = 2586 (%)** | **Hazard Ratio1**  **(95% CI)** | ***P*-Value2** |
| --- | --- | --- | --- | --- |
| **Disease Free Survival (DFS) events3** | 344 (13.3) | 402 (15.5) | 0.89 (0.77, 1.03) | 0.12 |
| **Breast cancer recurrence**  (Protocol definition of DFS events4) | 209 | 286 | 0.75 (0.63, 0.89) | 0.001 |
| Local Breast Recurrence | 15 | 44 |  |  |
| Local Chest Wall Recurrence | 6 | 14 |  |  |
| Regional Recurrence | 10 | 8 |  |  |
| Distant Recurrence | 140 | 167 |  |  |
| Distant Recurrence (first or subsequent events) Contralateral Breast Cancer | 142  37 | 169  53 | 0.88 (0.70,1.10) | 0.246 |
| Deaths Without Recurrence or Contralateral Breast Cancer | 135 | 116 |  |  |

1Adjusted by receptor status, nodal status and prior chemotherapy

2Stratified log-rank test, stratified by receptor status, nodal status and prior chemotherapy

3DFS events defined as earliest of loco-regional recurrence, distant metastasis, contralateral breast cancer or death from any cause, and ignoring switches to Femara in 60% of the placebo arm.

4Protocol definition does not include deaths from any cause

Updated analyses were conducted at a median follow-up of 62 months. In the Femara arm, 71% of the patients were treated for a least 3 years and 58% of patients completed at least 4.5 years of extended adjuvant treatment. After the unblinding of the study at a median follow-up of 28 months, approximately 60% of the selected patients in the placebo arm opted to switch to Femara.

In this updated analysis shown in Table 10 Femara significantly reduced the risk of breast cancer recurrence or contralateral breast cancer compared with placebo (HR 0.75; 95% CI 0.63, 0.89; *P*=0.001). However, in the updated DFS analysis (interval between randomization and earliest event of loco-regional recurrence, distant metastasis, contralateral breast cancer, or death from any cause) the treatment difference was heavily diluted by 60% of the patients in the placebo arm switching to Femara and accounting for 64% of the total placebo patient-years of follow-up. Ignoring these switches, the risk of DFS event was reduced by a non-significant 11% (HR 0.89; 95% CI 0.77, 1.03). There was no significant difference in distant disease-free survival or overall survival.

# First-Line Treatment of Advanced Breast Cancer

A randomized, double-blind, multinational trial (P025) compared Femara 2.5 mg with tamoxifen 20 mg in 916 postmenopausal patients with locally advanced (Stage IIIB or loco-regional recurrence not amenable to treatment with surgery or radiation) or metastatic breast cancer. Time to progression (TTP) was the primary endpoint of the trial. Selected baseline characteristics for this study are shown in Table 11.

# Table 11: Selected Study Population Demographics

| **Baseline Status** | **Femara** | **Tamoxifen** |
| --- | --- | --- |
|  | **N=458** | **N=458** |
| **Stage of Disease** |  |  |
| IIIB | 6% | 7% |
| IV | 93% | 92% |
| **Receptor Status** |  |  |
| ER and PgR Positive | 38% | 41% |
| ER or PgR Positive | 26% | 26% |
| Both Unknown | 34% | 33% |
| ER- or PgR-/Other Unknown | <1% | 0 |
| **Previous Antiestrogen Therapy** |  |  |
| Adjuvant | 19% | 18% |
| None | 81% | 82% |
| **Dominant Site of Disease** |  |  |
| Soft Tissue | 25% | 25% |
| Bone | 32% | 29% |
| Viscera | 43% | 46% |

Femara was superior to tamoxifen in TTP and rate of objective tumor response (see Table 12).

Table 12 summarizes the results of the trial, with a total median follow-up of approximately 32 months. (All analyses are unadjusted and use 2-sided *P*-values.)

# Table 12: Results of First-Line Treatment of Advanced Breast Cancer

| **Femara** | **Tamoxifen** | | | **Hazard or Odds** |  |  |  |
| --- | --- | --- | --- | --- | --- | --- | --- |
|  | **2.5 mg** | | | **20 mg** | **Ratio (95% CI)** | | |
|  | **N=453** | | | **N=454** | ***P*-Value (2-Sided)** | | |
| **Median Time to Progression** | 9.4 months | | | 6.0 months | 0.72 (0.62, 0.83)1 | | |
|  |  | | |  | *P* <0.0001 | | |
| **Objective Response Rate** |  | | |  |  | | |
| (CR + PR) | 145 (32%) | | | 95 (21%) | 1.77 (1.31, 2.39)2 | | |
|  |  | | |  | *P*=0.0002 | | |
| (CR) | 42 (9%) | | | 15 (3%) | 2.99 (1.63, 5.47)2 | | |
|  |  | | |  | *P*=0.0004 | | |
| **Duration of Objective Response** |  | | |  |  | | |
| Median | 18 months | | | 16 months |  | | |
|  | (N=145) | | | (N=95) |  | | |
| **Overall Survival** | 35 months | | | 32 months |  | | |
|  | (N=458) | | | (N=458) | *P*=0.51363 | | |

1Hazard ratio

2Odds ratio

3Overall log-rank test

Figure 2 shows the Kaplan-Meier curves for TTP.

# Figure 2: Kaplan-Meier Estimates of Time to Progression (Study P025)


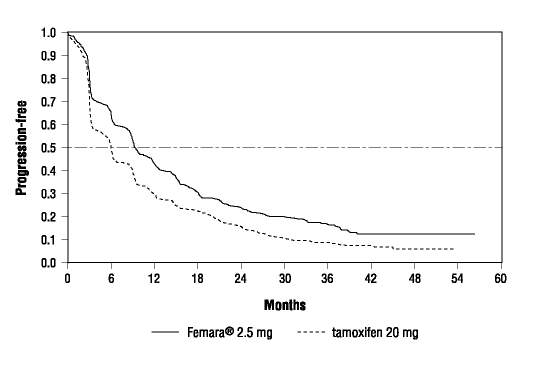


Table 13 shows results in the subgroup of women who had received prior antiestrogen adjuvant therapy, Table 14, results by disease site and Table 15, the results by receptor status.

# Table 13: Efficacy in Patients Who Received Prior Antiestrogen Therapy

| **Variable** | **Femara** | **Tamoxifen** |
| --- | --- | --- |
|  | **2.5 mg** | **20 mg** |
|  | **N=84** | **N=83** |
| **Median Time to Progression** (95% CI) | 8.9 months (6.2, 12.5) | 5.9 months (3.2, 6.2) |
| Hazard Ratio for TTP (95% CI) | 0.60 (0.43, 0.84) | |
| **Objective Response Rate** |  |  |
| (CR + PR) | 22 (26%) | 7 (8%) |
| Odds Ratio for Response (95% CI) | 3.85 (1.50, 9.60) | |

Hazard ratio less than 1 or odds ratio greater than 1 favors Femara; hazard ratio greater than 1 or odds ratio less than 1 favors tamoxifen.

**Table 14: Efficacy by Disease Site**

|  | **Femara** | **Tamoxifen** |
| --- | --- | --- |
|  | **2.5 mg** | **20 mg** |
| **Dominant Disease Site** |  |  |
| **Soft Tissue:** | N=113 | N=115 |
| Median TTP | 12.1 months | 6.4 months |
| Objective Response Rate | 50% | 34% |
| **Bone:** | N=145 | N=131 |
| Median TTP | 9.5 months | 6.3 months |
| Objective Response Rate | 23% | 15% |
| **Viscera:** | N=195 | N=208 |
| Median TTP | 8.3 months | 4.6 months |
| Objective Response Rate | 28% | 17% |

**Table 15: Efficacy by Receptor Status**

| **Variable** | **Femara** | **Tamoxifen** |
| --- | --- | --- |
|  | **2.5 mg** | **20 mg** |
| **Receptor Positive** | N=294 | N=305 |
| Median Time to Progression (95% CI) | 9.4 months (8.9, 11.8) | 6.0 months (5.1, 8.5) |
| Hazard Ratio for TTP (95% CI) | 0.69 (0.58, 0.83) |  |
| Objective Response Rate (CR+PR) | 97 (33%) | 66 (22%) |
| Odds Ratio for Response 95% CI) | 1.78 (1.20, 2.60) |  |
| **Receptor Unknown** | N=159 | N=149 |
| Median Time to Progression (95% CI) | 9.2 months (6.1, 12.3) | 6.0 months (4.1, 6.4) |
| Hazard Ratio for TTP (95% CI) | 0.77 (0.60, 0.99) |  |
| Objective Response Rate (CR+PR) | 48 (30%) | 29 (20%) |
| Odds Ratio for Response (95% CI) | 1.79 (1.10, 3.00) |  |

Hazard ratio less than 1 or odds ratio greater than 1 favors Femara; hazard ratio greater than 1 or odds ratio less than 1 favors tamoxifen.

Figure 3 shows the Kaplan-Meier curves for survival.

# Figure 3: Survival by Randomized Treatment Arm


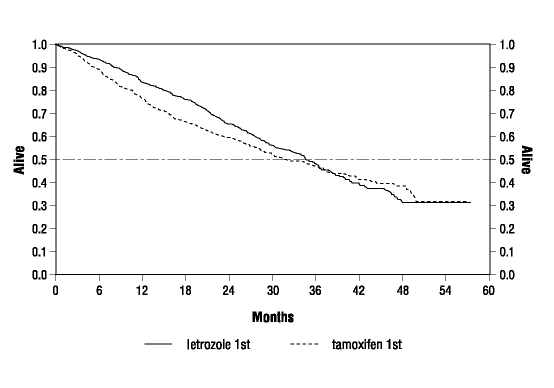


**Legend:** Randomized Femara: n=458, events 57%, median overall survival 35 months (95% CI 32 to 38 months) Randomized tamoxifen: n=458, events 57%, median overall survival 32 months (95% CI 28 to 37 months) Overall log-rank *P*=0.5136 (i.e., there was no significant difference between treatment arms in overall survival).

The median overall survival was 35 months for the Femara group and 32 months for the tamoxifen group, with a *P*-value 0.5136. Study design allowed patients to cross over upon progression to the other therapy. Approximately 50% of patients crossed over to the opposite treatment arm and almost all patients who crossed over had done so by 36 months. The median time to crossover was 17 months (Femara to tamoxifen) and 13 months (tamoxifen to Femara). In patients who did not cross over to the opposite treatment arm, median survival was 35 months with Femara (n=219, 95% CI 29 to 43 months) vs 20 months with tamoxifen (n=229, 95% CI 16 to 26 months).

# Second-Line Treatment of Advanced Breast Cancer

Femara was initially studied at doses of 0.1 mg to 5.0 mg daily in six noncomparative trials (AR/BC1, P01, AR/ST1, AR/PS1, AR/ES1 and NJO-03) in 181 postmenopausal estrogen/progesterone receptor positive or unknown advanced breast cancer patients previously treated with at least antiestrogen therapy. Patients had received other hormonal therapies and also may have received cytotoxic therapy. Eight (20%) of forty patients treated with Femara 2.5 mg daily in trials achieved an objective tumor response (complete or partial response).

Two large randomized, controlled, multinational (predominantly European) trials (AR/BC2, AR/BC3) were conducted in patients with advanced breast cancer who had progressed despite antiestrogen therapy. Patients were randomized to Femara 0.5 mg daily, Femara

2.5 mg daily, or a comparator [megestrol acetate 160 mg daily in one study (AR/BC2); and aminoglutethimide 250 mg twice a day with corticosteroid supplementation in the other study (AR/BC3)]. In each study over 60% of the patients had received therapeutic antiestrogens, and about one-fifth of these patients had an objective response. The megestrol acetate controlled study was double- blind; the other study was open label. Selected baseline characteristics for each study are shown in Table 16.

# Table 16: Selected Study Population Demographics

| **Parameter** | **megestrol acetate** | **aminoglutethimide** |
| --- | --- | --- |
|  | **study** | **study** |
| **No. of Participants** | 552 | 557 |
| **Receptor Status** |  |  |
| ER/PR Positive | 57% | 56% |
| ER/PR Unknown | 43% | 44% |
| **Previous Therapy** |  |  |
| Adjuvant Only | 33% | 38% |
| Therapeutic +/- Adj. | 66% | 62% |
| **Sites of Disease** |  |  |
| Soft Tissue | 56% | 50% |
| Bone | 50% | 55% |
| Viscera | 40% | 44% |

Confirmed objective tumor response (complete response plus partial response) was the primary endpoint of the trials. Responses were measured according to the Union Internationale Contre le Cancer (UICC) criteria and verified by independent, blinded review. All responses were confirmed by a second evaluation 4 to 12 weeks after the documentation of the initial response.

Table 17 shows the results for the first trial (AR/BC2), with a minimum follow-up of 15 months, which compared Femara 0.5 mg, Femara 2.5 mg, and megestrol acetate 160 mg daily. (All analyses are unadjusted.)

# Table 17: Megestrol Acetate Study Results

|  | **Femara** | **Femara** | **megestrol** |
| --- | --- | --- | --- |
|  | **0.5 mg** | **2.5 mg** | **acetate** |
|  | **N=188** | **N=174** | **N=190** |
| **Objective Response (CR + PR)** | 22 (11.7%) | 41 (23.6%) | 31 (16.3%) |
| **Median Duration of Response** | 552 days | (Not reached) | 561 days |
| **Median Time to Progression** | 154 days | 170 days | 168 days |
| **Median Survival** | 633 days | 730 days | 659 days |
| **Odds Ratio for Response** | Femara 2.5: Femara 0.5=2.33 | | Femara 2.5: megestrol=1.58 |
|  | (95% CI: 1.32, 4.17); *P*=0.004* | | (95% CI: 0.94, 2.66); *P*=0.08* |
| **Relative Risk of Progression** | Femara 2.5: Femara 0.5=0.81 | | Femara 2.5: megestrol=0.77 |
|  | (95% CI: 0.63, 1.03); *P*=0.09* | | (95% CI: 0.60, 0.98); *P*=0.03* |


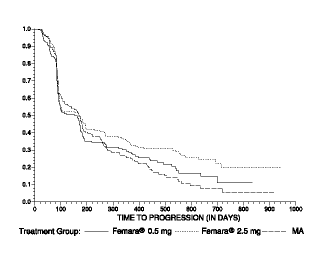
*Two-sided *P*-value

The Kaplan-Meier curves for progression for the megestrol acetate study are shown in Figure 4.

Figure 4: Kaplan-Meier Estimates of Time to Progression (Megestrol Acetate Study).

The results for the study comparing Femara to aminoglutethimide (AR/BC3), with a minimum follow-up of 9 months, are shown in Table 18 (Unadjusted analyses are used).

# Table 18: Aminoglutethimide Study Results

|  | **Femara** | **Femara** |  |
| --- | --- | --- | --- |
|  | **0.5 mg** | **2.5 mg** | **aminoglutethimide** |
|  | **N=193** | **N=185** | **N=179** |
| **Objective Response (CR + PR)** | 34 (17.6%) | 34 (18.4%) | 22 (12.3%) |
| **Median Duration of Response** | 619 days | 706 days | 450 days |
| **Median Time to Progression** | 103 days | 123 days | 112 days |
| **Median Survival** | 636 days | 792 days | 592 days |
| **Odds Ratio for Response** | Femara 2.5: |  | Femara 2.5: |
|  | Femara 0.5=1.05 |  | aminoglutethimide=1.61 |
|  | (95% CI: 0.62, 1.79); *P*=0.85* | | (95% CI: 0.90, 2.87); *P*=0.11* |
| **Relative Risk of Progression** | Femara 2.5: |  | Femara 2.5: |
|  | Femara 0.5=0.86 |  | aminoglutethimide=0.74 |
|  | (95% CI: 0.68, 1.11); *P*=0.25* | | (95% CI: 0.57, 0.94); *P*=0.02* |

*Two-sided *P*-value

The Kaplan-Meier curves for progression for the aminoglutethimide study is shown in Figure 5.

# Figure 5: Kaplan-Meier Estimates of Time to Progression (Aminoglutethimide Study)


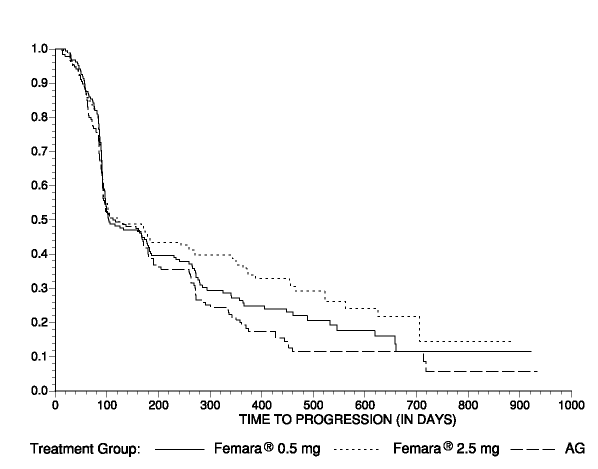


1. **HOW SUPPLIED/STORAGE AND HANDLING**

Packaged in HDPE bottles with a safety screw cap.

2.5 mg tablets

Bottles of 30 tablets...................................................................................NDC 0078-0249-15

Store at 25°C (77°F); excursions permitted to 15°C to 30°C (59°F to 86°F) [see USP Controlled Room Temperature].

# PATIENT COUNSELING INFORMATION

### Embryo-Fetal Toxicity

Advise females of reproductive potential of the potential risk to a fetus and to use effective contraception during Femara therapy and for at least 3 weeks after the last dose. Advise females to contact their healthcare provider if they become pregnant, or if pregnancy is suspected, during treatment with Femara *[see Warnings and Precautions (5.6) and Use in Specific Populations (8.1, 8.3)].*

### Lactation

Advise women not to breastfeed during Femara treatment and for at least 3 weeks after the last dose *[see Use in Specific Populations (8.2)].*

### Infertility

Advise females and males of reproductive potential of the potential for reduced fertility from Femara *[see Use in Specific Populations (8.3)].*

### Fatigue and Dizziness

Since fatigue and dizziness have been observed with the use of Femara and somnolence was uncommonly reported, caution is advised when driving or using machinery.

### Bone Effects

Consideration should be given to monitoring bone mineral density. Distributed by:

Novartis Pharmaceuticals Corporation

East Hanover, New Jersey, 07936

© Novartis

T2017-106
